# Hydrophobic tuning with non-canonical amino acids in a copper metalloenzyme

**DOI:** 10.1038/s41557-026-02116-7

**Published:** 2026-04-13

**Authors:** Sandro Fischer, Anton Natter Perdiguero, Kelvin Lau, Alexandria Deliz Liang

**Affiliations:** 1 Department of Chemistry, University of Zurich, Zurich, Switzerland; 2 Protein Production and Structure Characterization Core Facility (PTPSP), School of Life Sciences, École Polytechnique Fédérale de Lausanne (EPFL), Lausanne, Switzerland

**Keywords:** Biocatalysis, Expression systems, Metalloproteins, Synthetic biology, Enzymes

## Abstract

Hydrophobicity controls many aspects of protein and enzyme function. Although hydrophobic tuning can be somewhat achieved with canonical amino acids, the incorporation of non-canonical amino acids further extends this ability to enable new and improved functionality. Here we engineer an aminoacyl-tRNA synthetase/tRNA pair for the site-specific genetic encoding of a set of bulky, hydrophobic amino acids, namely cyclopentylalanine, cyclohexylalanine and cycloheptylalanine. As a proof of concept, we demonstrate the utility of hydrophobic tuning based on non-canonical amino acids (ncAAs) to engineer a bacterial laccase, which is both a classical metalloenzyme and a high-value catalyst for industrial processes. The resulting mutations substantially improved the catalytic activity, particularly the turnover frequency and total turnover number. To understand this improved functionality, the redox potentials, electronic spectra and structure–function relationships were examined. Combining traditional directed evolution with ncAA-based engineering resulted in further improvements in catalysis, which were contextualized by analysing the changes imparted from these two methods.

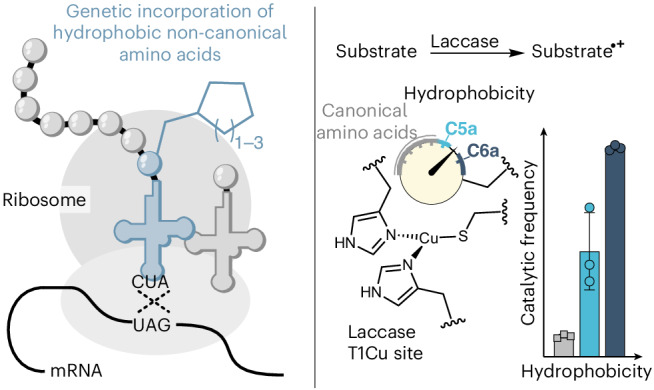

## Abstract (web summary)

Hydrophobicity plays an important role in protein function, but tuning hydrophobicity with canonical amino acids is chemically limited. Now through the genetic incorporation of bulky, highly hydrophobic non-canonical amino acids, their utility in enzyme engineering by enhancing the function of a copper-dependent laccase through hydrophobic tuning has been demonstrated.

## Main

Hydrophobic interactions are crucial for stabilizing protein cores and driving folding. Within enzymes, hydrophobic environments can also promote catalysis and tune cofactor binding, substrate binding and reactivity^1,2^. In the laboratory, modifications of such environments by programmed mutagenesis have demonstrated both chemically intuitive and surprising effects. For metalloenzymes in particular, hydrophobic environments can affect a plethora of properties, including redox potential^3^, the acidity of metal-coordinated water molecules^4^ and even metal specificity^5,6^. Nature and traditional laboratory mutagenesis use predominantly 20 canonical amino acids to optimize or study these effects. However, incorporation of non-canonical amino acids (ncAAs) allows for broadened chemical variability, enabling detailed enzymatic studies^7–10^, novel reactions^11–14^ and even improved function^15–18^. Hydrophobic tuning using ncAAs has been demonstrated with proteins generated through native chemical ligation^3,19^, in vitro protein production with pre-aminoacylated transfer RNAs (tRNAs)^20^ and global ncAA replacement of methionine with norleucine or homopropargylglycine^21–23^. However, these techniques are limited compared with the genetic incorporation of ncAAs in a site-specific manner. Such limitations include low protein yield, the need for peptide synthesis using harmful solvents and reagents, limited applicability to diverse protein scaffolds and a lack of targeted mutagenesis, preventing (semi-)rational engineering.

Despite the value of site-specific, genetic ncAA incorporation, this strategy remains underexplored for hydrophobic tuning, largely owing to the limited available tools. Herein we describe the development of a platform for the robust genetic incorporation of three extremely hydrophobic ncAAs and application of these tools in a proof-of-concept study to fine-tune the hydrophobic environment of a high-value enzyme—a bacterial laccase (Enzyme Commission number 1.10.3.2). Our results demonstrate that site-selective incorporation of either l-3-cyclopentylalanine (**C5a**) or l-3-cyclohexylalanine (**C6a**) at a key position in the active site of a bacterial laccase improved the turnover frequency (*k*_cat_), catalytic efficiency (*k*_cat_/*K*_M_, where *K*_M_ is the Michaelis constant) and total turnover number (TTN), particularly with more-challenging high-redox-potential substrates. Moreover, we show that these parameters can be further enhanced through traditional directed evolution, and we compare the outcomes of these two strategies for enzyme engineering. These results highlight that site-specific incorporation of ncAAs can be used to improve catalysis, tune the hydrophobicity of a protein environment and directly promote the rate-limiting step(s) of an enzyme.

## Results

### Cycloalkyl hydrophobic ncAAs can be genetically incorporated

**Fig. 1:**
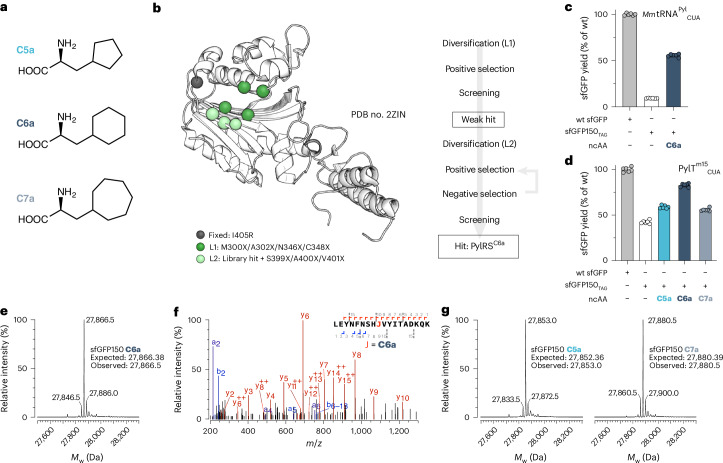
Engineering an aaRS/tRNA pair for bulky hydrophobic ncAAs. **a**, Structures of ncAAs incorporated in this study: **C5a**, **C6a** and **C7a**. **b**, Mutation positions within the catalytic domain of *Mm*PylRS, PDB no. 2ZIN (ref. ^30^) for site libraries used for engineering PylRS^C6a^. **c**, Amber suppression benchmarking of pGS1T-PylRS^C6a^–*Mm*tRNA^Pyl^_CUA_ pair in sfGFP150_TAG_ with and without 6 mM **C6a**. The data are normalized to an otherwise wt protein (wt sfGFP) and represent the mean and standard deviation of six biological replicates. **d**, Amber suppression benchmarking of the pGS1T-PylRS^C6a^–PylT^m15^_CUA_ pair in sfGFP150_TAG_ with and without 12 mM dl-**C5a**, 6 mM **C6a** or 12 mM dl-**C7a**. The data are normalized to an otherwise wt protein (wt sfGFP) and represent the mean and standard deviation of six biological replicates. **e**, Intact LC-MS analysis (positive electrospray time of flight) of sfGFP150_TAG_ expression in the presence of 6 mM **C6a**. Additional peaks consistent with typically observed H_2_O elimination (–18 Da) and Na^+^ addition (+23 Da) are labelled. **f**, A representative LC-MS/MS spectrum from a tryptic digest of sfGFP150_TAG_ expressed with pGS1T-PylRS^C6a^–PylT^m15^_CUA_ in the presence of 6 mM **C6a**. The peptide fragmentation diagram indicates the fragment ions identified from MS/MS fragmentation. Multiple peptides containing **C6a** were observed, but no peptides for canonical amino acid incorporation were observed. *m*/*z*, the mass-to-charge ratio. **g**, Intact LC-MS analysis (positive electrospray time of flight) of sfGFP150_TAG_ expression in the presence of 12 mM dl-**C5a** or 12 mM dl-**C7a**. Additional peaks consistent with typically observed H_2_O elimination (–18 Da) and Na^+^ addition (+23 Da) are labelled. The full MS spectra for insets in **e** and **g** are supplied in Supplementary Fig. 2. Source data

**Extended Data Fig. 1:**
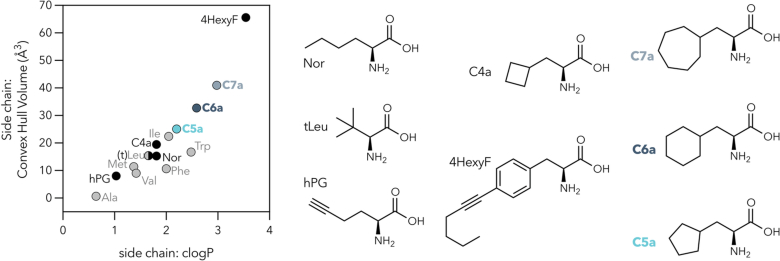
Comparison of hydrophobic amino acids: volumes and calculated n-octanol:water partition coefficients (clogP). **C5a**, **C6a**, and **C7a** have high predicted n-octanol:water partition coefficients (clogP), indicating high hydrophobicity, and they are distinctly more space-filing than the hydrophobic canonical amino acids. The comparison also illustrates that these ncAAs expand the chemical space for these metrics substantially more than the canonical amino acids (grey) and more than other ncAAs (black), such as cyclobutylalanine (C4a), norleucine (Nor), tert-leucine (tLeu), and homopropargylglycine (hPG). A recently reported set of ncAAs that are lipidation mimics (such as 4HexyF) are also highly hydrophobic^114^ and space-filling, but their long side chains make them less suitable for engineering within enzyme active sites, which are typically buried within a protein. Because the calculated parameters are the same for Leu and tLeu, they are shown as half circles of grey and black. Source data

Based on an assessment of hydrophobicity and size, we identified three bulky, hydrophobic ncAAs—l-3-cyclopentylalanine (**C5a**), l-3-cyclohexylalanine (**C6a**) and l-3-cycloheptylalanine (**C7a**)—with substantially distinct features compared with canonical hydrophobic amino acids (Fig. 1a and Extended Data Fig. 1). We reasoned that these differences would make them ideal candidates to augment the natural amino acid alphabet. Additionally, we note that other groups have also reported these ncAAs as valuable for protein and peptide applications, but no tools exist for their genetic incorporation in vivo^20,24–28^. Site-specific genetic incorporation of ncAAs in cells requires the development of an aminoacyl-tRNA synthetase (aaRS)/tRNA pair that specifically recognizes a ncAA and enables recoding of a target codon via the ribosome. Typically, the target codon is the amber stop codon (TAG). Due to the nature of aaRS/tRNA engineering, many evolved aaRS/tRNA pairs display polyspecificity, enabling incorporation of related ncAAs using the same aaRS/tRNA pair. Herein, to maximize the potential of observing polyspecificity, we engineered an aaRS/tRNA pair for incorporation of **C6a**, the intermediate-sized ncAA.

Based on its evolvability and its orthogonality in several common laboratory hosts, the pyrrolysyl-tRNA synthetase (PylRS)/tRNA pair was selected as the parent system for engineering^29^. The PylRS from *Methanosarcina mazei* (*Mm*PylRS; Protein Data Bank (PDB) no. 2ZIN; ref. ^30^) was subjected to two rounds of randomization using site-saturation mutagenesis (Fig. 1b). Positive selection, negative selection and screening were conducted iteratively with co-expression of the cognate tRNA for amber suppression (*Mm*tRNA^Pyl^_CUA_). The best performing mutant, termed PylRS^C6a^, contained five amino acid mutations: M300D/L302H/N346A/A400C/I405R. The efficiency of this mutant for **C6a** incorporation was evaluated using a superfolder green fluorescent protein (sfGFP) reporter containing an amber stop codon at position N150 (sfGFP150_TAG_). The production of sfGFP150_TAG_ increased approximately sixfold upon addition of 6 mM **C6a** (Supplementary Fig. 1).

For application of engineered aaRS/tRNA pairs, we designed an optimized plasmid system (pGS1T). The PylRS^C6a^/*Mm*tRNA^Pyl^_CUA_ pair was cloned onto pGS1T, where PylRS^C6a^ is under the control of a constitutive glnS promoter and *Mm*tRNA^Pyl^_CUA_ is under the control of a proK promoter. In the optimized plasmid system, expression of sfGFP150_TAG_ from a pBAD vector in NEB10β competent *Escherichia coli* (New England Biolabs) showed an approximately sixfold increase upon addition of 6 mM **C6a**, yielding >50% of wild-type protein (wt sfGFP) production (Fig. 1c). To further improve the suppression efficiency, *Mm*tRNA^Pyl^_CUA_ was replaced with PylT^m15^_CUA_, a previously reported mutant tRNA derived from *Mm*tRNA^Pyl^_CUA_ (ref. ^31^). In the presence of 6 mM **C6a**, the resulting construct improved the incorporation efficiency of **C6a** in sfGFP150_TAG_ to result in high levels of sfGFP production (>80% of wt protein) with selective incorporation of **C6a** as shown by liquid-chromatography/mass-spectrometry (LC-MS) and LC/tandem-MS (LC-MS/MS; Fig. 1d–f). Importantly, although multiple peptides containing **C6a** were observed, no peptides for canonical amino acids were observed. In the absence of **C6a** with PylT^m15^_CUA_, incorporation of phenylalanine was observed. To examine if we could leverage polyspecificity to incorporate **C5a** and **C7a**, we tested the PylRS^C6a^/PylT^m15^_CUA_ pair in the presence and absence of **C5a** and **C7a**. We observed a modest increase in sfGFP production upon addition of 12 mM dl-ncAA for both ncAAs (Fig. 1d), but the LC-MS data of both purified proteins show that the ncAA was selectively incorporated without incorporation of phenylalanine (Fig. 1g). Thus we conclude that in the presence of the desired ncAA, the engineered PylRS^C6a^/PylT^m15^_CUA_ is capable of selective incorporation of **C5a**, **C6a** or **C7a** into proteins in *E. coli*. Alternatively, if selectivity in the absence of the ncAA is required, the engineered PylRS^C6a^/*Mm*tRNA^Pyl^_CUA_ pair is a highly suitable alternative, albeit with lower yields.

### Bacterial laccases as a proof of concept for hydrophobic tuning

**Fig. 2:**
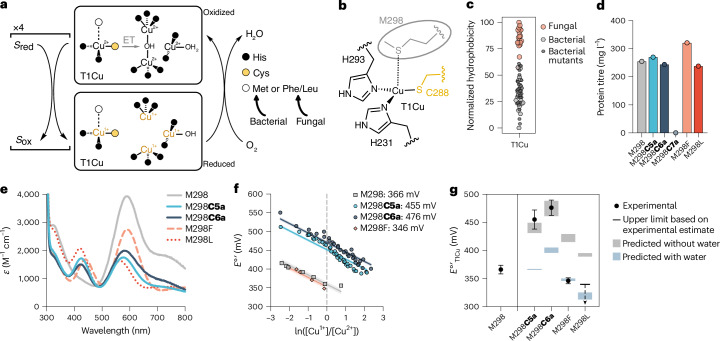
Laccases, general reactivity with features of the copper centres and *Sc*SLAC variant characterization for M298, M298C5a, M298C6a, M298C7a, M298F and M298L. **a**, General copper coordination and catalysis of *Sc*SLAC, in which electron transfer (ET) occurs from the reduced substrate (*S*_red_) to the oxidized T1Cu centre, producing the one-electron oxidized substrate (*S*_ox_). This process is repeated four times, with electrons flowing from the T1Cu to the trinuclear copper centre to form the four-electron reduced enzyme state, which catalyses the reduction of molecular oxygen to water. **b**, Chemical representation of the T1Cu centre responsible for substrate oxidation. **c**, Comparison of the average hydrophobicity within 10 Å of the T1Cu centre in fungal, bacterial and mutant bacterial laccases. **d**, Expression titre of *Sc*SLAC variants in milligrams per litre of culture from a single, representative expression. For each variant, the proteins were isolated as biological replicates at least three times. **e**, Absorption spectra of *Sc*SLAC variants, in which *ε* represents the extinction coefficient. **f**, Spectrophotometric redox titration analysis to determine the T1Cu redox potential (*E*°′_T1Cu_) through calculating the ratio of oxidized to reduced T1Cu species at varying potentials. The potential of M298L was too low to be determined by the spectrophotometric method; only an upper limit of 340 mV could be estimated. **g**, Comparison of predicted and experimentally determined redox potentials based on the change in hydrophobicity^3^. We conducted *E*°′_T1Cu_ predictions accounting for hydrophobicity changes from the M298 mutation as well as predictions accounting for both this mutation and the accumulation of water. The error bars indicate standard deviation. For M298L, the experimentally determined upper limited is provided. Source data

We next sought to demonstrate the power of hydrophobic tuning with a long-standing, well-documented challenge as a proof of concept: laccases. These multi-copper enzymes are capable of oxidizing diverse substrates, concomitantly reducing molecular oxygen to water^32,33^, making them ‘green’ catalysts for a wide variety of applications from biomass valorization^34,35^ to synthetic chemistry^36,37^. Laccases contain two distinct copper sites: (1) a prototypical mononuclear T1Cu site that oxidizes substrates and (2) a trinuclear copper cluster (TNC) that reduces molecular oxygen, comprising a T2Cu site and a binuclear T3Cu centre^32^ (Fig. 2a). The T1Cu site is located near the surface of the protein, and its substrate-binding pocket is open and solvent-exposed, accommodating diverse chemical structures and enabling a broad substrate scope. The rate-limiting step of laccases is often characterized as electron transfer from the substrate to the T1Cu centre^32,38–44^, which is governed by the redox potential of the T1Cu site (*E*°′_T1Cu_), reorganization energy, electronic coupling, electron transfer distance and temperature according to Marcus theory^45^. Fungal laccases and bacterial laccases are highly dissimilar in three-dimensional (3D) structure but contain similar coordination of the T1Cu site and the TNC. Fungal laccases are typically more-potent catalysts with higher *E*°′_T1Cu_ values (approximately +700 mV versus the normal hydrogen electrode (NHE)^46^), but they also exhibit reduced stability, require complex glycosylation patterns for function and are challenging to express in heterologous hosts^47–49^. By contrast, bacterial laccases can be rapidly produced with high yield, are active under harsh conditions and do not require glycosylation. However, bacterial laccases are less applicable in many domains because of their diminished activity, which is often linked to their low *E*°′_T1Cu_ (approximately +360 to +400 mV versus the NHE; ref. ^47^).

One of the most thoroughly discussed differences between bacterial and fungal laccases is the coordination at the T1Cu site. Bacterial laccases contain a CysHis_2_Met motif (Fig. 2b), whereas fungal laccases lack the coordinating methionine, which is replaced by a non-coordinating phenylalanine or leucine residue. The difference in *E*°′_T1Cu_ is often attributed to the axial methionine: the elongated MetS–Cu bond increases the electron density at the copper centre through the additional coordination, stabilizing the Cu^2+^ state, destabilizing the Cu^1+^ state and, therefore, lowering the *E*°′_T1Cu_. Several attempts have been made to increase the *E*°′_T1Cu_ and activity of bacterial laccases by engineering the primary coordination sphere of the T1Cu site to resemble the T1Cu site of fungal laccases^38,39,50–55^. These classical mutagenesis methods have in some cases been shown to increase the *E*°′_T1Cu_ value^56,57^, but concurrently, their rate-limiting kinetics are typically substantially reduced. A notable exception is the unusual CueO multi-copper oxidase, in which a fifth copper binds near the T1Cu site^52,58^. The general challenges associated with these classical mutagenesis strategies highlight the need for alternative methods.

Our analysis of the T1Cu sites of bacterial and fungal laccases indicated that the T1Cu sites of fungal laccases are markedly more hydrophobic than the T1Cu sites of bacterial laccases (Fig. 2c). Based on this information, fundamental chemical knowledge and prior reports with laccases, we posited that the bacterial laccase from *Streptomyces coelicolor* (*Sc*SLAC) could benefit from hydrophobic tuning with our ncAAs to increase the *E*°′_T1Cu_, improve the rate-limiting step and overcome limitations encountered when engineering the primary coordination sphere of the T1Cu environment^38–42,56–58^.

We postulated that mutation of the axial methionine to **C5a**, **C6a** or **C7a** would remove electron density contributed from the coordinating methionine at the copper centre, increase hydrophobicity, provide steric bulk and unlike mutations of the axial methionine to canonical amino acids, prevent water from accumulating near the T1Cu site, all potentially raising the *E*°′_T1Cu_ and improving the rate-limiting step of catalysis. Notably, recent work has also linked the accumulation of water near the T1Cu centre to the diminished activity of axial mutants in *Sc*SLAC and a related bacterial laccase^42,55^. We anticipated that increases up to approximately +300 mV—nearing that of the fungal laccases—would likely be tolerated without making the subsequent catalytic steps prohibitively slow or causing a significant thermodynamic penalty. Thus we designed experiments to incorporate **C5a**, **C6a** and **C7a** into the axial position of the T1Cu site in *Sc*SLAC (M298; Fig. 2b).

### Replacing the axial methionine with C5a or C6a increases the *E*°′_T1Cu_

Using the engineered PylRS^C6a^/PylT^m15^_CUA_ in the strain B-95ΔAΔfabR (refs. ^59,60^), we produced M298**C5a** and M298**C6a** with a high, wt-like titre, but **C7a** yielded no soluble protein, possibly owing to steric bulk (Fig. 2d). Additionally, we isolated wt *Sc*SLAC and the high-redox potential mimics—mutants M298F and M298L—to compare the properties of these key mutations. For each protein variant, we confirmed the expected mass of the intact protein by MS (Supplementary Fig. 3) and determined the copper content by atomic absorption spectroscopy (Supplementary Fig. 4). Additionally, as described below, we evaluated the ultraviolet–visible (UV–vis) absorption spectra, electron paramagnetic resonance (EPR) spectra and the redox potential by spectrophotometric titrations.

For all four M298 mutants, the UV–vis spectra of the oxidized Cu^2+^ state exhibited a lower-intensity ligand-to-metal charge-transfer (LMCT) band at ~600 nm and higher-intensity LMCT band at ~450 nm (Fig. 2e and Supplementary Table 1). Within T1Cu centres, these bands are attributed to a *π*-bonding interaction (~600 nm) and a *σ*-bonding interaction (~450 nm) between the sulfur *p* orbitals of the cysteine and the ${d}_{{x}^{2}-{y}^{2}}$ orbital of the copper ion^56,61^. The intense *σ*-bonding LMCT band is unusual for both natural bacterial and fungal laccases^55,62^. Such LMCT changes are consistent with distortion altering the Cu–S_Cys_ orbital overlap^63–66^, or minimization of the local electric field at the T1Cu centre increasing the covalency of the Cu–S_Cys_ bond^67^.

**Extended Data Fig. 2:**
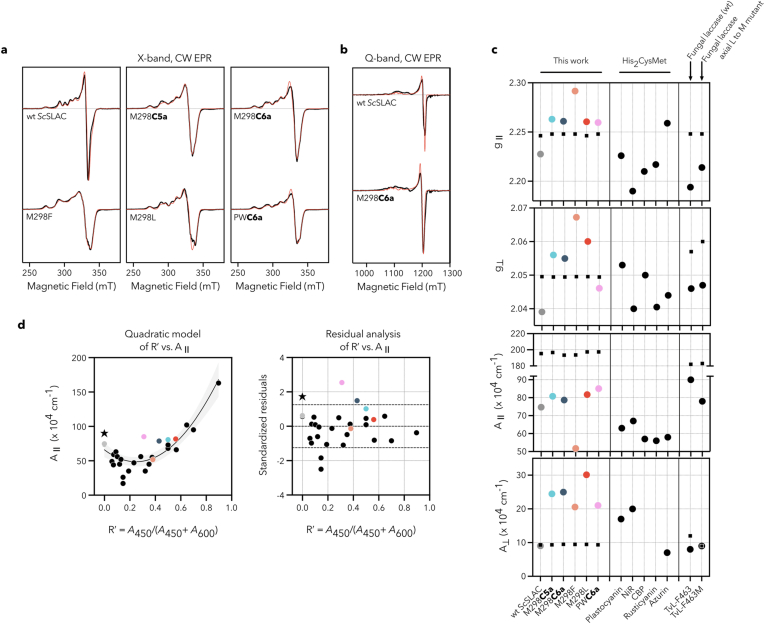
The EPR spectra and parameters for wt *Sc*SLAC, M298C5a, M298C6a, M298F, M298L, and PWC6a. **a**) The X-band continuous-wave (cw) EPR spectra for wt *Sc*SLAC, M298**C5a**, M298**C6a**, M298F, M298L, and PW**C6a** were collected at 40 K (black traces) and simulated as a composite (red traces) of the two EPR-active spin systems T1Cu and T2Cu. **b**) For wt *Sc*SLAC and M298**C6a**, the Q-band EPR spectra were also collected at 40 K and simulated (black and red, respectively). **c**) The EPR parameters derived from the simulation were extracted and visualized for comparison. For comparison, parameters from several T1Cu sites are shown^65^, including both *Tv*L and its axial methionine mutant (*Tv*L-F463M)^40^. The T2Cu site parameters are shown as small black squares. The mutants examined in this work are colored as shown throughout the manuscript (M298, grey; M298**C5a**, cyan; M298**C6a**, dark blue; M298F, salmon; M298L, red; and PW**C6a**, pink). **d**) Modeling of the UV-vis parameter R’ (ε_σ_ /(ε_σ_ + ε_π_)) as a function of the EPR parameter A_∥_ was examined as previously reported^65^. The three-coordinate fungal laccase *Tv*L^40^ is illustrated as a black star. The mutants of in this study are colored as described in panel c. As previously reported, the collective data were well described by a quadratic function, with notable outliers above the modeled function for the three-coordinate fungal laccases, PW**C6a**, and M298**C6a**, as well as outliers below the function for the azurin variants M121G and M121Q. A residual analysis is provided to highlight deviations from the model. The shading in panel c represents the 95% confidence interval for the quadratic fit. Source data

In addition to UV–vis, EPR signals have historically been used to describe the T1Cu centres of different proteins (Extended Data Fig. 2 and Supplementary Table 2). Thus we collected EPR data to compare the variants here with each other and with other previously reported T1Cu centres. The EPR spectra of M298F and M298L deviated from that of wt *Sc*SLAC, and their spectral parameters (parallel and perpendicular *g* values, *g*_⊥_ and *g*_∥_, and the hyperfine coupling constants *A*_⊥_ and *A*_∥_) were consistent with previous studies of fungal-like mutants of SLAC^55,62^. The EPR parameters derived for M298**C5a** and M298**C6a** (*g*_⊥_ = 2.055–2.056, *g*_∥_ = 2.261–2.263, ∆*g* = 0.206 and *A*_∥_ = 79–81 × 10^4^ cm^−1^) are intermediate between those of wt *Sc*SLAC and M298F, and are dissimilar from those of fungal laccases, which typically exhibit lower *g*_∥_ values (2.16–2.21) and higher *A*_∥_ values (85–100 × 10^4^ cm^−1^) than those observed for wt *Sc*SLAC^68,69^. By comparison, with fungal laccases, axial phenylalanine/leucine to methionine mutations shift the EPR parameters, but the EPR parameters of the resulting axial methionine mutants do not match those of bacterial laccases^40,68^ (Extended Data Fig. 2). We evaluated if these variants are consistent with a previously reported model describing a quadratic relationship between *R*′ and *A*_∥_ for four-coordinate T1Cu centres, where *R*′ is the ratio of extinction coefficient for the *σ* LMCT band compared to the sum of the extinction coefficients for the *σ* and *π* LMCT bands (ε_*σ*_/(*ε*_*σ*_ + *ε*_*π*_))^65^. Good agreement was observed for M298, M298F and M298L, but fungal laccases, M298**C6a** and to a lesser extent M298**C5a** exhibited markedly higher *A*_∥_ values than predicted from their *R*′ values (Extended Data Fig. 2). Unlike for UV–vis data^67^, it is unclear how hydrophobicity or electric-field changes could impact T1Cu EPR signals. Interestingly, a dampened electric field from extreme hydrophobic tuning could explain the increase in the LMCT from the *σ*-bonding interaction and increased covalency elevating the *A*_∥_ values.

To further understand changes in the T1Cu electronics, spectrophotometric redox titrations were performed to determine *E*°′_T1Cu_ (Fig. 2f, Supplementary Fig. 5 and Supplementary Table 1). The wt *Sc*SLAC *E*°′_T1Cu_ was determined to be 366(7) mV versus NHE, consistent with literature values between 360 and 430 mV (refs. ^54,70–72^). For the fungal-like *Sc*SLAC mutants M298F and M298L, the *E*°′_T1Cu_ values were lower compared with wt *S*cSLAC, consistent with a previous report of *Sc*SLAC^56^. The potential of M298L was so low that it could not be accurately determined with the method used herein (potassium ferricyanide titration). By contrast, the *E*°′_T1Cu_ values for M298**C5a** and M298**C6a** were substantially higher than that of wt *Sc*SLAC, with an increase of approximately +90 mV and +110 mV, respectively. A model relating *E*°′_T1Cu_ and the axial-residue partition coefficient (log*P*) was applied to predict the shift in *E*°′_T1Cu_ based on hydrophobicity changes^3^. The results indicate that *E*°′_T1Cu_ should shift by approximately +74 and +109 mV for M298**C5a** and M298**C6a**, respectively, which aligns well with our experimental results (Fig. 2g). Conversely, the experimentally determined *E*°′_T1Cu_ values for M298F and M298L are at least 50 mV more negative than predicted. Crystallographic studies reveal water accumulation near the T1Cu site of fungal-like SLAC variants, attributed to the small size of leucine and rotation of the phenyl ring away from the T1Cu site into an adjacent thin pocket^42,55,62^ (Supplementary Fig. 6). For M298F and M298L, accounting for log*P* changes from both the axial side chain and an ordered water molecule results in predictions that align well with our experimentally determined redox potentials (Fig. 2g). Thus, there is likely differential water accumulation between M298F/L and M298**C5a**/**C6a**. Collectively, our spectroelectrochemical results suggest that M298**C5a** and M298**C6a** deviate from natural bacterial and fungal laccases, fungal-like mutants of bacterial laccases and other well-studied T1Cu sites.

### Axial C5a and C6a mutations improve catalysis

**Fig. 3:**
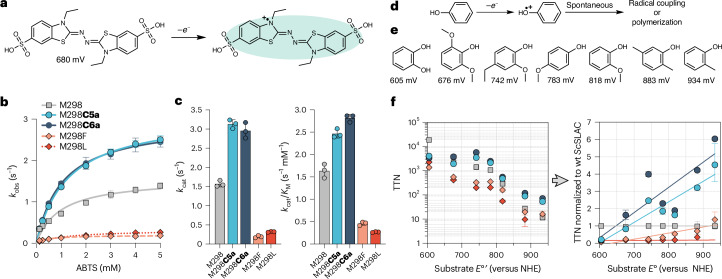
Characterization of steady-state activity and phenolic substrate profiling of *Sc*SLAC variants. **a**, Oxidation reaction of the classical laccase substrate ABTS. **b**, Michaelis–Menten kinetics of *Sc*SLAC variants with ABTS as a substrate at pH 4.0 at 25 °C. *k*_obs_ represents the observed rate constant. **c**, Comparison of extracted kinetic parameters for the *Sc*SLAC variants with ABTS as substrate. **d**, Phenolic oxidation reactions with laccases. **e**, Phenolic substrate panel and reported redox potentials versus NHE^74^. **f**, TTN and normalized TTN of *Sc*SLAC variants with phenolic substrates at pH 7.0 at 25 °C. The normalized TTNs were divided by the TTN of *Sc*SLAC M298. The data from the plots in **b**, **c** and **f** are provided as the mean and standard deviation of biological triplicates. Source data

Intrigued by the spectroelectrochemical results, we characterized the catalytic activity of each variant (Fig. 3). The common laccase substrate 2,2′-azino-bis(3-ethylbenzothiazoline-6-sulfonic acid) (ABTS; 680 mV versus NHE^73^) was used to benchmark the activity (Fig. 3a), and Michaelis–Menten kinetics were determined at pH 4 (Fig. 3b,c, Supplementary Fig. 7 and Supplementary Table 3). The *k*_cat_ of the two fungal-like *Sc*SLAC variants M298F and M298L were eightfold and fivefold reduced from wt *Sc*SLAC, respectively. Additionally, the mutations M298I and M298Q were similarly inactive (Supplementary Fig. 8 and Supplementary Table 3). By contrast, mutation of the axial ligand to M298**C5a** and M298**C6a** increased the *k*_cat_ by approximately twofold for each variant. Additionally, the *k*_cat_/*K*_M_ also increased for M298**C5a** and M298**C6a** by 1.6-fold and 1.8-fold, respectively. These results suggest that with low redox potential substrates, these hydrophobic ncAAs have a positive effect on the rate-limiting step of catalysis.

To evaluate catalysis with higher-redox-potential substrates, the activity of *Sc*SLAC variants was assessed against a panel of phenolic compounds with varying redox potentials^74^ (Fig. 3d–f and Supplementary Figs. 9–11). For each variant, we determined the TTNs after 24 h. Many of these substrates have higher redox potentials than the T1Cu centres, making substrate oxidation thermodynamically unfavourable (endergonic). However, substrate oxidation is coupled to reduction of molecular oxygen, an exergonic reaction (~820 mV at pH 7; ref. ^75^) and coupled to additional steps that are also reportedly exergonic, including binding of molecular oxygen, proton transfer and release of water^76^. Such coupling mechanisms are widely used in biological systems and enable thermodynamically unfavourable transformations to occur efficiently^77–79^. Thus, it is commonly observed that laccases can oxidize substrates with redox potentials greater than their *E*°′_T1Cu_ values (refs. ^39,80^).

Turnover analysis for the fungal-like variants—M298F and M298L—revealed that both variants exhibit lower TTNs for almost all substrates compared with wt *Sc*SLAC. For M298**C5a** and M298**C6a**, a general trend was observed where with increasing substrate redox potential, the TTN relative to wt *Sc*SLAC increased. With the highest potential substrates, M298**C5a** and M298**C6a** showed an improvement of TTN compared with the wt of up to fivefold and sixfold, respectively (Fig. 3f). Although a correlation between TTN and substrate redox potential is clear for both M298**C5a** and M298**C6a**, we observed some deviations that are suggestive of weak substrate-specific effects, indicating that substrate redox potential alone is an insufficient descriptor. Collectively, these results indicate that the ncAA-containing *Sc*SLAC variants can be used for the oxidation of redox-inert substrates and suggest the potential to oxidize molecules that are intractable with wt *Sc*SLAC.

### Structural changes in M298C6a are focused near the T1Cu centre

**Fig. 4:**
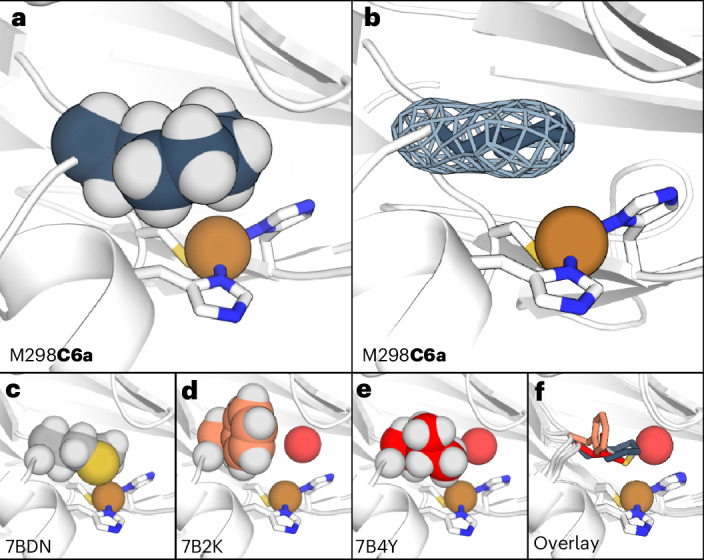
Structural comparison of *Sc*SLAC variants. **a**, The structure of *Sc*SLAC M298**C6a** was determined by X-ray crystallography (PDB no. 9HU7). **b**, The 2Fo-Fc electron density map (*σ* = 8, where Fo and Fc are the observed and calculated structure factors, respectively, and *σ* represents the root-mean-square electron density of the map) rendered in mesh for the **C6a** side chain shows the excess electron density for the **C6a** side chain when the side chain is left out of the model—indicating that the side-chain orientation is supported by the experimental data. **c**–**e**, Space-filling views for wt *Sc*SLAC M298 (**c**, PDB no. 7BDN), M298F (**d**, PDB no. 7B2K) and M298L (**e**, PDB no. 7B4Y). **f**, An overlay of the structures shows the relative orientations of the side chains and the potential steric clash between the ordered water molecules and the M298 and M298**C6a**. The copper ions are illustrated as orange spheres; the side chains of the axial amino acids are depicted as sticks; and the ordered water molecules near the T1Cu copper in M298F and M298L are each shown as a single red sphere for the oxygen atom. For all spheres, the relative scale is set to 70% of the van der Waals radii.

To understand the structure–function relationships that give rise to improvement with M298**C5a** and M298**C6a**, we turned to protein crystallography. We obtained crystallographic data of *Sc*SLAC M298**C6a** (Fig. 4a,b; PDB no. 9HU7; Extended Data Table 1). The resolution (3.51 Å) was not sufficient to distinguish between a chair, twist or boat conformation of the cyclohexyl ring. Thus, the chair conformation was selected for refinement because it is thermodynamically more stable^81^. The *Sc*SLAC M298**C6a** structure was compared with previously reported structures for fungal-like SLAC variants^42,55,62^ (Fig. 4c–f and Supplementary Fig. 6). Global comparison of the *Sc*SLAC M298**C6a** structure to the other *Sc*SLAC variants indicated that the overall structures are nearly identical (r.m.s.d. values, 0.218–0.234 Å). However, distinct features were observed at the T1Cu centre. In *Sc*SLAC M298**C6a**, the orientation of the **C6a** side chain partly envelops the copper ion similarly to that of the methionine side chain in wt *Sc*SLAC, but M298**C6a** lacks the S–Cu coordination. By contrast, canonical mutations of the axial methionine produced a large cavity within 5 Å of the T1Cu, enabled by either the smaller side chain or, in the case of planar aromatics, a flipping away of the aromatic ring^42,55,62^ (Fig. 4d,e and Supplementary Fig. 6). Within these cavities, a T1Cu-coordinating water molecule and one to two ordered water molecules within 5 Å of the T1Cu centre are observed^55,62^. It has been suggested that these ordered water molecules contribute to increased reorganization energy that negatively affects *k*_cat_ in these mutants. Comparatively, the bulk of the **C6a** side chain fills the space, such that a cavity cannot be identified in this variant. These results are consistent with those observed for the redox potential predictions, further supporting the differences in water accumulation between the ncAA variants and the fungal-like mutants. Additional computational analysis indicates that the 10 Å around the T1Cu centre of M298**C6a** is more fungal-like than other bacterial laccase mutants (Supplementary Fig. 13), with hydrophobicity being the primary distinguishing factor.

### Traditional mutagenesis from M298C6a further improves activity

**Fig. 5:**
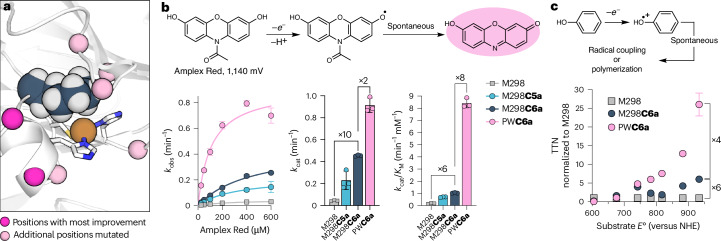
Traditional directed evolution applied to *Sc*SLAC M298**C6a**. **a**, Positions near the T1Cu site selected for site-saturation mutagenesis: F195, M298, I200, Y229, V290, S292 and M296. **b**, Michaelis–Menten kinetics of Amplex Red oxidation and its rapid spontaneous decay to resorufin at pH 7.0 and 25 °C (Supplementary Fig. 14 and Supplementary Table 4). The reaction scheme is shown above. **c**, Oxidation of phenolic substrates. The general reaction scheme for oxidation of phenolic substrates by laccases (top) and the TTNs with phenolic substrates at pH 7.0 and 25 °C. The TTNs are normalized to the TTN of wt *Sc*SLAC. Absolute values are shown in Supplementary Fig. 10. The data from the plots in **b** and **c** are provided as the mean and standard deviation of biological triplicates. Source data

To test if traditional enzyme engineering could further improve this system, we performed a directed evolution campaign (Fig. 5). Using the high-redox-potential substrate Amplex Red^82^ (1.14 V versus NHE^83^), we developed a lysate-based screening assay. Amplex Red can undergo one-electron oxidation with subsequent rapid and spontaneous decay to resorufin (Fig. 5b), which can be detected via fluorescence spectroscopy in a 96-well plate format. We first compared Michaelis–Menten parameters with Amplex Red for wt *Sc*SLAC, M298**C5a** and M298**C6a**. Gratifyingly, the *k*_cat_ values for M298**C5a** and M298**C6a** were fivefold and tenfold higher than the *k*_cat_ of wt *Sc*SLAC, respectively, showing markedly more improvement with this high-redox-potential substrate than that observed for ABTS (Fig. 3b). Based on performance and the commercial availability of **C6a**, we selected M298**C6a** as the starting point for further engineering.

**Extended Data Fig. 3:**
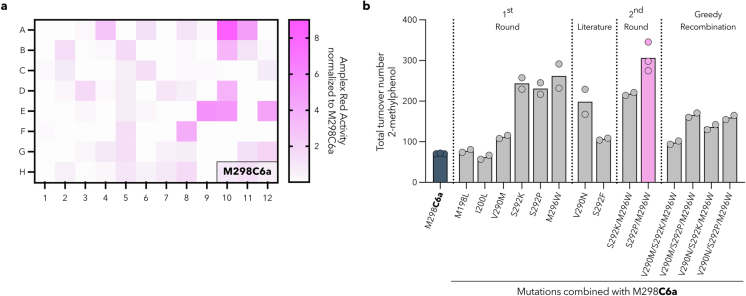
Traditional directed evolution from M298C6a. **a**) A representative result of the lysate screening assay from site-directed mutagenesis for further engineering of M298**C6a**. As a positive control, M298**C6a** was used as a standard in wells H10-12, and activity was normalized to M298**C6a**. **b**) Selected variants were purified and the total turnover number with 2-methylphenol was measured. M298**C6a** and PW**C6a** were prepared in biological triplicates, the other samples were prepared in preparative duplicates. Mutants V290N and S292F were included based on previous literature reports^54^. The independent data points are shown, and the bars represent the mean values. Source data

**Extended Data Fig. 4:**
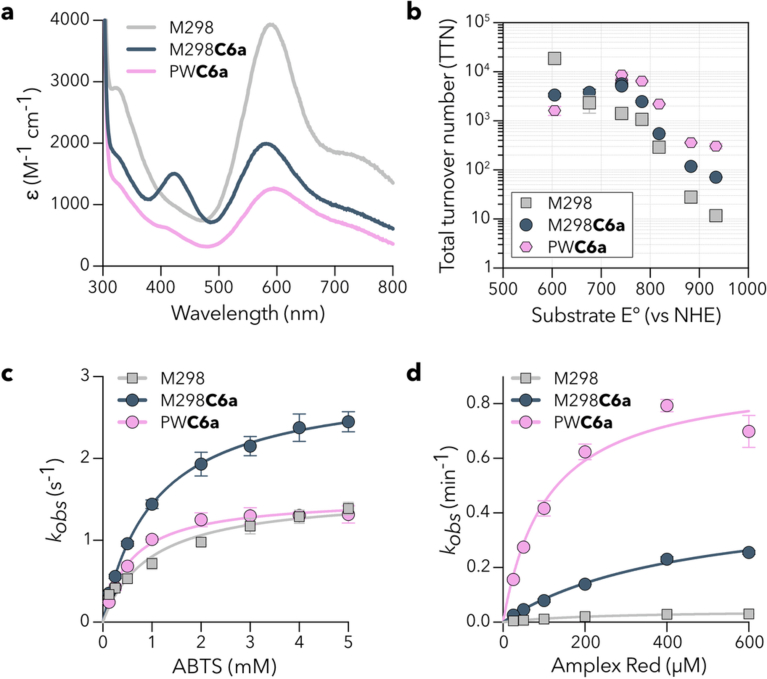
Comparison along evolutionary trajectory: UV-vis spectra and catalytic activity. The UV-vis spectra (**a**), activities for phenolic oxidation (**b**), ABTS oxidation (**c**), and Amplex Red oxidation (**d**) for M298, M298**C6a**, and PW**C6a**. The data shown in panels b-d are provided as the mean and standard deviation of biological triplicates. Source data

Seven positions within M298**C6a** were selected for iterative single site-saturation mutagenesis over three rounds (Fig. 5a and Extended Data Fig. 3). Identification of the best mutant was based on both the rapid Amplex Red assay and a secondary LC-MS screen based on the oxidation of 2-methylphenol. From three rounds of screening, the most-improved variant was the triple mutant S292P/M296W/M298**C6a** (PW**C6a**). The PW**C6a** mutant was characterized similarly to M298**C6a** (Extended Data Fig. 4 and Supplementary Figs. 3–5). Strikingly, the combination of semi-rational ncAA mutagenesis and traditional directed evolution resulted in an overall increase in *k*_cat_ and catalytic efficiency of 20-fold and 47-fold, respectively, for PW**C6a** compared with wt *Sc*SLAC (Supplementary Table 5).

To understand the differences between our results from semi-rational engineering with ncAAs and from traditional directed evolution, we examined the changes in catalysis over the evolutionary trajectory. The introduction of M298**C6a** induced a tenfold improvement in *k*_cat_ and a sixfold improvement in catalytic efficiency with Amplex Red. Subsequent introduction of two additional mutations (PW**C6a**) improved the *k*_cat_ only twofold but resulted in an eightfold enhancement of the catalytic efficiency, owing to a decrease in *K*_M_ (Supplementary Tables 4 and 5). To parse the kinetic effects of M298**C6a** and the S292P/M296W mutations, we characterized the kinetics of Amplex Red oxidation with *Sc*SLAC S292P/M296W (Supplementary Table 4). The changes in *k*_cat_ were nearly additive towards PW**C6a** (normalized epistasis^84^, 0.10), indicating that the effects on the rate-limiting step are independent and similar in magnitude (tenfold and ninefold improvement for M298**C6a** and S292P/M296W, respectively). By contrast, the changes in *k*_cat_/*K*_M_ are non-additive, with PW**C6a** showing improvements substantially higher than the sum of the improvements for *Sc*SLAC S292P/M296W and M298**C6a** (normalized epistasis, 4.6). This high level of epistasis indicates that these mutations are acting cooperatively to improve the catalytic efficiency. Thus, in this case, rational introduction of a single ncAA provided similar effects on *k*_cat_—the targeted parameter—as several rounds of traditional directed evolution. However, combining the two methodologies significantly enhanced the overall catalytic performance.

Although we tried to obtain a crystal structure of PW**C6a**, no diffraction-quality crystals could be obtained. In the crystal structure of M298**C6a**, both S292 and M296 are on a short helix that links the T1Cu-coordinating C288 to M298**C6a**. This helix also contains H293, the surface-exposed histidine residue that coordinates the T1Cu centre and can interact with the substrate. In the crystal structure of M298**C6a**, both S292 and M296 face the surface of the protein, with S292 packing against H293 and M296 covering M298**C6a**.

Lastly, we compared the catalytic efficiencies of the different variants with the highly active fungal laccase from *Trametes versicolor*. From the starting wt *Sc*SLAC, which has a 244-fold lower catalytic efficiency, we were able to enhance the catalytic efficiency 50-fold to only fivefold lower than the *T. versicolor* fungal laccase (Supplementary Table 4). These results demonstrate the efficacy of this combined method to achieve substantial catalytic improvements approaching the efficiency of a fungal laccase. Collectively, these results highlight the value of this dual-mutation strategy, leveraging both semi-rational and classical approaches. Nonetheless, we note that semi-rational engineering with ncAAs was only practical through knowledge of the rate-limiting kinetics.

## Discussion

Mutagenesis with ncAAs has shown continued promise for enzymatic characterization and enzyme engineering. Herein we enabled the genetic incorporation of three extremely hydrophobic ncAAs—**C5a**, **C6a** and **C7a**—and demonstrated their potential for hydrophobic tuning using semi-rational enzyme engineering with the bacterial laccase *Sc*SLAC. Most ncAA-based enzyme engineering focuses on improved non-native activity, often through improving substrate recognition, enhancing enantioselectivity or installing an abiotic catalytic entity. Here we demonstrate that ncAA-based enzyme engineering can be used to enhance near-native catalysis and directly accelerate the rate-limiting chemical step(s) through semi-rational design using fundamental chemical principles. This approach enabled improvements up to tenfold in the rate-limiting step of catalysis with concomitant improvements in the *E*°′_T1Cu_, catalytic efficiency and TTN. Functional and structural characterization of M298**C6a** as well as comparisons with previously studied bacterial and fungal laccases suggest that the increased hydrophobicity can largely account for the improved redox potential. However, we propose that the hydrophobic ncAA mutations could exhibit improved activity through a multi-factorial stabilization Cu^1+^ state: (1) removing electron density from the T1Cu site through elimination of the methionine ligand, (2) increasing the hydrophobicity around the T1Cu site and (3) providing steric bulk, limiting water accumulation around the T1Cu and also potentially limiting the reorganization energy upon reduction from Cu^2+^ to Cu^1+^. In addition to the ncAA-based engineering, further catalytic improvements were derived through a short, traditional directed evolution campaign from the best-performing ncAA mutant. Our results highlight both the synergistic potential and the differences in these two approaches. We anticipate that further improvements could be derived through computationally guided engineering^85,86^ and more extensive combinatorial directed evolution approaches^87^. Additional mutants with improved activity could enable unprecedented applications for these enzymes.

Moreover, beyond the work presented herein, we expect that these genetic code expansion tools will be valuable to explore and fine-tune the hydrophobicity of proteins in catalysis^5^ and binding interactions^88^. Owing to the use of **C6a** for modulating peptide properties, we anticipate that the ability to genetically incorporate this ncAA could have applications in peptide chemistry as well^28,89,90^. Additionally, because PylRS/tRNA^Pyl^ pairs can be typically shuttled between *E. coli* and eukaryotic cells^91^, the developed tools could be applied to study and tune the effects of hydrophobicity of a wide array of proteins in various hosts. Our findings suggest that the continued development of robust translation systems for ncAA incorporation will considerably enhance the viability and value of ncAA-based enzyme engineering.

## Methods

### General instrumentation and materials

All purchased chemicals were used as delivered and not further purified. UV–vis data were measured either on a Multiskan Sky (Thermo Scientific) using the SkanIt RE v.6.0.1 software or a Cary 60 UV–vis (Agilent) using the Cary WINUV Scan Application v.5.0.0.999, except for kinetics measurements, which were measured on a Tecan Infinite M Nano+ using the i-control v.2.0 software. The ^1^H NMR spectra were recorded in CDCl_3_ or D_2_O on a Bruker AV-400 (400 MHz) using Topspin v.4.1.3, with chemical shift *d* in parts per million relative to solvent signals (*d* = 7.26 ppm for CDCl_3_, 4.79 ppm for D_2_O) and coupling constants *J* given in hertz. The ^13^C NMR spectra were recorded in D_2_O on a Bruker AV-400 (400 MHz) using Topspin v.4.1.3. NMR data were analysed with MestreNova v.14.1.2. Automated flash chromatography was conducted on a Biotage Isolera instrument. All sequencing was conducted by Microsynth. Primers were ordered from Microsynth or Integrated DNA Technologies (IDT). Gene fragments were ordered from Twist Bioscience. Plasmids were assembled via Gibson assembly.

### Mass spectrometry methods

During aaRS/tRNA screening, initial high-throughput LC-MS of sfGFP was conducted using an Agilent single-quadrupole mass spectrometer with electrospray ionization (ESI), coupled to a 1290 Infinity II LC system using the OpenLab CDS v.2.7 software.

Verification of all proteins discussed in the text was additionally carried out by high-resolution LC-MS carried out at the Functional Genomics Centre Zürich (FGCZ). Briefly, samples were resolved on an ACQUITY ultra-performance LC (UPLC) BioResolve RP mAb column (2.7 µm, 2.1 mm × 150 mm, 450 Å). The analysis was performed on a Synapt G2-Si mass spectrometer directly coupled to the UPLC station. Mass spectra were acquired in the positive-ion mode by scanning the *m*/*z* range from 400 to 5,000 Da with a scan duration of 1 s and an interscan delay of 0.1 s. The spray voltage was set to 3 kV, the cone voltage to 50 V and the source temperature to 100 °C. The data were recorded with the MassLynx v.4.2 software. The recorded *m*/*z* data of single peaks were deconvoluted into mass spectra by applying the maximum entropy algorithm MaxEnt1 (MaxLynx) with a resolution of the output mass 0.5 Da per channel and Uniform Gaussian Damage Model at the half height of 0.7 Da.

LC-MS/MS was carried out by the FGCZ. Purified protein (~1 mg ml^−1^) was digested in solution by mixing 5 µl sample with 40 µl digestion buffer (10 mM Tris, 2 mM CaCl_2_, pH 8.2). Protein was reduced and alkylated by 0.9 µl of 100 mM tris(2-carboxyethyl)phosphine plus 1.4 µl of 100 mM 2-chloroacetamide. Some 2 µl trypsin (100 ng µl^−1^ in 10 mM HCl) was added, and microwave assisted digestion was carried out at 60 °C for 30 min. The samples were dried and dissolved in 20 µl double-distilled H_2_O plus 0.1% formic acid. Samples were analysed on an M class UPLC system coupled to a Q Exactive mass spectrometer (Thermo). Data were searched against the sfGFP sequence by Byonic v.5.2 (Protein Metrics).

Small-molecule LC-MS analysis and UPLC analysis were both conducted with an Agilent 1290 Infinity II LC system coupled to a single-quadrupole mass spectrometer (ESI).

### aaRS library construction

A set of libraries of PylRS from *M. mazei* (*Mm*PylRS) was prepared by randomizing between three and five residues of the substrate binding site using site-saturation mutagenesis (Fig. 1b and Supplementary Table 6). Additionally, a library of *Mm*PylRS containing the fixed I405R mutation was prepared^92^. Libraries were created using modified Golden Gate cloning for mutagenesis at the positions listed in Supplementary Table 6 with the primers listed in Supplementary Data 1. A pSL plasmid encoding the corresponding synthetase (GenBank no. PX848775) was amplified using inverse polymerase chain reaction (PCR) with primers carrying a BsaI restriction site and an additional 6 bp overhang at the 5′ end. PCR products were purified using an NEB Monarch PCR clean-up kit. The purified PCR products were digested with DpnI and BsaI in ×1 rCutSmart. Digests were carried out at 37 °C overnight. Digested products were purified using an NEB Monarch PCR clean-up kit. DNA ligation reactions contained T4-ligase and ×1 T4-ligase buffer. Ligation was carried out at 16 °C overnight. Ligation products were purified using an NEB Monarch PCR clean-up kit. Electrocompetent NEB10β *E. coli* (100 µl) were transformed with ligation product by electroporation in a cuvette with a 2 mm gap on an Eppendorf Eporator with 250 Ω and 2,500 V and with pulse times of ~5 ms. The cells were recovered in super optimal broth with catabolite repression (SOC) media for 1 h at 37 °C with shaking at 220 rpm. The number of transformants was estimated by dilution series plating of lysogeny broth (LB) agar with 50 μg ml^−1^ kanamycin. The recovered cells were transferred to 10 ml LB media with 50 μg ml^−1^ kanamycin and grown for 5 h. The cells were harvested by centrifugation, and the DNA was isolated using an NEB Monarch Plasmid Miniprep Kit. The library quality was confirmed by Sanger sequencing of two to three individual clones.

### aaRS library selections

For positive selections, electrocompetent NEB10β cells (100 µl) carrying a pDPS2-*Mm*tRNA^Pyl^_CUA_ plasmid (GenBank no. PX848774) were transformed with 250 ng of a given library by electroporation in a cuvette with a 2 mm gap on an Eppendorf Eporator at 250 Ω and 2,500 V with pulse times of ~5 ms. The cells were recovered in SOC media for 1 h at 37 °C with shaking and transferred to 10 ml LB media with 50 μg ml^−1^ kanamycin, 10 μg ml^−1^ tetracycline and 6 mM **C6a**. The cells were grown for 1–2 h, harvested by centrifugation, resuspended in 250 μl LB media and plated on LB agar with 50 μg ml^−1^ kanamycin, 10 μg ml^−1^ tetracycline, 100 μg ml^−1^ chloramphenicol, 0.4% arabinose and 6 mM **C6a**. Plates were incubated for 24 h at 37 °C. If additional selection rounds were carried out, cells were collected by washing the plate with 10 ml LB media and harvesting the cells by centrifugation. Plasmid DNA was isolated using an NEB Monarch Plasmid Miniprep Kit. DNA was digested with AgeI for 18 h at 37 °C to remove the selection plasmid and purified using an NEB Monarch PCR clean-up kit. If additional selection rounds were not carried out, we proceeded as though the step was the final positive selection round described further below.

For negative selections, electrocompetent NEB10β cells (100 µl) carrying a pBARN-*Mm*tRNA^Pyl^_CUA_ plasmid (GenBank no. PX848776) were transformed with 50 ng of library DNA by electroporation in a cuvette with a 2 mm gap on an Eppendorf Eporator at 250 Ω and 2,500 V with pulse times of ~5 ms. The cells were recovered in SOC media for 1 h at 37 °C with shaking and transferred to 10 ml LB media with 50 μg ml^−1^ kanamycin and 35 μg ml^−1^ chloramphenicol. The cells were grown in the presence of the antibiotics for 1–2 h. The cells were harvested by centrifugation, resuspended in 250 μl LB media and plated on LB agar with 0.4% arabinose, 50 μg ml^−1^ kanamycin and 35 μg ml^−1^ chloramphenicol. Plates were incubated for 24 h at 37 °C. Cells were collected by washing the plate with 10 ml LB media, cells were harvested by centrifugation and plasmid DNA was purified using an NEB Monarch Plasmid Miniprep Kit. DNA was digested with AgeI in ×1 rCutsmart. Digests were carried out at 37 °C overnight to the remove selection plasmid and purified using an NEB Monarch PCR clean-up kit.

For screening after the final round of positive selection, individual colonies were used to inoculate 96-well plates with 250 μl 2xYT media with 50 μg ml^−1^ kanamycin and 10 μg ml^−1^ tetracycline. Cultures were grown for 24 h at 37 °C with shaking at 400 rpm. Subsequently, 25 μl of each culture was used to inoculate 96-well plates with 250 μl 2xYT media with 50 μg ml^−1^ kanamycin, 10 μg ml^−1^ tetracycline, 0.4% arabinose and 0 or 6 mM **C6a**. Cultures were grown for 24 h at 37 °C with shaking at 400 rpm. Some 100 μl of each culture was transferred to a 96-well clear well plate. Fluorescence (excitation at 480 nm and emission at 510 nm) and optical density at 600 nm (OD_600_) were measured. DNA from cultures with high fluorescence/OD_600_ ratios was isolated using an NEB Monarch Plasmid Miniprep Kit and analysed by Sanger sequencing to identify mutations in the PylRS gene.

### sfGFP amber suppression assay

NEB10β *E. coli* were co-transformed with pBAD-sfGFP150_TAG_ (GenBank no. PX848777, modified from Addgene no. 85483)^93^ and the corresponding aaRS/tRNA expression plasmid (GenBank nos. PX848771 and PX848772) by heat shock (42 °C, 30 s), recovered in SOC for 1 h at 37 °C and plated on LB agar with 50 μg ml^−^ kanamycin and 10 μg ml^−1^ tetracycline. Plates were incubated for 24 h at 37 °C. Individual colonies were used to inoculate 250 μl autoinduction media with 100 μg ml^−1^ kanamycin, 20 μg ml^−1^ tetracycline and the ncAA. The cultures were grown for 24 h at 37 °C with shaking at 400 rpm. For each ncAA, the concentrations were as follows: 6 mM **C6a** and 12 mM dl-ncAA for **C5a** and **C7a**. The wt sfGFP was analogously expressed from pBAD-sfGFP by omission of 10 μg ml^−1^ tetracycline. Some 100 μl of each culture was transferred to a 96-well clear well plate. Fluorescence (excitation at 480 nm and emission at 510 nm) and absorbance at 600 nm were measured.

### Analysis of ncAA incorporation in sfGFP

NEB10β *E. coli* were co-transformed with pBAD-sfGFP150_TAG_ and the corresponding aaRS/tRNA expression plasmid by heat shock (42 °C, 30 s), recovered in SOC for 1 h at 37 °C and plated on LB agar with 50 μg ml^−1^ kanamycin and 10 μg ml^−1^ tetracycline. Plates were incubated for 24 h at 37 °C. Individual colonies were used to inoculate 5 ml autoinduction media with 100 μg ml^−1^ kanamycin, 20 μg ml^−1^ tetracycline and either 12 mM dl-**C5a**, 6 mM **C6a** or 12 mM dl-**C7a**. The cultures were grown for 24 h at 37 °C with shaking at 240 rpm. The cells were harvested by centrifugation for 10 min at 4 °C and 4,200*g*; the supernatant was decanted; and the cell pellets were stored at –80 °C. To isolate the sfGFP, the cells were thawed at room temperature and resuspended in lysis buffer (500 μl, 20 mM Tris, 300 mM NaCl, pH 7.2 at 4 °C, 0.2% *n*-octyl β-d-thioglucopyranoside, 4 mg ml^−1^ lysozyme). The lysis was conducted at 22 °C for 4 h. The sfGFP was isolated by purification with Ni–nitrilotriacetic acid (Ni–NTA) resin (HisPur from Thermo Scientific) according to the manufacturer’s instructions. The purified sfGFP was analysed by LC-MS as indicated in the mass spectrometry section.

### *Sc*SLAC production

The N-terminal signalling peptide (amino acids 2–30) of *Sc*SLAC is highly prone to proteolysis^94^. In laccases, this region is not necessary for catalysis. In many laccase studies—including *Sc*SLAC studies—the N-terminal region is truncated^38,87,94,95^. Thus, to reduce complexity and ensure that LC-MS could be used to identify ncAA incorporation, we designed a *Sc*SLAC construct without the N-terminal signalling peptide and with a C-terminal His6 tag for purification. Both constructs yielded similar catalytic parameters with ABTS, which were similar to previous literature reports with similar conditions^54^. Thus we continued with the N-terminally truncated *Sc*SLAC–His6 on a pET28 plasmid (GenBank no. PX848773). The sequence is provided in the DNA and protein sequence section. Primers used for the cloning of *Sc*SLAC variants are provided in Supplementary Data 1.

To produce *Sc*SLAC, the corresponding pET28-SLAC-M298X (where X is M, L, F, Q or I) plasmid was transformed into B-95ΔAΔfabR cells (Addgene no. 197934)^59^. Precultures were grown at 37 °C overnight in LB with 50 μg ml^−1^ kanamycin from single colonies or glycerol stocks. The cells were diluted 100-fold into 25–50 ml autoinduction media with 50 μg ml^−1^ kanamycin in a baffled flask and shaken at 37 °C for 3 h. CuCl_2_ was added to a final concentration of 2 mM, and the cells were shaken (160 rpm) at 25 °C for 28 h. The cells were collected by centrifugation (10 min, 4 °C, 4,200*g*), the supernatant was decanted and the cells were frozen at –80 °C.

### *Sc*SLAC production with ncAAs

B-95ΔAΔfabR cells (Addgene no. 197934)^59^ were transformed with pGS1T-PylRS^C6a^–PylT^m15^_CUA_ and the desired pET28-SLAC-M298_TAG_ plasmid. Precultures were grown at 37 °C overnight in LB with 50 μg ml^−1^ kanamycin and 10 μg ml^−1^ tetracycline from single colonies or glycerol stocks. The cells were diluted 100-fold into 50–100 ml autoinduction media with 50 μg ml^−1^ kanamycin, 10 μg ml^−1^ tetracycline and either 20 mM dl-**C5a**, 8 mM **C6a** or 12 mM dl-**C7a** in a baffled flask and shaken at 37 °C for 8–14 h. CuCl_2_ was added to a final concentration of 2 mM, and the cells were shaken (160 rpm) at 25 °C for 24–28 h. The cells were collected by centrifugation (10 min, 4 °C, 4,200*g*), the supernatant was decanted and the cells were frozen at –80 °C.

### *Sc*SLAC purification

Cells were thawed on ice and lysed with lysis buffer (20 ml g^−1^ cell pellet, 20 mM Tris, 500 mM NaCl, pH 8.0 at 4 °C, 0.25% *n*-octyl β-d-thioglucopyranoside, 2 mg ml^−1^ lysozyme, 0.25 µl ml^−1^ DNase, 2 mM MgCl_2_) at 4 °C overnight, turning end over end. CuCl_2_ was added to a final concentration of 2 mM, and the lysate was turned end over end at 4 °C for 3 h. The lysate was pelleted by centrifugation (18,000*g*, 4 °C, 1 h), and the supernatant was decanted. Ni–NTA beads (1 ml g^−1^ cell pellet) were added to the supernatant, and the suspension was turned end over end at 4 °C for 30–45 min. The Ni–NTA beads were collected by centrifugation (100*g*, 4 °C, 5 min), the supernatant was decanted and the beads were loaded onto a gravity flow column. The columns were washed four times with 4 ml of washing buffer (20 mM HEPES, 300 mM NaCl, 20 mM imidazole, pH 7.0), and the protein was collected with elution buffer (20 mM HEPES, 300 mM NaCl, 500 mM imidazole, pH 7.0). Fractions with protein were combined, and the protein was buffer exchanged to storage buffer (20 mM HEPES, 300 mM NaCl, pH 7.0) using a Zeba Spin Desalting Column (2 ml or 5 ml, 7 kDa molecular weight cut-off). Fractions with protein were pooled, flash frozen in liquid nitrogen and stored at –80 °C.

### Protein concentration determination

Protein concentrations of expressed proteins were measured by absorbance at 280 nm on a NanoDrop 2000c spectrophotometer from Thermo Scientific using the NanoDrop 2000/2000c operation software v.1.6.198. Protein concentration of the fungal laccase was determined by Bradford assay.

### Crystallization and structure determination

*Sc*SLAC M298**C6a** was concentrated to 18–20 mg ml^−1^ using Amicon Ultra-4 centrifugal filter tubes (10 kDa molecular weight cut-off, Merck Millipore) in storage buffer (20 mM HEPES, 300 mM NaCl, pH 7.0). Crystals of *Sc*SLAC M298**C6a** were obtained at 20 °C within four weeks by sitting-drop vapour diffusion with *Sc*SLAC M298**C6a** (150 nl) and a precipitant of 18.2% Jeffamine M-600 and 17.5% dimethyl sulfoxide (DMSO; 100 nl precipitant). The crystals were mounted onto MicroMesh (400/10 µm, MiTiGen) and flash-cooled in liquid nitrogen after the addition of 25% glycerol as cryoprotectant.

Data were collected at the European Synchrotron Radiation Facility (ESRF) MASSIF-3/ID30-A3 beamline at 0.9677 Å. Multiple datasets of one crystal were collected due to weak diffraction. The data were processed using the Autoprocess pipeline^96–101^. The data could be merged and scaled to 3.51 Å and were solved by molecular replacement using the AlphaFold3 (ref. ^102^) model of the protein. The crystal contained one molecule per asymmetric unit with a high solvent content of >85% that may have contributed to the weak diffraction but may have aided bulk solvent correction during crystallographic refinement. The initial model was refined and rebuilt iteratively in Coot^103^ and phenix.refine^104^. *Sc*SLAC M298**C6a** could be refined to an *R*_work_/*R*_free_ (crystallographic *R* factors measuring the fit of the structural model to the diffraction data) of 0.2048:0.2185 and a Molprobity score of 0.88. The map was of exceptional quality (Supplementary Fig. 12). The PDB model has been deposited in the PDB as no. 9HU7. In the PDB deposition, **C6a** was termed ALC based on previous PDB entries. Crystallographic details are provided in Extended Data Table 1.

### Michaelis–Menten kinetics with ABTS

The reactions were carried out in 20 mM Britton–Robinson buffer (20 mM acetic acid, 20 mM phosphoric acid, 20 mM boric acid, pH adjusted with aqueous NaOH) at pH 4.0. To prepare the reactions, ×2 solutions of reaction components were prepared as follows: ×2 Britton–Robinson buffer, 2 mM CuCl_2_ and varying ABTS concentrations (0.2, 0.5, 1, 2, 4, 6, 8 or 10 mM). Separately, the desired enzyme was diluted to 400 nM in water for wt, **C5a** and **C6a** and to 2,000 nM for M298L and M298F. To initiate the reaction, 50 µl of the enzyme solution was added to 50 µl of the ×2 reaction component solution. As a control, samples without enzyme were prepared with 50 µl of water instead of the 50 µl of dilute enzyme. Immediately after mixing by pipetting up and down, the absorption change at 420 nm (*ε*_420_ = 36,000 M^−1^ cm^−1^) was measured every 9–10 s. The reaction was carried out at 25 °C. For data processing, for each ABTS concentration, the time trace corresponding to the negative control (without enzyme) was subtracted from each time trace containing protein. The kinetic parameters were determined by Michaelis–Menten analysis.

### Redox potential determination

Redox potentials were determined by redox titration using K_3_[Fe(CN)_6_] as a redox dye and sodium dithionite as reductant. The titrations were performed in a glove box under nitrogen atmosphere. The following components were mixed together in a total volume of 250 µl: 110–285 µM protein, 500 µM K_3_[Fe(CN)_6_] and 4 µM methyl viologen in 20 mM HEPES and 300 mM NaCl, at pH 7.0. The absorbance spectra were measured from 300–900 nm after every addition of 0.5–2 µl sodium dithionite (5 mM) and an equilibration time of 20 min. A baseline correction was made based on absorbance at 900 nm. The amount of oxidized protein and dye was determined by spectral deconvolution by fitting linear combinations of the spectra of oxidized protein and dye from 380 to 650 nm. The Python code for this deconvolution is available for use (as described in the ‘Code availability’ statement)^105^. The reduced species did not absorb within the wavelength range of interest. The percentage of oxidized and reduced dye was used to determine the redox potential of the system using the Nernst equation as follows:

$${E}_{\mathrm{cell}}={E}^{\circ\prime}_{\mathrm{cell}}-\frac{{RT}}{{zF}}\mathrm{ln}\left(\frac{{X}_{\mathrm{red}}}{{X}_{\mathrm{ox}}}\right)$$

where *E*_cell_ is the potential of the cell, ${E}^{\circ\prime}_{\mathrm{cell}}$ is the standard potential of the cell, *R* is the ideal gas constant, *T* is the temperature, *z* is the number of electrons transferred, *F* is Faraday’s constant, *X*_red_ is the concentration of the reduced species and *X*_ox_ is the concentration of the oxidized species. At equilibrium, the two half reactions can be described as follows:

$${E}^{\circ\prime}_{\mathrm{dye}}-\frac{{RT}}{{zF}}\times {\mathrm{ln}}\left(\frac{{\mathrm{Dye}}_{\mathrm{red}}}{{\mathrm{Dye}}_{\mathrm{ox}}}\right)={E}^{\circ\prime}_{{\rm{T}}1{\mathrm{Cu}}}-\frac{{RT}}{{zF}}\times {\mathrm{ln}}\left(\frac{{{\rm{T}}1{\mathrm{Cu}}}_{\mathrm{red}}}{{{\rm{T}}1{\mathrm{Cu}}}_{\mathrm{ox}}}\right)$$

where ${E}^{\circ\prime}_{\mathrm{dye}}$ is the standard redox potential of the dye, Dye_red_ is the concentration of reduced dye, Dye_ox_ is the concentration of the oxidized dye, ${E}^{\circ\prime}_{{\rm{T}}1{\mathrm{Cu}}}$ is the redox potential of the T1Cu site, T1Cu_red_ is the concentration of reduced T1Cu site and T1Cu_ox_ is the concentration of the oxidized T1Cu site. A redox potential of 436 mV versus NHE for K_3_[Fe(CN)_6_] was used^54^, and the conditions for *RT*/*zF* were *R* = 8.314 J K^−1^ mol^−1^, *T* = 293 K, *F* = 96,485 C mol^−1^ and *z* = 1. Varying *z* did not result in better-quality fits.

$$0.436\,{\rm{V}}-0.02525\times {\mathrm{ln}}\left(\frac{{\mathrm{Dye}}_{\mathrm{red}}}{{\mathrm{Dye}}_{\mathrm{ox}}}\right)={E}^{\circ\prime}_{{\rm{T}}1{\mathrm{Cu}}}-0.02525\times {\mathrm{ln}}\left(\frac{{{\rm{T}}1{\mathrm{Cu}}}_{\mathrm{red}}}{{{\rm{T}}1{\mathrm{Cu}}}_{\mathrm{ox}}}\right)$$

At each titration point, the deconvolution data provide the concentration of the reduced and oxidized dye and the concentration of the reduced and oxidized T1Cu centre. With this information, we can treat the equation as a linear function where the left side of the equation is *y*, ${E}^{\circ\prime}_{{\rm{T}}1{\mathrm{Cu}}}$ is the *y* intercept (*b*), –*RT*/*zF* is the slope (*m*) and ln(T1Cu_red_/T1Cu_ox_) is *x*. The predicted redox potentials based on log*P* were determined by a linear model: ${\mathrm{predicted}}\,{E}^{\circ\prime}_{{\rm{T}}1{\mathrm{Cu}}\_{\mathrm{variant}}}={\mathrm{slope}}\left({\mathrm{clog}} {P}_{\mathrm{variant}}-{\mathrm{clog}} {P}_{\mathrm{wt}}\right)+{E}^{\circ\prime}_{{\rm{T}}1{\mathrm{Cu}}\_{\mathrm{wt}}}$, where the clog*P*_variant_ and clog*P*_wt_ are the calculated log*P* of the mutated amino acid side chain and the methionine side chain, respectively, and the lower and upper limits are represented with a slope of 78 and 100, respectively, as reported previously^3^.

### EPR data collection and processing

For each SLAC variant, samples of approximately 300 µM enzyme were prepared in 20% glycerol and 80% 20 mM HEPES and 300 mM NaCl, pH 7.0. The X-band (9.5 GHz) continuous-wave (cw) EPR spectra were acquired on an Elexsys E500 EPR spectrometer (Bruker Biospin) equipped with an SHQ resonator (Bruker) and an ESR900 helium flow cryostat (Oxford Instruments) to maintain a stable temperature (40 K). The data were recorded using a magnetic-field modulation of 100 kHz with an amplitude of 0.2 mT and a lock-in conversion time of 81 ms at a non-saturating microwave power of 0.5 mW. All spectra were corrected by linear scaling to the same effective microwave frequency of 9.5 GHz, and the magnetic field was calibrated using commercial 2,2-diphenyl-1-picrylhydrazyl (DPPH, Merck). The EPR data were baseline corrected, and the baseline-corrected data were simulated in MATLAB 2017a with EasySpin (v.6.0.8)^106^. Because the T1Cu and T2Cu species overlap, Q-band continuous-wave EPR spectra of wt *Sc*SLAC and M298**C6a** were obtained to aid with fitting. The Q-band (34.5 GHz) EPR spectra were acquired on an Elexsys E580 EPR spectrometer (Bruker) equipped with a cryogen-free variable-temperature EPR cryostat (Cryogenic) using a home-built dedicated continuous-wave EPR resonator with 3 mm sample access^107^. For the four spectra of wt ScSLAC and M298**C6a**, a global fit of the X-band and Q-band data with shared parameters for the T2Cu site were obtained. These T2Cu parameters were used as starting points for the fits of the X-band data for M298L, M298F, M298**C5a** and PW**C6a**. Although hyperfine coupling from the coordinating nitrogen atoms could be observed in several X-band spectra, a robust fit was not possible with the added ^14^N parameters. Additionally, very broad features around 360 and 1,150 mT were observed; however, they could not be reliably fit. The data, simulated traces and simulated parameters are illustrated in Extended Data Fig. 2 and Supplementary Table 2.

### Total turnover number measurements

The reactions were carried out in 20 mM Britton–Robinson buffer (20 mM acetic acid, 20 mM phosphoric acid, 20 mM boric acid, pH adjusted with aqueous NaOH) at pH 7.0. The following reaction components were mixed together in a reaction volume of 100 µl: 970 µM phenolic substrate, 970 µM CuCl_2_ and 11.1–1,388 nM enzyme in 20 mM Britton–Robinson buffer. (For substrates with higher redox potentials, the enzyme concentrations were increased to ensure that the product concentration was well within the detection limit of our assay.) Reactions omitting enzyme were prepared as negative controls. The reaction was carried out at 25 °C for 24 h while shaking at 500 rpm in an Accutherm microtube shaking incubator. The reaction was quenched by the addition of 10 µl aqueous HCl (2 M). The reaction mixture was clarified by centrifugation (5 min, 14,000*g*), and 50 µl supernatant was diluted in 100 µl water. The mixture was analysed by UPLC. The UV–vis peak (220 nm) corresponding to the desired phenolic substrate was integrated, and the remaining, unconverted substrate concentration was determined by comparison to a standard calibration curve. The molar conversion was then calculated based on the concentration of substrate in the negative control minus the concentration of unconverted substrate. The final enzymatic conversion was then divided by enzyme concentration to determine the TTN.

### *Sc*SLAC library preparation

Libraries were created using modified Golden Gate cloning. A pET28 plasmid encoding the corresponding *Sc*SLAC template was amplified using inverse PCR with primers (Supplementary Data 1) carrying a BsaI restriction site and an additional 6 bp overhang at the 5′ end. PCR products were purified using an NEB Monarch PCR clean-up kit. The purified PCR products were digested with DpnI and BsaI in ×1 rCutSmart. Digests were carried out at 37 °C for 6 h. Digested products were purified using an NEB Monarch PCR clean-up kit. DNA ligation reactions contained T4-ligase and ×1 T4-ligase buffer. Ligation was carried out at 16 °C overnight. Ligation products were purified using an NEB Monarch PCR clean-up kit. Chemically competent B-95ΔAΔfabR cells (Addgene no. 197934)^59^ carrying pGS1T-PylRS^C6a^–PylT^m15^_CUA_ were transformed with ligation mixture by heat shock (42 °C, 30 s), recovered in SOC at 37 °C for 1 h and plated on LB agar with 50 μg ml^−1^ kanamycin and 10 μg ml^−1^ tetracycline.

### *Sc*SLAC lysate-based screening

Individual clones from *Sc*SLAC libraries were picked into 96-well deep well plates with 250 μl LB media with 50 μg ml^−1^ kanamycin and 10 μg ml^−1^ tetracycline and grown at 37 °C overnight. For single site-saturation mutagenesis libraries, 93 clones/site were picked, whereas for double site-saturation mutagenesis, a total of 930 clones were picked to oversample the respective library size by ~2.5-fold. Each 96-well deep well plate additionally contained three *Sc*SLAC clones carrying the parent of the library. Library clones were diluted 100-fold into 400 μl autoinduction media with 50 μg ml^−1^ kanamycin, 10 μg ml^−1^ tetracycline and 6 mM **C6a**. Precultures were additionally diluted 1:1 with 50% glycerol, snap frozen and stored at –80 °C. Cultures were grown at 37 °C for 8 h and additionally at 25 °C for 20 h. The cells were collected by centrifugation (10 min, 4 °C, 4,200*g*), the supernatant was decanted and the cells were frozen at –80 °C.

Cell pellets were thawed at room temperature and lysed with lysis buffer (150 µl, 20 mM Tris, 500 mM NaCl, pH 8.0 at 4 °C, 0.25% *n*-octyl β-d-thioglucopyranoside, 2 mg ml^−1^ lysozyme, 2 mM CuCl_2_) at 37 °C for 3 h. The lysate was clarified by centrifugation (4,384*g*, 4 °C, 15 min), and 10 µl was used for the screening. The reactions were carried out in 20 mM Britton–Robinson buffer (20 mM acetic acid, 20 mM phosphoric acid, 20 mM boric acid, pH adjusted with aqueous NaOH) at pH 7.0. The following reaction components were mixed together in a reaction volume of 100 µl: 50 µM Amplex Red, 500 µM CuCl_2_ and lysate. The reactions were incubated at 30 °C for 1 h, and the activity was measured by fluorescence (excitation wavelength *λ*_ex_, 560 nm; emission wavelength *λ*_em_, 590 nm).

### Michaelis–Menten kinetics with Amplex Red

The reactions were carried out in 20 mM Britton–Robinson buffer (20 mM acetic acid, 20 mM phosphoric acid, 20 mM boric acid, pH adjusted with aqueous NaOH) at pH 7.0. The following reaction components were mixed together in a reaction volume of 100 µl: Amplex Red (25, 50, 100, 200, 400 or 600 µM), 500 µM CuCl_2_ and enzyme (0.2 µM for M298**C5a**, M298**C6a** and PW**C6a**, and 1 µM for wt). As a control, samples without enzyme were prepared. For the calibration curve, resorufin (0, 0.5, 1, 2, 4, 6, 8 or 10 µM) was used. After mixing by pipetting up and down, the fluorescence change (*λ*_ex_, 560 nm; *λ*_em_, 590 nm) was measured every minute for 1 h. The reaction was carried out at 25 °C. For data processing, for each Amplex Red concentration, the time trace corresponding to the negative control (without enzyme) was subtracted from each time trace containing protein. The calibration curve was fitted with a polynomial fit second order and was used to calculate the amount of product formed. For each concentration, the slopes for all ten consecutive timepoints were calculated, and the highest value was used as *k*_obs_. The kinetic parameters were determined by Michaelis–Menten analysis.

### Calculated log*P* determination

The calculated log*P* of the ncAAs was calculated from the SMILES (Simplified Molecular Input Line Entry System) using the RDKit package in Python (RDKit, open-source cheminformatics https://www.rdkit.org). The Python code is available for use (as described in the ‘Code availability’ statement)^105^.

### Feature analysis of laccase crystal structures

Crystal structures were extracted from the PDB based on the search term ‘laccase’ (Supplementary Table 7). Starting structures were manually processed to remove all heteroatoms and water molecules except for the T1Cu and the TNC; symmetry mates were searched to find the correct oligomeric structure for each laccase. The manually processed structures were further automatically processed in the following ways: REDUCE^108^ to add hydrogen atoms, automated identification and coordinate alignment of the T1Cu binding site. All atoms within a 10 Å radius of the copper were extracted for subsequent analysis. Molecular electrostatic potentials were calculated using APBS^109^ following PDB no. 2PQR (refs. ^110,111^) conversion with the PARSE^112^ force field (**C6a** values were taken from SwissSidechain^113^). Molecular lipophilicity potentials were generated using PyMLP^114^ (**C6a** values were determined from RDKit computed log*P* values, and atomistic assignments were based on canonical amino acids^114^), and cavity properties including volume and average cavity hydropathy were determined using PyKVFinder^115^ with a 1.2 Å probe radius. Physicochemical parameters were extracted to describe the area around the T1Cu. Comparison of these parameters was used to identify features that were statistically significant in discriminating between the wt bacterial and fungal laccases (18 wt bacterial and 19 fungal structures; Mann–Whitney *U* test for *P* values and common language effect size magnitude for effect size). Uniform manifold approximation and projection (UMAP) and *t*-distributed stochastic neighbor embedding (*t*-SNE) were used to visualize and quantify the difference between the variants. M298**C6a** was determined to be the least similar to bacterial laccases and the most similar to fungal laccases. The preprocessed PDB files and Python code are available for use (as described in the ‘Code availability’ statement)^105^.

### Reporting summary

Further information on research design is available in the Nature Portfolio Reporting Summary linked to this article.

## Online content

Any methods, additional references, Nature Portfolio reporting summaries, source data, extended data, supplementary information, acknowledgements, peer review information; details of author contributions and competing interests; and statements of data and code availability are available at 10.1038/s41557-026-02116-7.

## Supplementary information

Supplementary Information Supplementary Figs. 1–24; Tables 1–7; DNA, protein and plasmid sequences; and synthetic methods.

Reporting Summary

Supplementary Data 1 Sequence data for primers, coding regions, proteins and plasmids.

Supplementary Data 2 Code related to log*P* prediction, PDB analysis and redox deconvolution.

Supplementary Data 3 Source data for Supplementary Information figures.

## Source data

Source Data Fig. 1 Fluorescence and OD_600_ data for engineering the aaRS/tRNA pair; LC-MS spectra of sfGFP containing **C5a**, **C6a** or **C7a**; and LC-MS/MS spectrum of sfGFP containing **C6a**.

Source Data Fig. 2 Hydrophobicity of T1Cu sites, protein titre, extinction coefficient spectra and redox titration, as well as raw data for extinction coefficient and redox titration experiments.

Source Data Fig. 3 Kinetics and TTN analysis.

Source Data Fig. 5 Kinetics and TTN analysis.

Source Data Extended Data Fig. 1 Calculated log*P* and convex hull volumes for amino acids.

Source Data Extended Data Fig. 2 EPR spectra and extracted parameters.

Source Data Extended Data Fig. 3 TTNs for preliminary hits from directed evolution.

Source Data Extended Data Fig. 4 Comparison of UV–vis spectra, TTNs and kinetics for M298, M298**C6a** and PW**C6a**.

## Figures and Tables

**Extended Data Table 1:**
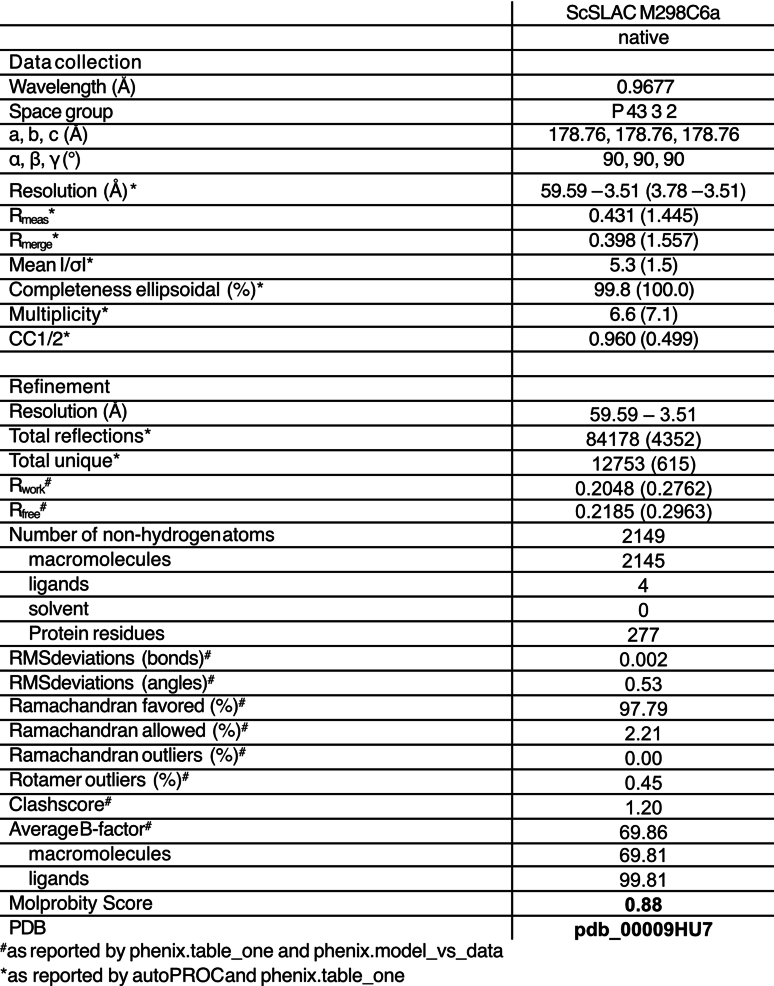
Crystallography: data collection and refinement statistics

## Data Availability

The sequence data, source data, raw data, input data, output data and crystallographic data are available via Zenodo at 10.5281/zenodo.18271154 (ref. ^105^). DNA and protein sequences, including primer sequences, are provided in Supplementary Data 1. The crystallographic data for *Sc*SLAC M298**C6a** have also been deposited at the PDB under accession no. 9HU7 and are available at https://proteindiffraction.org. All reported plasmid sequences are deposited in GenBank, and the IDs are referenced in Methods and Supplementary Information. The plasmids pGS1T-PylRS^C6a^–PylT^m15^_CUA_, pGS1T-PylRS^C6a^–*Mm*tRNA^Pyl^_CUA_ and pET28-*Sc*SLAC-6xHis are available through Addgene (nos. 251554, 251555 and 251556, respectively). Source data are provided with this paper.

## Code availability

The code used for determining log*P*, deconvolution of redox titration spectra and comparison of T1Cu environments of bacterial and fungal laccases is available via Zenodo at 10.5281/zenodo.18271154 (ref. ^105^). The code is also provided as Supplementary Data 2.

## References

[1] Estell, D. A. et al. Probing steric and hydrophobic effects on enzyme-substrate interactions by protein engineering. *Science* **233**, 659–663 (1986). 17835820 10.1126/science.233.4764.659

[2] Benkovic, S. J., Fierke, C. A. & Naylor, A. M. Insights into enzyme function from studies on mutants of dihydrofolate reductase. *Science* **239**, 1105–1110 (1988). 3125607 10.1126/science.3125607

[3] Garner, D. K. et al. Reduction potential tuning of the blue copper center in *Pseudomonas aeruginosa* azurin by the axial methionine as probed by unnatural amino acids. *J. Am. Chem. Soc.* **128**, 15608–15617 (2006). 17147368 10.1021/ja062732i

[4] Fierke, C. A., Calderone, T. L. & Krebs, J. F. Functional consequences of engineering the hydrophobic pocket of carbonic anhydrase II. *Biochemistry* **30**, 11054–11063 (1991). 1657158 10.1021/bi00110a007

[5] Eom, H., Cao, Y., Kim, H., de Visser, S. P. & Song, W. J. Underlying role of hydrophobic environments in tuning metal elements for efficient enzyme catalysis. *J. Am. Chem. Soc.* **145**, 5880–5887 (2023). 36853654 10.1021/jacs.2c13337

[6] Hunt, J. A., Ahmed, M. & Fierke, C. A. Metal binding specificity in carbonic anhydrase is influenced by conserved hydrophobic core residues. *Biochemistry* **38**, 9054–9062 (1999). 10413479 10.1021/bi9900166

[7] Ortmayer, M. et al. Rewiring the “push-pull” catalytic machinery of a heme enzyme using an expanded genetic code. *ACS Catal.* **10**, 2735–2746 (2020). 32550044 10.1021/acscatal.9b05129 PMC7273622

[8] Ortmayer, M. et al. A noncanonical tryptophan analogue reveals an active site hydrogen bond controlling ferryl reactivity in a heme peroxidase. *JACS Au* **1**, 913–918 (2021). 34337604 10.1021/jacsau.1c00145 PMC8317151

[9] Perdiguero, A. N. & Liang, A. D. Practical approaches to genetic code expansion with aminoacyl-tRNA synthetase/tRNA pairs. *CHIMIA* **78**, 22–31 (2024). 38430060 10.2533/chimia.2024.22

[10] Huguenin-Dezot, N. et al. Trapping biosynthetic acyl-enzyme intermediates with encoded 2,3-diaminopropionic acid. *Nature* **565**, 112–117 (2019). 30542153 10.1038/s41586-018-0781-z PMC6436733

[11] Drienovská, I., Rioz-Martínez, A., Draksharapu, A. & Roelfes, G. Novel artificial metalloenzymes by *in vivo* incorporation of metal-binding unnatural amino acids. *Chem. Sci.* **6**, 770–776 (2014). 28936318 10.1039/c4sc01525h PMC5590542

[12] Mayer, C., Dulson, C., Reddem, E., Thunnissen, A.-M. W. H. & Roelfes, G. Directed evolution of a designer enzyme featuring an unnatural catalytic amino acid. *Angew. Chem. Int. Ed.* **58**, 2083–2087 (2019). 10.1002/anie.201813499 PMC6519144 30575260

[13] Burke, A. J. et al. Design and evolution of an enzyme with a non-canonical organocatalytic mechanism. *Nature* **570**, 219–223 (2019). 31132786 10.1038/s41586-019-1262-8

[14] Trimble, J. S. et al. A designed photoenzyme for enantioselective [2+2] cycloadditions. *Nature* **611**, 709–714 (2022). 36130727 10.1038/s41586-022-05335-3

[15] Jackson, J. C., Duffy, S. P., Hess, K. R. & Mehl, R. A. Improving nature’s enzyme active site with genetically encoded unnatural amino acids. *J. Am. Chem. Soc.* **128**, 11124–11127 (2006). 16925430 10.1021/ja061099y

[16] Ugwumba, I. N. et al. Improving a natural enzyme activity through incorporation of unnatural amino acids. *J. Am. Chem. Soc.* **133**, 326–333 (2011). 21162578 10.1021/ja106416g

[17] Green, A. P., Hayashi, T., Mittl, P. R. E. & Hilvert, D. A chemically programmed proximal ligand enhances the catalytic properties of a heme enzyme. *J. Am. Chem. Soc.* **138**, 11344–11352 (2016). 27500802 10.1021/jacs.6b07029

[18] Pott, M. et al. A noncanonical proximal heme ligand affords an efficient peroxidase in a globin fold. *J. Am. Chem. Soc.* **140**, 1535–1543 (2018). 29309143 10.1021/jacs.7b12621

[19] Berry, S. M., Ralle, M., Low, D. W., Blackburn, N. J. & Lu, Y. Probing the role of axial methionine in the blue copper center of azurin with unnatural amino acids. *J. Am. Chem. Soc.* **125**, 8760–8768 (2003). 12862470 10.1021/ja029699u

[20] Mendel, D. et al. Probing protein stability with unnatural amino acids. *Science* **256**, 1798–1802 (1992). 1615324 10.1126/science.1615324

[21] Welsh, J. P., Patel, K. G., Manthiram, K. & Swartz, J. R. Multiply mutated *Gaussia* luciferases provide prolonged and intense bioluminescence. *Biochem. Biophys. Res. Commun.* **389**, 563–568 (2009). 19825431 10.1016/j.bbrc.2009.09.006

[22] Cirino, P. C., Tang, Y., Takahashi, K., Tirrell, D. A. & Arnold, F. H. Global incorporation of norleucine in place of methionine in cytochrome P450 BM-3 heme domain increases peroxygenase activity. *Biotechnol. Bioeng.* **83**, 729–734 (2003). 12889037 10.1002/bit.10718

[23] Hoesl, M. G. et al. Lipase congeners designed by genetic code engineering. *ChemCatChem* **3**, 213–221 (2011).

[24] Horton, K. L., Stewart, K. M., Fonseca, S. B., Guo, Q. & Kelley, S. O. Mitochondria-penetrating peptides. *Chem. Biol.* **15**, 375–382 (2008). 18420144 10.1016/j.chembiol.2008.03.015

[25] Kaganman, I. Pony express to mitochondria. *Nat. Methods* **5**, 468 (2008).

[26] Nomura, T. et al. Probing phenylalanine environments in oligomeric structures with pentafluorophenylalanine and cyclohexylalanine. *Biopolymers* **95**, 410–419 (2011). 21280026 10.1002/bip.21594

[27] Haerianardakani, S. et al. Phenylalanine mutation to cyclohexylalanine facilitates triangular trimer formation by β-hairpins derived from Aβ. *J. Am. Chem. Soc.* **142**, 20708–20716 (2020). 33237748 10.1021/jacs.0c09281 PMC7821965

[28] Harrison, K. et al. Exploiting hydrophobic amino acid scanning to develop cyclic peptide inhibitors of the SARS-CoV-2 main protease with antiviral activity. *Chem. Eur. J.* **30**, e202401606 (2024). 38801240 10.1002/chem.202401606

[29] Dunkelmann, D. L. & Chin, J. W. Engineering pyrrolysine systems for genetic code expansion and reprogramming. *Chem. Rev.* **124**, 11008–11062 (2024). 39235427 10.1021/acs.chemrev.4c00243 PMC11467909

[30] Yanagisawa, T. et al. Multistep engineering of pyrrolysyl-tRNA synthetase to genetically encode *N*^ɛ^-(*o*-azidobenzyloxycarbonyl) lysine for site-specific protein modification. *Chem. Biol.* **15**, 1187–1197 (2008). 19022179 10.1016/j.chembiol.2008.10.004

[31] Serfling, R. et al. Designer tRNAs for efficient incorporation of non-canonical amino acids by the pyrrolysine system in mammalian cells. *Nucleic Acids Res.* **46**, 1–10 (2018). 29177436 10.1093/nar/gkx1156 PMC5758916

[32] Jones, S. M. & Solomon, E. I. Electron transfer and reaction mechanism of laccases. *Cell. Mol. Life Sci.* **72**, 869–883 (2015). 25572295 10.1007/s00018-014-1826-6 PMC4323859

[33] Solomon, E. I., Sundaram, U. M. & Machonkin, T. E. Multicopper oxidases and oxygenases. *Chem. Rev.* **96**, 2563–2606 (1996). 11848837 10.1021/cr950046o

[34] Lange, H., Decina, S. & Crestini, C. Oxidative upgrade of lignin – recent routes reviewed. *Eur. Polym. J.* **49**, 1151–1173 (2013).

[35] Munk, L., Sitarz, A. K., Kalyani, D. C., Mikkelsen, J. D. & Meyer, A. S. Can laccases catalyze bond cleavage in lignin?. *Biotechnol. Adv.* **33**, 13–24 (2015). 25560931 10.1016/j.biotechadv.2014.12.008

[36] Witayakran, S. & Ragauskas, A. J. Synthetic applications of laccase in green chemistry. *Adv. Synth. Catal.* **351**, 1187–1209 (2009).

[37] Obermaier, S., Thiele, W., Fürtges, L. & Müller, M. Enantioselective phenol coupling by laccases in the biosynthesis of fungal dimeric naphthopyrones. *Angew. Chem. Int. Ed.* **58**, 9125–9128 (2019). 10.1002/anie.201903759 31050129

[38] Gunne, M., Höppner, A., Hagedoorn, P.-L. & Urlacher, V. B. Structural and redox properties of the small laccase Ssl1 from *Streptomyces sviceus*. *FEBS J.* **281**, 4307–4318 (2014). 24548692 10.1111/febs.12755

[39] Olbrich, A. C., Schild, J. N. & Urlacher, V. B. Correlation between the T1 copper reduction potential and catalytic activity of a small laccase. *J. Inorg. Biochem.* **201**, 110843 (2019). 31536948 10.1016/j.jinorgbio.2019.110843

[40] Xu, F. et al. Targeted mutations in a *Trametes villosa* laccase: axial perturbations of the T1 copper. *J. Biol. Chem.* **274**, 12372–12375 (1999). 10212209 10.1074/jbc.274.18.12372

[41] Durão, P. et al. Perturbations of the T1 copper site in the CotA laccase from *Bacillus subtilis*: structural, biochemical, enzymatic and stability studies. *J. Biol. Inorg. Chem.* **11**, 514 (2006). 16680453 10.1007/s00775-006-0102-0

[42] Zovo, K. et al. Substitution of the methionine axial ligand of the T1 copper for the fungal-like phenylalanine ligand (M298F) causes local structural perturbations that lead to thermal instability and reduced catalytic efficiency of the small laccase from *Streptomyces coelicolor* A3(2). *ACS Omega* **7**, 6184–6194 (2022). 35224382 10.1021/acsomega.1c06668 PMC8867573

[43] Tepper, A. W. J. W. et al. Identification of a radical intermediate in the enzymatic reduction of oxygen by a small laccase. *J. Am. Chem. Soc.* **131**, 11680–11682 (2009). 19645472 10.1021/ja900751c

[44] Xu, F. Oxidation of phenols, anilines, and benzenethiols by fungal laccases: correlation between activity and redox potentials as well as halide inhibition. *Biochemistry* **35**, 7608–7614 (1996). 8652543 10.1021/bi952971a

[45] Marcus, R. A. Electron transfer reactions in chemistry: theory and experiment*. Angew. Chem. Int. Ed.* **32**, 1111–1121 (1993).

[46] Sakurai, T. & Kataoka, K. Structure and function of type I copper in multicopper oxidases. *Cell. Mol. Life Sci.* **64**, 2642 (2007). 17639274 10.1007/s00018-007-7183-y PMC11136192

[47] Margot, J. et al. Bacterial *versus* fungal laccase: potential for micropollutant degradation. *AMB Express* **3**, 63 (2013). 24152339 10.1186/2191-0855-3-63 PMC3819643

[48] Singh, M., Singh, R. & Chopra, C. Characterization, applications, and industrial potential of bacterial laccases: a comprehensive study. *Biol. Forum Int. J.* **15**, 1046–1051 (2023).

[49] Chauhan, P. S., Goradia, B. & Saxena, A. Bacterial laccase: recent update on production, properties and industrial applications. *3 Biotech* **7**, 323 (2017). 28955620 10.1007/s13205-017-0955-7 PMC5602783

[50] Miura, Y. et al. Direct electrochemistry of CueO and its mutants at residues to and near type I Cu for oxygen-reducing biocathode. *Fuel Cells* **9**, 70–78 (2009).

[51] Kataoka, K. et al. Enhancement of laccase activity through the construction and breakdown of a hydrogen bond at the type I copper center in *Escherichia coli* CueO and the deletion mutant Δα5−7 CueO. *Biochemistry* **50**, 558–565 (2011). 21142169 10.1021/bi101107c

[52] Kataoka, K., Kogi, H., Tsujimura, S. & Sakurai, T. Modifications of laccase activities of copper efflux oxidase, CueO by synergistic mutations in the first and second coordination spheres of the type I copper center. *Biochem. Biophys. Res. Commun.* **431**, 393–397 (2013). 23337502 10.1016/j.bbrc.2013.01.040

[53] Zhang, L. et al. Engineering of laccase CueO for improved electron transfer in bioelectrocatalysis by semi-rational design. *Chem. Eur. J.* **26**, 4974–4979 (2020). 31985091 10.1002/chem.201905598 PMC7186830

[54] Wang, J.-X. et al. Increasing reduction potentials of type 1 copper center and catalytic efficiency of small laccase from *Streptomyces coelicolor* through secondary coordination sphere mutations. *Angew. Chem. Int. Ed.* **62**, e202314019 (2023). 10.1002/anie.202314019 PMC10842694 37926680

[55] Wang, J.-X. et al. Unexpected effect of an axial ligand mutation in the type 1 copper center in small laccase: structure-based analyses and engineering to increase reduction potential and activity. *Chem. Sci.* **16**, 11339–11346 (2025). 40438172 10.1039/d5sc02177d PMC12109610

[56] Eby, M. et al. *Optimization and Application of a Small Laccase (SLAC) for Enzymatic Fuel Cell Development* (Air Force Civil Engineer Center, 2016).

[57] Mate, D. M. & Alcalde, M. Laccase engineering: from rational design to directed evolution. *Biotechnol. Adv.* **33**, 25–40 (2015). 25545886 10.1016/j.biotechadv.2014.12.007

[58] Kurose, S. et al. Modification of spectroscopic properties and catalytic activity of *Escherichia coli* CueO by mutations of methionine 510, the axial ligand to the type I Cu. *Bull. Chem. Soc. Jpn* **82**, 504–508 (2009).

[59] Mukai, T. et al. Highly reproductive *Escherichia coli* cells with no specific assignment to the UAG codon. *Sci. Rep.* **5**, 9699 (2015). 25982672 10.1038/srep09699 PMC4434889

[60] Eddins, A. J. et al. Truncation-free genetic code expansion with tetrazine amino acids for quantitative protein ligations. *Bioconjug. Chem.* **34**, 2243–2254 (2023). 38047550 10.1021/acs.bioconjchem.3c00380 PMC11641772

[61] Solomon, E. I. Spectroscopic methods in bioinorganic chemistry: blue to green to red copper sites. *Inorg. Chem.* **45**, 8012–8025 (2006). 16999398 10.1021/ic060450d

[62] Olbrich, A. C. et al. Substitution of the axial type 1 Cu ligand affords binding of a water molecule in axial position affecting kinetics, spectral, and structural properties of the small laccase Ssl1. *Chem. Eur. J.* **31**, e202403005 (2025). 39541228 10.1002/chem.202403005

[63] Solomon, E. I. & Hadt, R. G. Recent advances in understanding blue copper proteins. *Coord. Chem. Rev.* **255**, 774–789 (2011).

[64] Solomon, E. I., Szilagyi, R. K., DeBeer George, S. & Basumallick, L. Electronic structures of metal sites in proteins and models: contributions to function in blue copper proteins. *Chem. Rev.* **104**, 419–458 (2004). 14871131 10.1021/cr0206317

[65] Yu, S.-S. et al. Structural basis for a quadratic relationship between electronic absorption and electronic paramagnetic resonance parameters of type 1 copper proteins. *Inorg. Chem.* **59**, 10620–10627 (2020). 32689800 10.1021/acs.inorgchem.0c01065

[66] Van Stappen, C. et al. Beyond blue: systematic modulation of electronic structure and redox properties of type 1 copper in azurin. *J. Am. Chem. Soc.* **147**, 24825–24837 (2025). 40626760 10.1021/jacs.5c07009 PMC13489697

[67] Bím, D. & N. Alexandrova, A. Electrostatic regulation of blue copper sites. *Chem. Sci.* **12**, 11406–11413 (2021). 34667549 10.1039/d1sc02233d PMC8447924

[68] Palmer, A. E., Randall, D. W., Xu, F. & Solomon, E. I. Spectroscopic studies and electronic structure description of the high potential type 1 copper site in fungal laccase: insight into the effect of the axial ligand. *J. Am. Chem. Soc.* **121**, 7138–7149 (1999).

[69] Garzillo, A. M. et al. Structural and kinetic characterization of native laccases from *Pleurotus ostreatus*, *Rigidoporus lignosus*, and *Trametes trogii*. *J. Protein Chem.* **20**, 191–201 (2001). 11565899 10.1023/a:1010954812955

[70] Gallaway, J. et al. Oxygen-reducing enzyme cathodes produced from SLAC, a small laccase from *Streptomyces coelicolor*. *Biosens. Bioelectron.* **23**, 1229–1235 (2008). 18096378 10.1016/j.bios.2007.11.004

[71] Toscano, M. D., De Maria, L., Lobedanz, S. & Østergaard, L. H. Optimization of a small laccase by active-site redesign. *ChemBioChem* **14**, 1209–1211 (2013). 23775916 10.1002/cbic.201300256

[72] Yu, L. et al. Analysis of the electron transfer pathway in small laccase by EPR and UV–vis spectroscopy coupled with redox titration. *Magn. Reson. Lett.* **4**, 200116 (2024). 40919590 10.1016/j.mrl.2024.200116 PMC12406506

[73] Scott, S. L., Chen, W. J., Bakac, A. & Espenson, J. H. Spectroscopic parameters, electrode potentials, acid ionization constants, and electron exchange rates of the 2,2′-azinobis(3-ethylbenzothiazoline-6-sulfonate) radicals and ions. *J. Phys. Chem.* **97**, 6710–6714 (1993).

[74] Pavitt, A. S., Bylaska, E. J. & Tratnyek, P. G. Oxidation potentials of phenols and anilines: correlation analysis of electrochemical and theoretical values. *Environ. Sci. Process. Impacts* **19**, 339–349 (2017). 28229145 10.1039/c6em00694a

[75] Wood, P. M. The potential diagram for oxygen at pH 7. *Biochem. J.* **253**, 287–289 (1988). 2844170 10.1042/bj2530287 PMC1149288

[76] den Boer, D., de Heer, H. C., Buda, F. & Hetterscheid, D. G. H. Challenges in elucidating the free energy scheme of the laccase catalyzed reduction of oxygen. *ChemCatChem* **15**, e202200878 (2023). 37082113 10.1002/cctc.202200878 PMC10107611

[77] Lipmann, F. in *Advances in Enzymology and Related Areas of Molecular Biology*, Vol. 1 (eds Nord, F. F. & Werkman, C. H.) Ch. 4 (John Wiley & Sons 1941); 10.1002/9780470122464.ch4

[78] Kalckar, H. M. The nature of energetic coupling in biological syntheses. *Chem. Rev.* **28**, 71–178 (1941).

[79] Sharp, R. E. & Chapman, S. K. Mechanisms for regulating electron transfer in multi-centre redox proteins. *Biochim. Biophys. Acta Protein Struct. Mol. Enzymol.* **1432**, 143–158 (1999). 10.1016/s0167-4838(99)00109-0 10407138

[80] Tadesse, M. A., D’Annibale, A., Galli, C., Gentili, P. & Sergi, F. An assessment of the relative contributions of redox and steric issues to laccase specificity towards putative substrates. *Org. Biomol. Chem.* **6**, 868–878 (2008). 18292878 10.1039/b716002j

[81] Squillacote, M., Sheridan, R. S., Chapman, O. L. & Anet, F. A. L. Spectroscopic detection of the twist-boat conformation of cyclohexane. Direct measurement of the free energy difference between the chair and the twist-boat. *J. Am. Chem. Soc.* **97**, 3244–3246 (1975).

[82] Wang, T., Xiang, Y., Liu, X., Chen, W. & Hu, Y. A novel fluorimetric method for laccase activities measurement using Amplex Red as substrate. *Talanta* **162**, 143–150 (2017). 27837810 10.1016/j.talanta.2016.10.006

[83] Dębski, D. et al. Mechanism of oxidative conversion of Amplex® Red to resorufin: pulse radiolysis and enzymatic studies. *Free Radic. Biol. Med.* **95**, 323–332 (2016). 10.1016/j.freeradbiomed.2016.03.027 PMC5697983 27021961

[84] Olson, C. A., Wu, N. C. & Sun, R. A comprehensive biophysical description of pairwise epistasis throughout an entire protein domain. *Curr. Biol.* **24**, 2643–2651 (2014). 25455030 10.1016/j.cub.2014.09.072 PMC4254498

[85] Mateljak, I. et al. Increasing redox potential, redox mediator activity, and stability in a fungal laccase by computer-guided mutagenesis and directed evolution. *ACS Catal.* **9**, 4561–4572 (2019).

[86] Barber-Zucker, S. et al. Designed high-redox potential laccases exhibit high functional diversity. *ACS Catal.* **12**, 13164–13173 (2022). 36366766 10.1021/acscatal.2c03006 PMC9638991

[87] Maté, D. et al. Laboratory evolution of high-redox potential laccases. *Chem. Biol.* **17**, 1030–1041 (2010). 20851352 10.1016/j.chembiol.2010.07.010

[88] Rogers, J. M., Passioura, T. & Suga, H. Nonproteinogenic deep mutational scanning of linear and cyclic peptides. *Proc. Natl Acad. Sci. USA* **115**, 10959–10964 (2018). 30301798 10.1073/pnas.1809901115 PMC6205457

[89] Bovy, P. R. et al. A synthetic linear decapeptide binds to the atrial natriuretic peptide receptors and demonstrates cyclase activation and vasorelaxant activity. *J. Biol. Chem.* **264**, 20309–20313 (1989). 2555353

[90] Chatenet, D. et al. Structure−activity relationships of a novel series of urotensin ii analogues: identification of a urotensin II antagonist. *J. Med. Chem.* **49**, 7234–7238 (2006). 17125276 10.1021/jm0602110

[91] Mukai, T. et al. Adding l-lysine derivatives to the genetic code of mammalian cells with engineered pyrrolysyl-tRNA synthetases. *Biochem. Biophys. Res. Commun.* **371**, 818–822 (2008). 18471995 10.1016/j.bbrc.2008.04.164

[92] Schneider, S. et al. Structural insights into incorporation of norbornene amino acids for click modification of proteins. *ChemBioChem* **14**, 2114–2118 (2013). 24027216 10.1002/cbic.201300435

[93] Miyake-Stoner, S. J. et al. Generating permissive site-specific unnatural aminoacyl-tRNA synthetases. *Biochemistry* **49**, 1667–1677 (2010). 20082521 10.1021/bi901947r

[94] Machczynski, M. C., Vijgenboom, E., Samyn, B. & Canters, G. W. Characterization of SLAC: a small laccase from *Streptomyces coelicolor* with unprecedented activity. *Protein Sci. Publ. Protein Soc.* **13**, 2388–2397 (2004). 10.1110/ps.04759104 PMC2280001 15295117

[95] Kielb, P. J., Teutloff, C., Bittl, R., Gray, H. B. & Winkler, J. R. Does tyrosine protect *S. coelicolor* laccase from oxidative degradation or act as an extended catalytic site?. *J. Phys. Chem. B* **126**, 7943–7949 (2022). 36191240 10.1021/acs.jpcb.2c04835 PMC10231039

[96] Vonrhein, C. et al. Data processing and analysis with the autoPROC toolbox. *Acta Crystallogr. D* **67**, 293–302 (2011). 10.1107/S0907444911007773 PMC3069744 21460447

[97] Kabsch, W. XDS. *Acta Crystallogr. D* **66**, 125–132 (2010). 10.1107/S0907444909047337 PMC2815665 20124692

[98] Evans, P. Scaling and assessment of data quality. *Acta Crystallogr. D* **62**, 72–82 (2006). 10.1107/S0907444905036693 16369096

[99] Evans, P. R. & Murshudov, G. N. How good are my data and what is the resolution? *Acta Crystallogr. D* **69**, 1204–1214 (2013). 10.1107/S0907444913000061 PMC3689523 23793146

[100] Agirre, J. et al. The *CCP4* suite: integrative software for macromolecular crystallography. *Acta Crystallogr. D* **79**, 449–461 (2023). 10.1107/S2059798323003595 PMC10233625 37259835

[101] Tickle, I. J. et al. The STARANISO server v.3.402 (Global Phasing Limited, 2016).

[102] Abramson, J. et al. Accurate structure prediction of biomolecular interactions with AlphaFold 3. *Nature* **630**, 493–500 (2024). 38718835 10.1038/s41586-024-07487-w PMC11168924

[103] Emsley, P., Lohkamp, B., Scott, W. G. & Cowtan, K. Features and development of *Coot*. *Acta Crystallogr. D* **66**, 486–501 (2010). 10.1107/S0907444910007493 PMC2852313 20383002

[104] Afonine, P. V. et al. Towards automated crystallographic structure refinement with *phenix.refine*. *Acta Crystallogr. D* **68**, 352–367 (2012). 10.1107/S0907444912001308 PMC3322595 22505256

[105] Fischer, S., Natter Perdiguero, A., Lau, K. & Deliz Liang, A. Data for “Hydrophobic tuning with non-canonical amino acids in a copper metalloenzyme”. *Zenodo*10.5281/zenodo.18271154 (2026). 10.1038/s41557-026-02116-7 PMC13323077 41974833

[106] Stoll, S. & Schweiger, A. EasySpin, a comprehensive software package for spectral simulation and analysis in EPR. *J. Magn. Reson.* **178**, 42–55 (2006). 16188474 10.1016/j.jmr.2005.08.013

[107] Fischer, J. W. A. et al. Design and performance of an oversized-sample 35 GHz EPR resonator with an elevated *Q* value. *Magn. Reson.* **5**, 143–152 (2024). 10.5194/mr-5-143-2024 PMC12178130 40539092

[108] Word, J. M., Lovell, S. C., Richardson, J. S. & Richardson, D. C. Asparagine and glutamine: using hydrogen atom contacts in the choice of side-chain amide orientation. *J. Mol. Biol.* **285**, 1735–1747 (1999). 9917408 10.1006/jmbi.1998.2401

[109] Jurrus, E. et al. Improvements to the APBS biomolecular solvation software suite. *Protein Sci.* **27**, 112–128 (2018). 28836357 10.1002/pro.3280 PMC5734301

[110] Dolinsky, T. J., Nielsen, J. E., McCammon, J. A. & Baker, N. A. PDB2PQR: an automated pipeline for the setup of Poisson–Boltzmann electrostatics calculations. *Nucleic Acids Res.* **32**, W665–W667 (2004). 15215472 10.1093/nar/gkh381 PMC441519

[111] Dolinsky, T. J. et al. PDB2PQR: expanding and upgrading automated preparation of biomolecular structures for molecular simulations. *Nucleic Acids Res.* **35**, W522–W525 (2007). 17488841 10.1093/nar/gkm276 PMC1933214

[112] Sitkoff, D., Sharp, K. A. & Honig, B. Accurate calculation of hydration free energies using macroscopic solvent models. *J. Phys. Chem.* **98**, 1978–1988 (1994).

[113] Gfeller, D., Michielin, O. & Zoete, V. SwissSidechain: a molecular and structural database of non-natural sidechains. *Nucleic Acids Res.* **41**, D327–D332 (2013). 23104376 10.1093/nar/gks991 PMC3531096

[114] Laguerre, M., Saux, M., Dubost, J. P. & Carpy, A. MLPP: a program for the calculation of molecular lipophilicity potential in proteins. *Pharm. Pharmacol. Commun.* **3**, 217–222 (1997).

[115] Guerra, J. V. daS. et al. pyKVFinder: an efficient and integrable Python package for biomolecular cavity detection and characterization in data science. *BMC Bioinf.* **22**, 607 (2021). 10.1186/s12859-021-04519-4 PMC8685811 34930115

