# Supplementary material for: Hydrophobic tuning with non-canonical amino acids in a copper metalloenzyme
Source: Nat Chem. 2026 Apr 13;18(7):1269–77. doi: 10.1038/s41557-026-02116-7 (PMC13323077; doi:10.1038/s41557-026-02116-7)
Supplement: Supplementary file 1 — Supplementary Figs. 1–24; Tables 1–7; DNA, protein and plasmid sequences; and synthetic methods. [file 41557_2026_2116_MOESM1_ESM.pdf]

nature chemistry

Supplementary information https://doi.org/10.1038/s41557-026-02116-7

Hydrophobic tuning with non-canonical amino acids in a copper metalloenzyme

In the format provided by the authors and unedited

I. Supplementary data figures

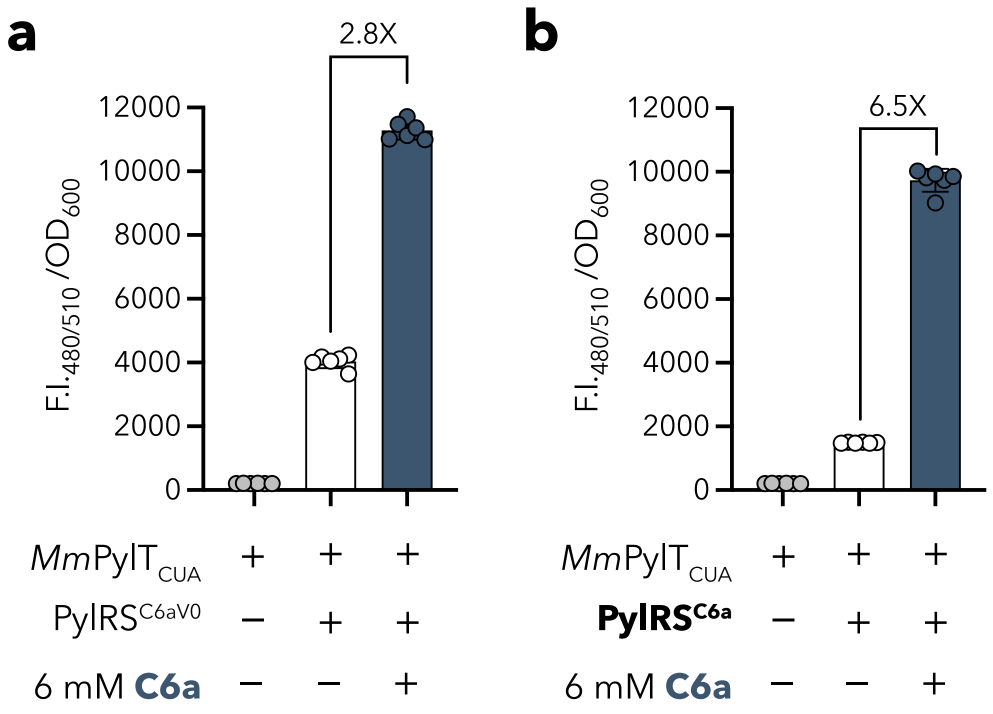

Supplementary Figure 1 | Validation of PylRS variants for incorporation of C6a. Suppression of sfGFP150$_{TAG}$ in NEB10β in the presence or absence of PylRS^{C6a_v0} and **C6a** (a) and in the presence or absence of PylRS^{C6a} and **C6a** (b). The data are presented as fluorescence (excitation at 480 nm and emission at 510 nm) normalized to the absorbance at 600 nm and represents the mean and standard deviation of 6 biological replicates.

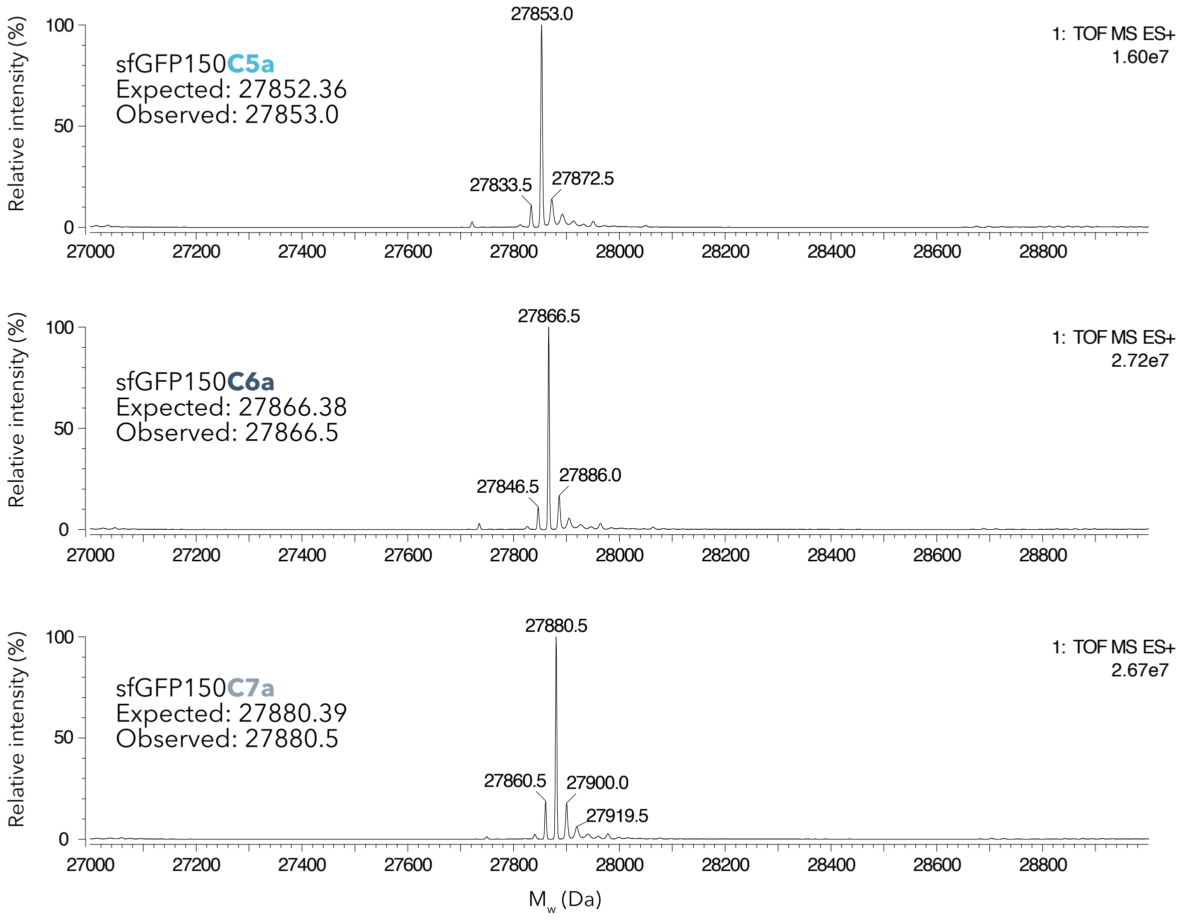

Supplementary Figure 2 | Full deconvolution spectra for LC-MS analysis of sfGFP150$_{TAG}$ containing C5a, C6a, or C7a. Spectra were collected for sfGFP150$_{TAG}$ expressed with pGS1T-PylRS^{C6a}-PylT^{m15}$_{CUA}$ in the presence of 12 mM of DL-**C5a** (top), 6 mM of **C6a** (middle), and 12 mM of DL-**C7a** (bottom).

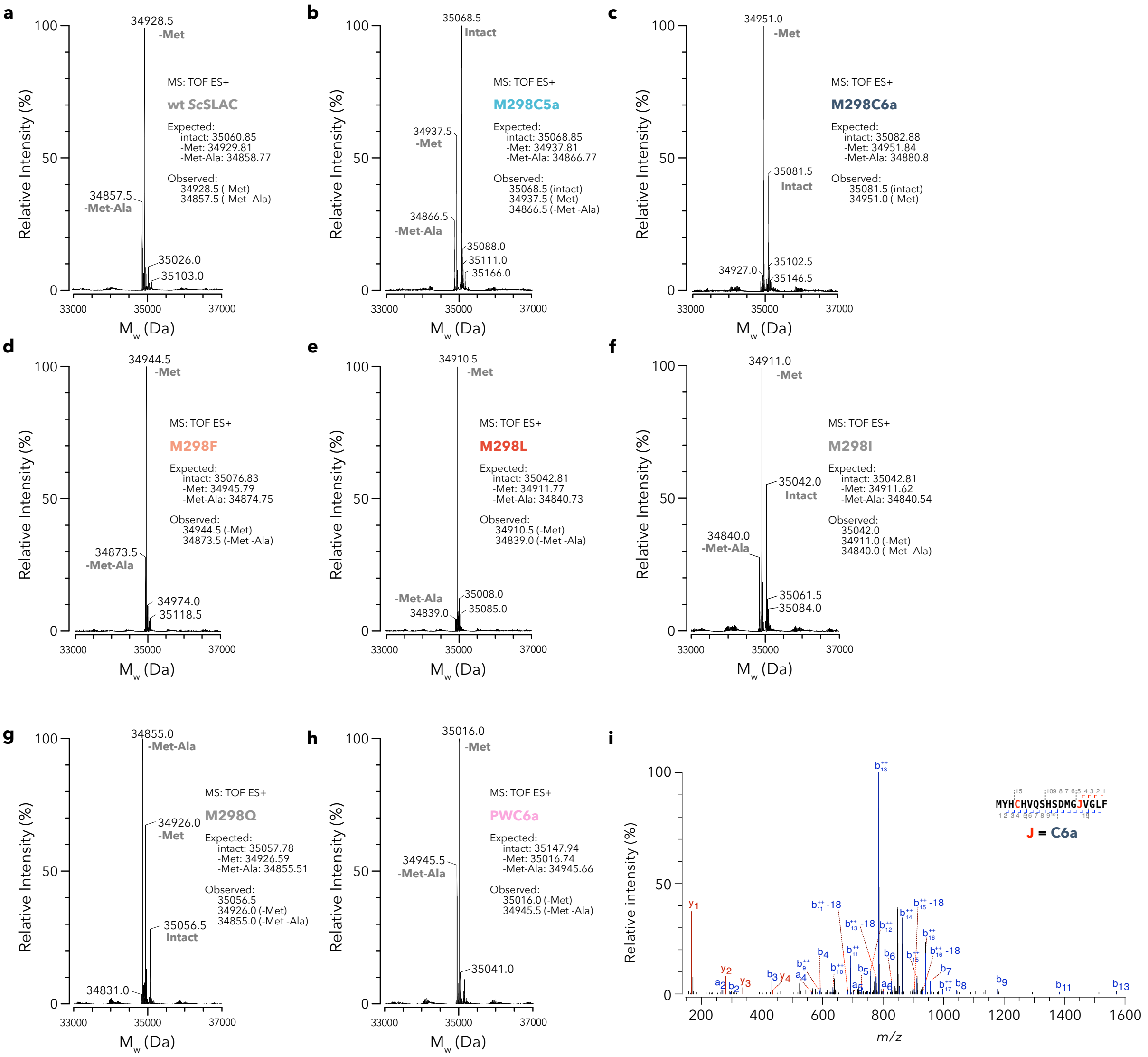

Supplementary Figure 3 | LC-MS and LC-MS/MS analysis of *Sc*SLAC-M298-variants. Mass spectra from LC-MS analysis of *Sc*SLAC variants: M298 (a), M298**C5a** (b), M298**C6a** (c), M298F (d), M298L (e), M298I (f), M298Q (g), and PW**C6a** (h). Often truncation of the N-terminal Met and MetAla is observed. A representative LC-MS/MS spectrum from a tryptic digest of M298**C6a** (i). Over 100 peptides containing **C6a** were observed, but no peptides for canonical amino acid incorporation were observed.

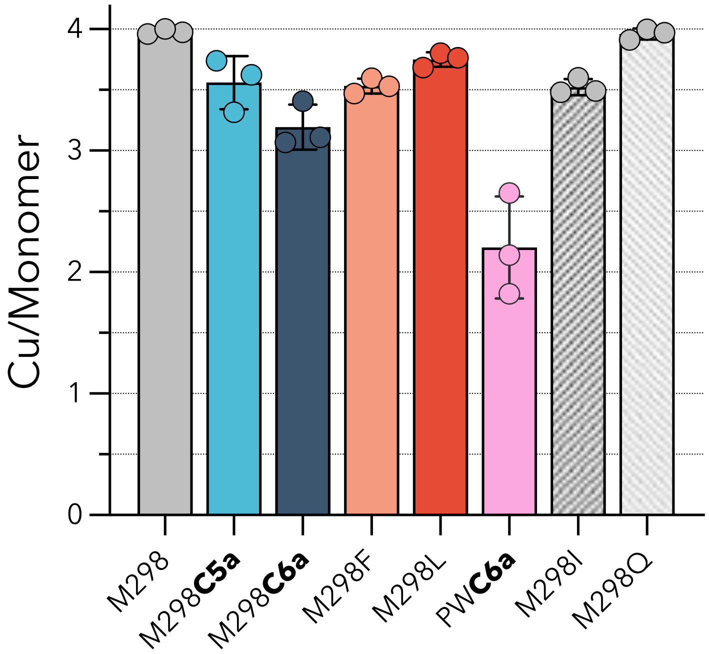

Supplementary Figure 4 | Metal content per enzyme of *Sc*SLAC variants. The copper content per monomer was measured with atomic absorbance spectroscopy (AAS). Measurements were performed in biological triplicates for all samples expect for M298I and M298Q that were performed in preparative triplicates. The data are represented as the mean and standard deviation of three replicates.

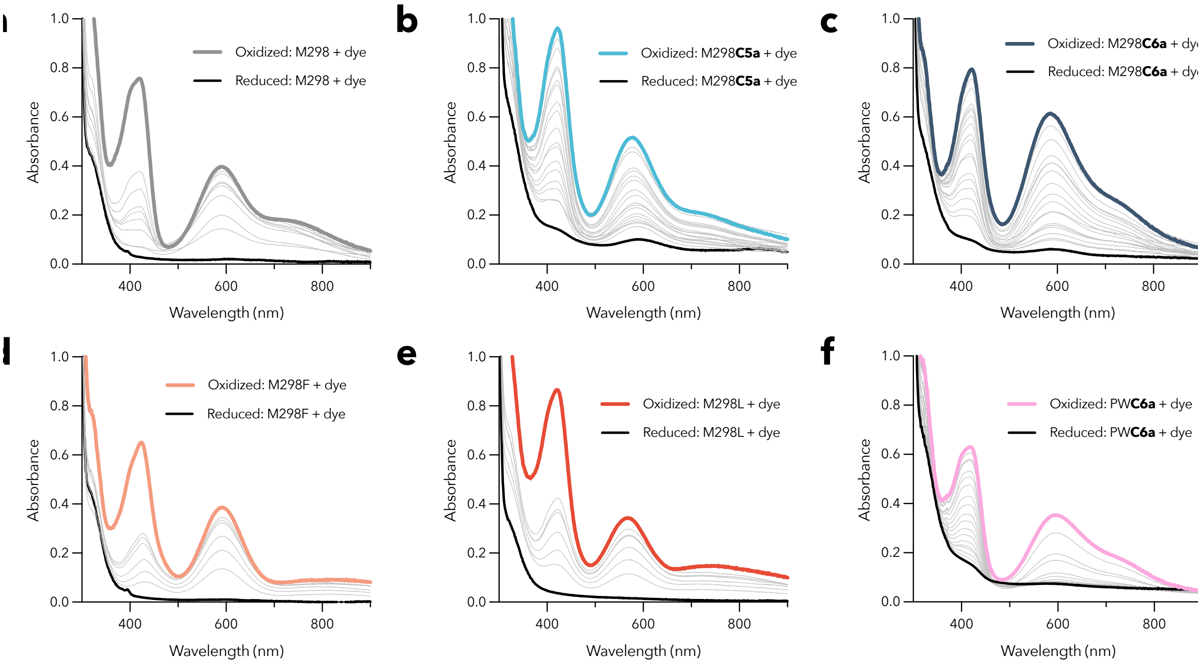

Supplementary Figure 5 | Redox titrations and analysis of the different *Sc*SLAC variants: M298, M298C5a, M298C6a, M298L, M298F, and PWC6a. For each variant, the UV-vis spectra for each titration point are shown in panels a-f. With these spectra, a deconvolution was conducted to calculate the ratio of reduced and oxidized dye and the ratio of reduced and oxidized T1Cu site. During titration of M298L, the dye was immediately and completely reduced before the protein, indicating that the $E°'_{T1Cu}$ of M298L is much lower than the dye. In contrast, PW**C6a** was immediately and completely reduced before the dye, indicating that the $E°'_{T1Cu}$ is much higher than the dye. Because there was no equilibrium between oxidation and reduction of the dye and M298L or PW**C6a**, it was not possible to determine the ratios of reduced and oxidized copper for the titrations of M298L and PW**C6a**, and therefore, the $E°'_{T1Cu}$ could not be precisely determined. Based on the indicator redox potential, the maximum absorbance attributed to the protein (0.40 AU) and dye (0.40 AU) in the fitted region (350-720 nm), and a detection limit estimate (0.010 AU), the theoretical maximum $E°'_{T1Cu}$ of M298L is 340 mV, and the theoretical minimum $E°'_{T1Cu}$ of PW**C6a** is 530 mV: $E^0_{dye} \pm \frac{RT}{zF} \cdot ln\left(\frac{Dye_{red}}{Dye_{ox}}\right) = 0.436\,V \pm \frac{8.314 \cdot 293}{1 \cdot 96485} ln\left(\frac{0.39}{0.01}\right)$ [116]. The $E°'_{T1Cu}$ measurements were performed in biological duplicates for M298**C5a** and M298**C6a**. A linear fit was applied according to Nernst equation: $E^0_{dye} - \frac{RT}{zF} \cdot ln\left(\frac{Dye_{red}}{Dye_{ox}}\right) = E^0_{T1Cu} - \frac{RT}{zF} \cdot ln\left(\frac{T1Cu_{red}}{T1Cu_{ox}}\right)$. The estimated $E°'_{T1Cu}$ is then given as the y-intercept (see Methods for a mathematical explanation). The factor of 1000 in the slope analysis is to account for the change from V to mV. g)

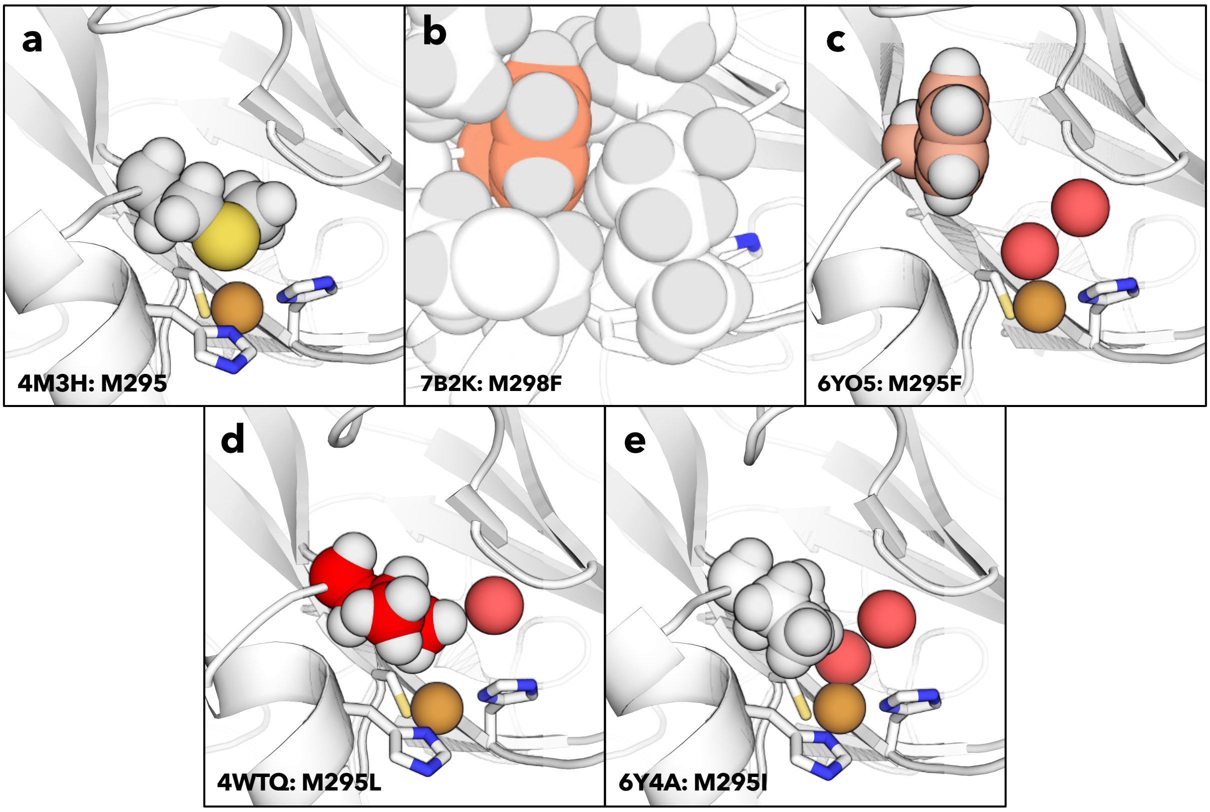

Supplementary Figure 6 | SLAC variants demonstrating water accumulation. Water accumulation in the active of fungal-like SLAC variants has been previously observed[42,55,62]. In the wt *Sc*SLAC, there is no pocket large enough for water within 5 Å of the T1Cu site (a). In axial phenylalanine mutants (ScSLAC, M298 and SLAC from *Streptomyces sviceus* M295), the phenyl ring of the side chain rotates away from the T1Cu site into a thin pocket adjacent to the T1Cu site creating a large cavity where water accumulates (b and c). For axial mutations to leucine (d) or isoleucine (e), the small side chain also creates large pockets allowing water to accumulate.

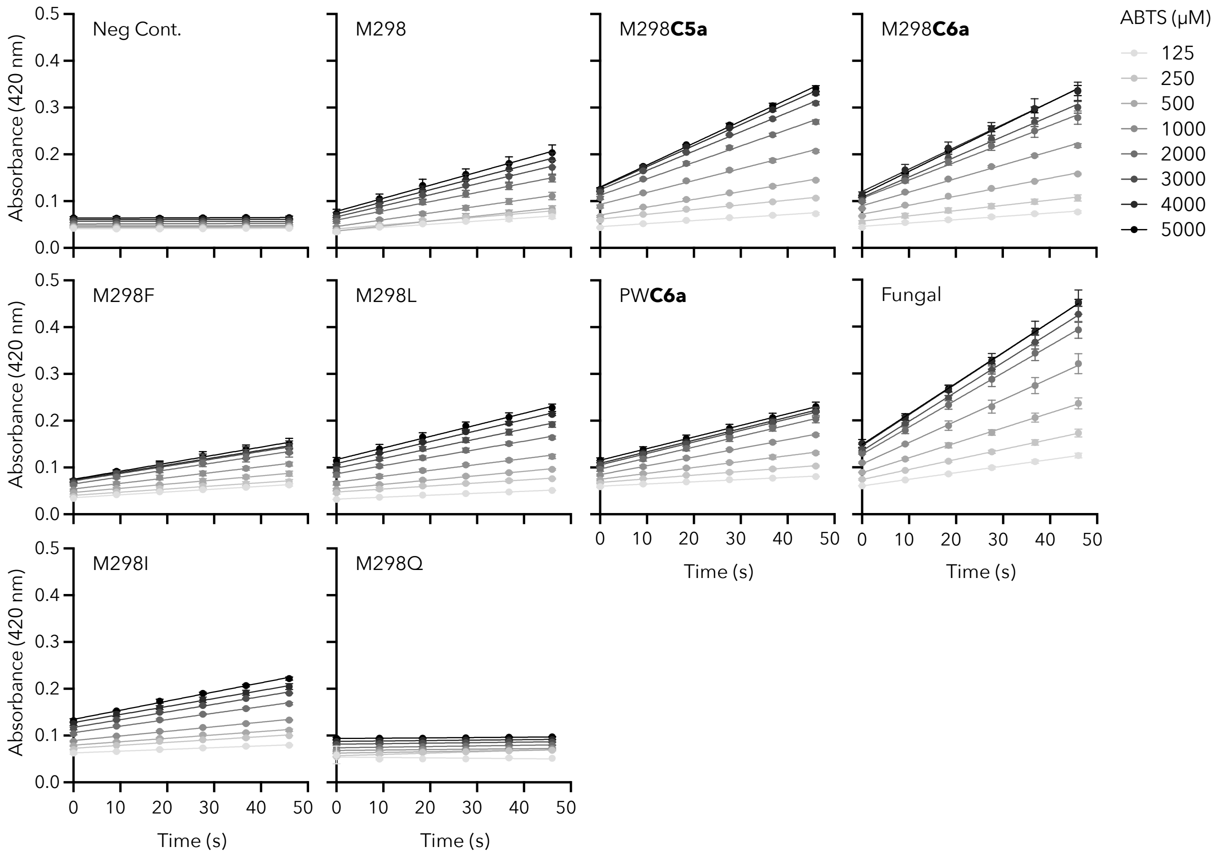

Supplementary Figure 7 | Initial velocity data for kinetics analysis with ABTS. The reactions were conducted in 20 mM BR buffer at pH 4.0, 1 mM $CuCl_2$, and varying ABTS concentrations. For the fungal laccase, the enzyme concentrations were 10 nM. For M298, M298**C5a**, and M298**C6a**, the enzyme concentrations were 200 nM. For M298F, M298L, M298I, and M298Q the enzyme concentrations were 1000 nM. For M298I, M298Q, and the fungal laccase, three preparative replicates were collected, for all other enzymes, at least three biological replicates were collected. Three preparative replicates were conducted for the negative control, which omits enzyme but contains 1 mM $CuCl_2$. The symbols represent the mean, and the error bars represent the standard deviation.

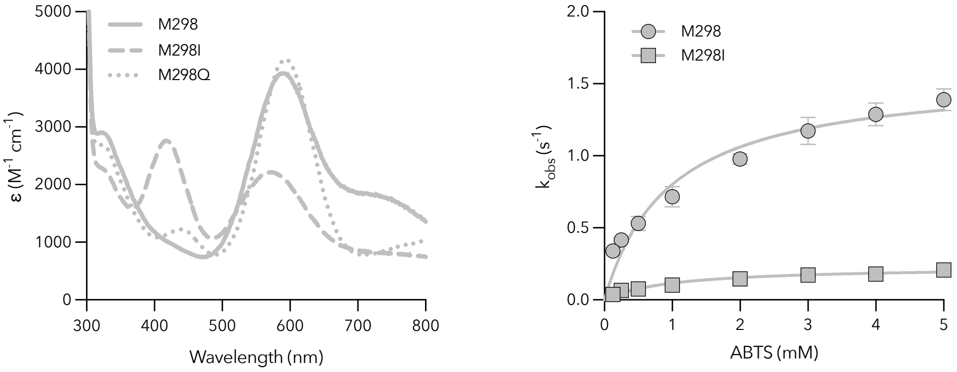

Supplementary Figure 8 | *Sc*SLAC-M298I and M298Q UV-vis spectra and Michelis-Menten kinetics of with ABTS. The reactions were conducted in 20 mM BR buffer at pH 4.0, 1 mM $CuCl_2$, and varying ABTS concentrations. The M298Q variant was not active enough to accurately estimate Michaelis-Menten kinetic parameters. M298I was measured in preparative triplicates. The data are represented as the mean and standard deviation of three replicates.

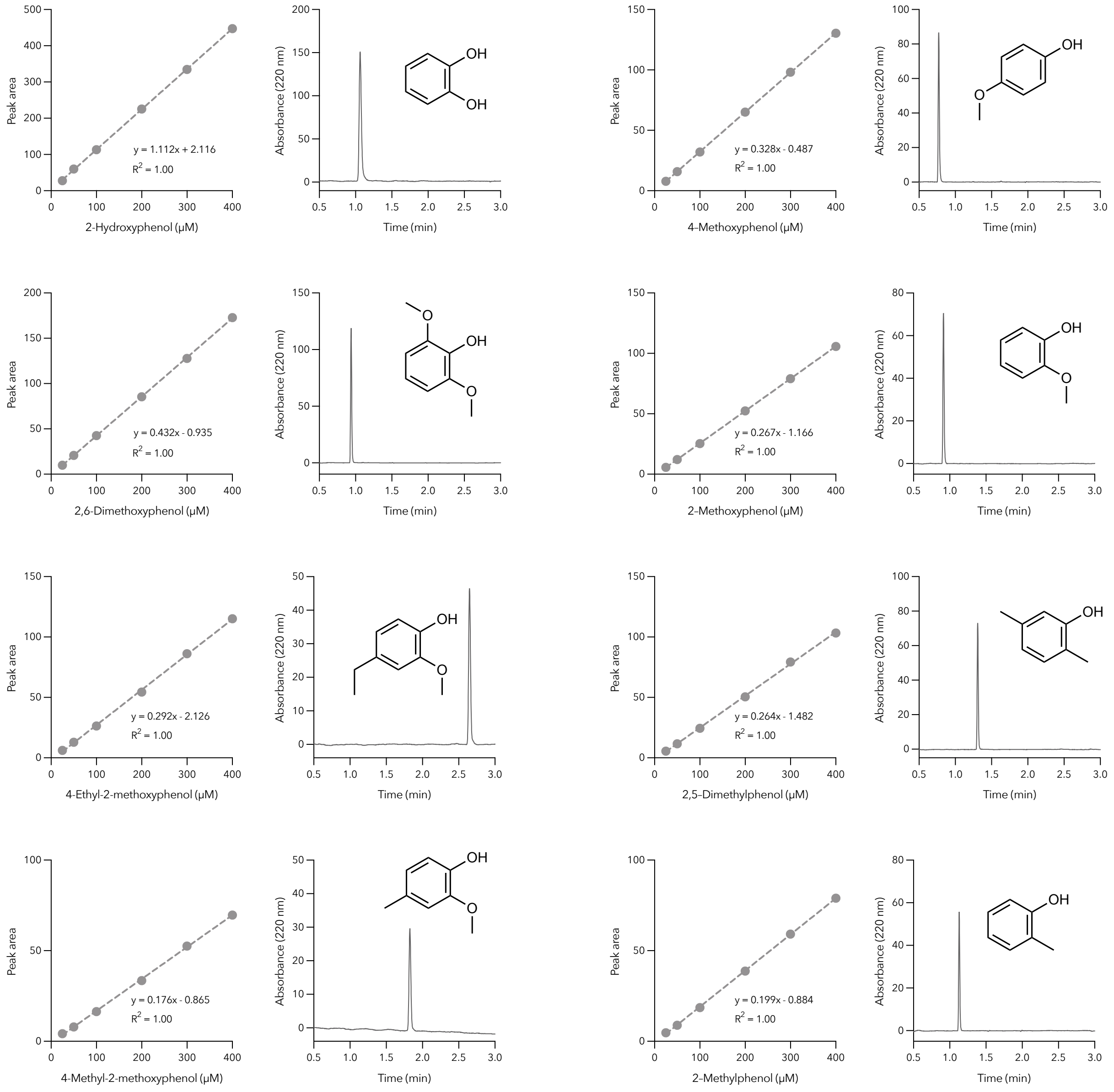

Supplementary Figure 9 | Calibration curves and representative LC-MS trace for each phenolic substrate. Calibration curves were prepared by integrating the peak area for standards at varying concentrations. All representative LC-MS data are for 300 μM phenolic substrate. All TTN experiments yielded substrate concentrations within the range of the calibration curves.

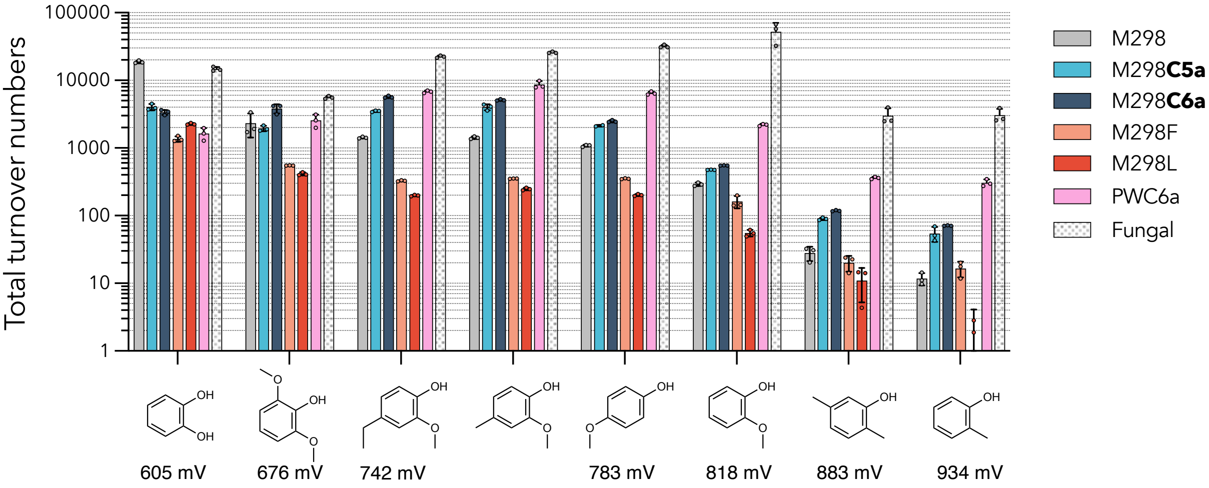

Supplementary Figure 10 | Total turnover numbers with phenolic substrates. Total turnover numbers of *Sc*SLAC M298, M298**C5a**, M298**C6a**, M298F, M298L, PW**C6a**, and the fungal laccase from *Trametes versicolor* with different phenolic substrates with their corresponding redox potential vs NHE[74]. For internal consistency, the reported redox potentials were derived from a single publication, which used a standardized method for all measurements[74]. The redox potential of 4-methyl-2-methoxyphenol was not reported by this method. Thus, the redox potential is omitted for this species. However, we posit that it is near that of 4-ethyl-2-methoxyphenol based on other reports. The total turnover was calculated based on determining the percent substrate loss over a negative control and then dividing by the enzyme concentration. To prevent full conversion and allow more accurate comparison between enzyme variants, enzyme concentrations were adjusted. Reactions were performed in biological triplicates. The data are represented as the mean and standard deviation of three replicates.

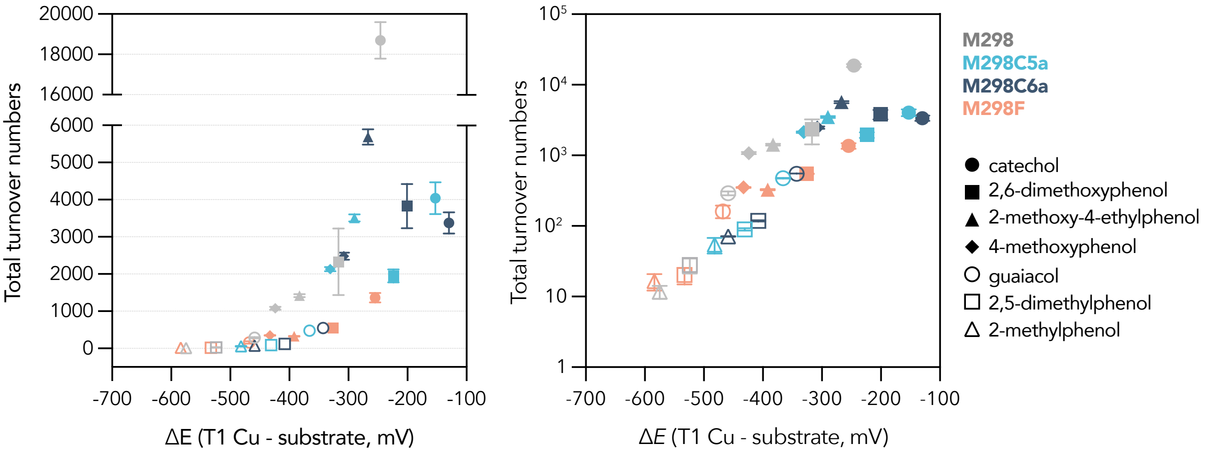

Supplementary Figure 11 | Total turnover numbers vs $E°'_{T1Cu}$ difference of *Sc*SLAC variant and phenolic substrate. The total turnover numbers of the corresponding *Sc*SLAC variant with the phenolic substrate plotted against the redox potential difference of the *Sc*SLAC variant and the respective phenolic substrate. Filled circles, catechol; filled squares, 2,6-dimethoxyphenol, filled triangle, 2-methoxy-4-ethylphenol; filled diamond, 4-methoxyphenol; open circle, guaiacol; open square, 2,5-dimethylphenol; open triangle, 2-methylphenol. The data for M298L and PW**C6a** are not shown because the redox potential could not be determined accurately. The data are illustrated as the mean and standard deviation of three biological replicates.

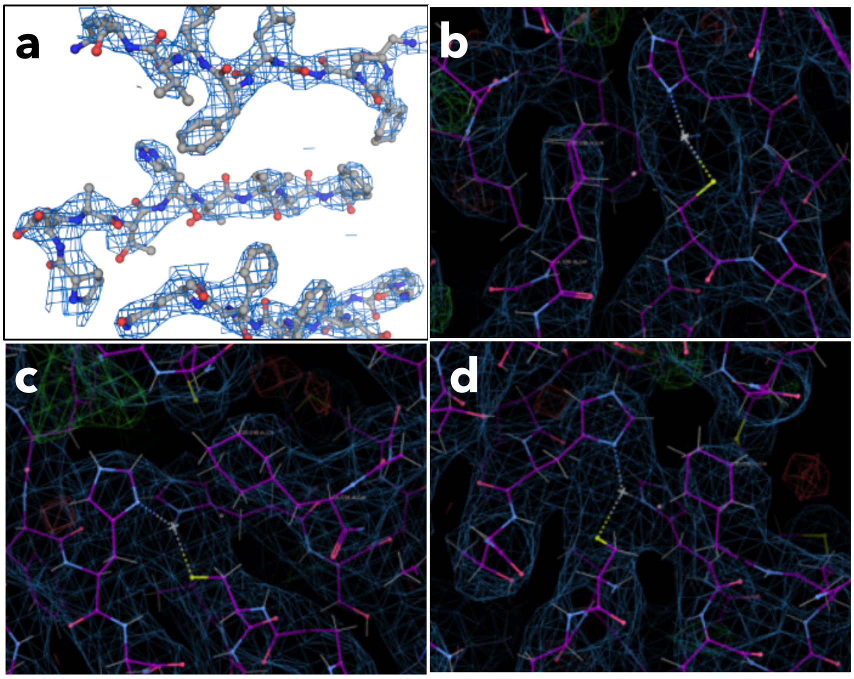

Supplementary Figure 12 | Crystallographic supplements. a) The 2Fo-Fc map for *Sc*SLAC M298**C6a** (PDB: 9HU7) illustrating the map quality, contoured at $\sigma = 1.0$. b-d) Different map angles illustrating the map quality.

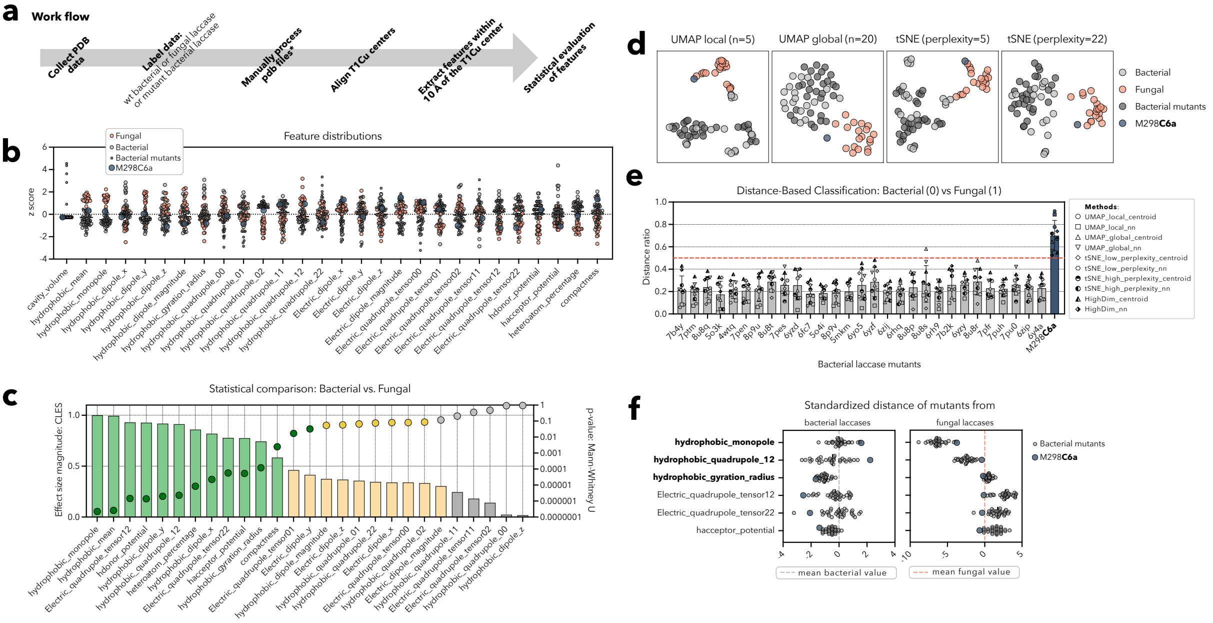

Supplementary Figure 13 | Comparison of 10 Å around the T1Cu centers of wt bacterial laccases, fungal laccases, and mutant bacterial laccases. a) Work-flow summary for the analysis. The PDB codes for the laccases analyzed are listed in Supplementary Table 7. b) Min-max normalized feature comparison. The cavity volume for all wt bacterial and fungal laccases is zero. Only axial mutant bacterial laccases contained cavities within 4 Å of the T1Cu. c) Statistical analysis of features differences between bacterial and fungal laccases. Color coding indicates features that are the most significant as green, weakly significant a yellow, and not significant as grey. Hydrophobicity features are the most statistically significant with the highest effect sizes and comprise more than 40% of the significant features. d) UMAP and t-SNE analysis of laccases for statistically significant features. e) Distance analysis comparing bacterial mutants to wt bacterial and fungal laccases. M298**C6a** is the most different from bacterial laccases and the most similar to fungal laccases. f) Illustration of the primary features contributing the difference between M298**C6a** and bacterial laccases, highlighting the importance of hydrophobicity in making M298**C6a** look less bacterial and more fungal. Importantly, ordered water molecules could not be evaluated in this model owing to differences in refinement and resolution between structures, so in practice, the decreased hydrophobicity from ordered water molecules that accumulates in the classical axial variants is not accounted for in these predictions. If it was accounted for, these variants would likely look even less fungal-like. Interestingly, the UMAP and t-SNE analysis was also able to separate the fungal T1Cu sites from Ascomycota and Basidiomycota, the latter of which are typically higher redox potential and more active than the former.

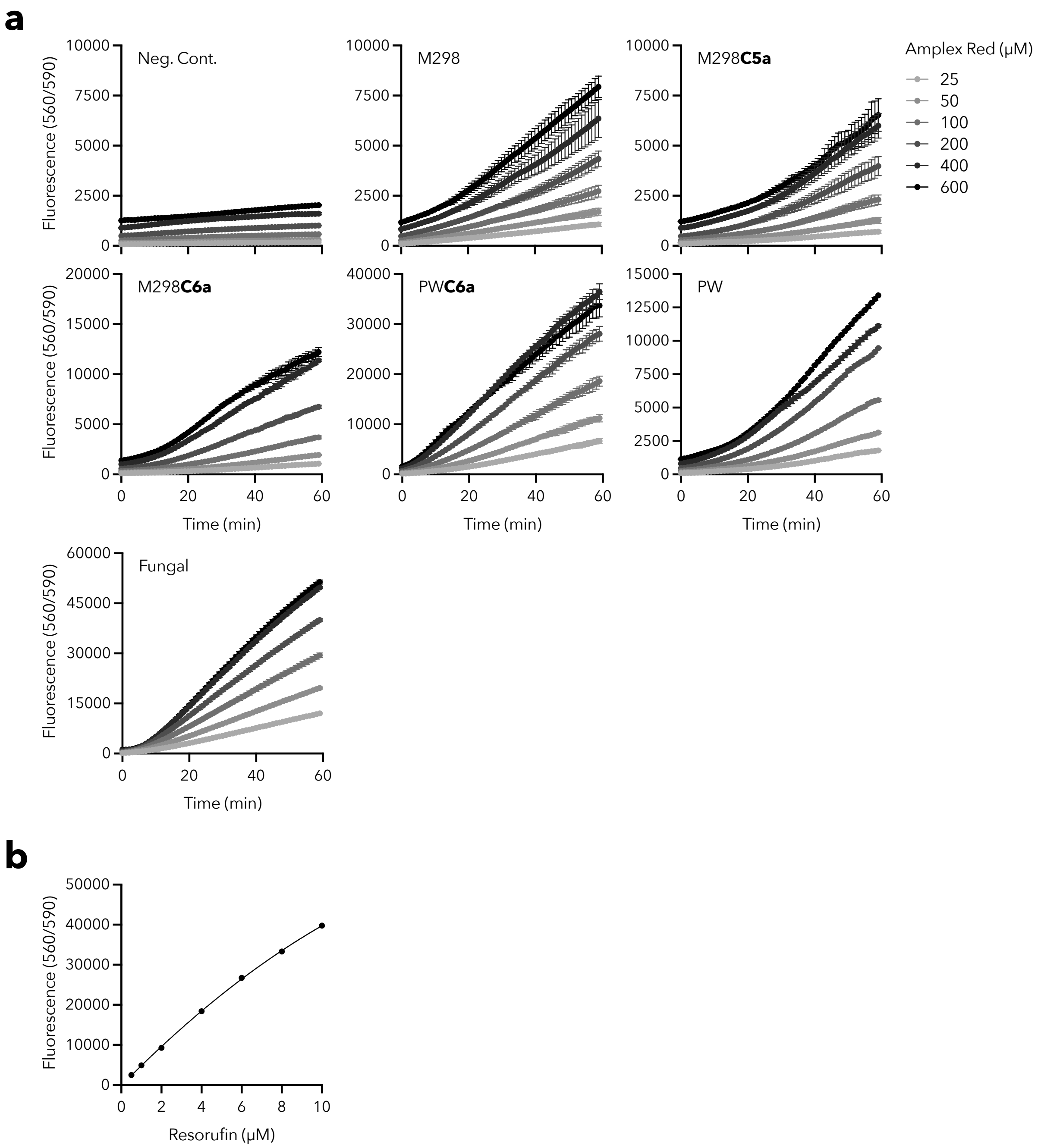

Supplementary Figure 14 | Initial velocity data and calibration curve for kinetics analysis with Amplex Red. a) The reactions were conducted in 20 mM BR buffer at pH 7.0, 0.5 mM $CuCl_2$, and varying Amplex Red concentrations. For the fungal laccase, the enzyme concentrations were 50 nM. For M298**C5a**, M298**C6a**, PW**C6a**, and PW, the enzyme concentrations were 200 nM. For M298, the enzyme concentrations were 1000 nM. For the fungal laccase, three preparative replicates were collected, for all other enzymes, three biological replicates were collected. Three preparative replicates were conducted for the negative control, which omits enzyme but contains 0.5 mM $CuCl_2$. The symbols represent the mean, and the error bars represent the standard deviation. b) Calibration curve used for the calculation of the product substrate in the kinetic analysis with Amplex Red. A polynomial fit second order was used to fit the calibration curve.

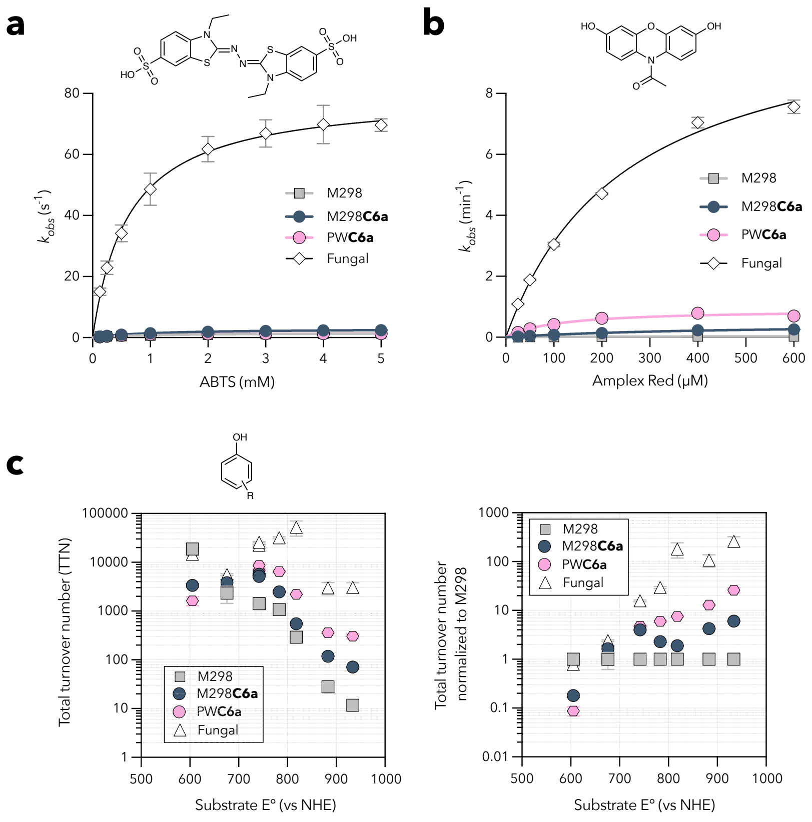

Supplementary Figure 15 | Activity comparison with fungal laccase. Michaelis-Menten kinetics of M298, M298**C6a**, PW**C6a**, and the fungal laccase from *Trametes versicolor* with ABTS as a substrate at pH 4.0 at 25 °C (a) and Amplex Red as a substrate at pH 7.0 at 25 °C (b). TTNs of M298, M298**C6a**, PW**C6a**, and the fungal laccase from *Trametes versicolor* with the phenol substrate (c). The TTNs are normalized to the TTN of ScSLAC M298 (d). Absolute values are shown in Supplementary Figure 10. The data illustrated represent the mean a standard deviation of biological triplicates for M298, M298**C6a**, and PW**C6a**, and preparative triplicates for the fungal laccase (Merck).

II. Synthesis

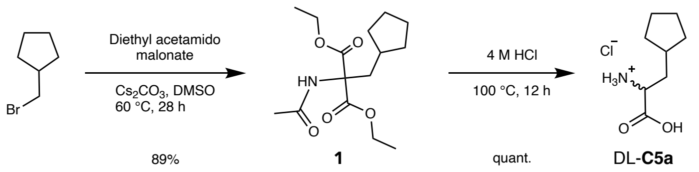

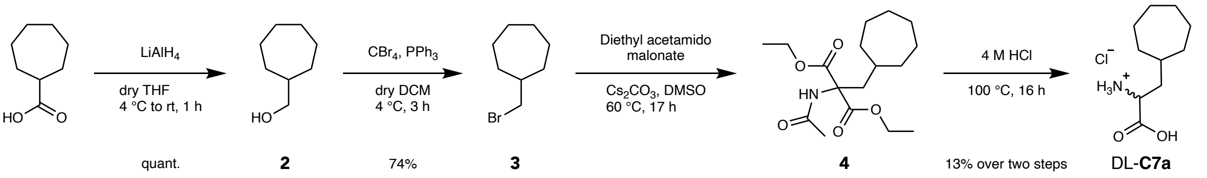

Diethyl 2-acetamido-2-(cyclopentylmethyl)malonate (1)[117]

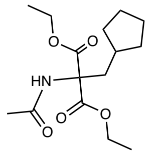

Diethyl acetamido malonate (769 mg, 3.54 mmol, 1.0 eq) was dissolved in DMSO (4 mL) under a nitrogen atmosphere. Cs_2CO_3 (1.73 g, 5.31 mmol, 1.5 eq.) was added, followed by the addition of cyclopentylmethylbromide (500 µL, 3.89 mmol, 1.1 eq.). The suspension was stirred at 60 °C for 28 h. The suspension was allowed to reach room temperature, and ice-cold water (50 mL) was added. The mixture was extracted with EtOAc (2 x 50 mL), and the combined organic phases were washed with brine (80 mL). The organic phase was dried over Na_2SO_4, and the supernatant was collected by filtration. The solvent was removed under reduced pressure to yield **1** (943 mg, 3.15 mmol, 89%) as a white solid.

^{1}H NMR (400 MHz, $CDCl_3$) δ 6.81 (s, 1H), 4.27 – 4.18 (m, 4H), 2.44 (d, $^3J_{HH}$ = 6.6 Hz, 2H), 2.03 (s, 3H), 1.76 – 1.38 (m, 7H), 1.25 (t, $^3J_{HH}$ = 7.1 Hz, 6H), 1.12 – 0.99 (m, 2H).

Cyclopentylalanine hydrochloride (C5a)

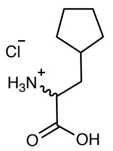

1 (943 mg, 3.15 mmol, 1.0 eq.) was suspended in aq. HCl (4 M, 20 mL) and stirred at 100 °C for 12 h. The solvent was removed under reduced pressure. The resulting white solid was dissolved in water, and the solution was lyophilized to yield a racemic mixture of **C5a** (610 mg, 3.15 mmol, quant.) as a white powder.

^{1}H NMR (400 MHz, D_2O) δ 4.03 (t, $^3J_{HH}$ = 6.6 Hz, 1H), 2.08 – 1.78 (m, 5H), 1.73 – 1.50 (m, 4H), 1.26 – 1.11 (m, 2H).
^{13}C NMR (101 MHz, D_2O) δ 172.9, 52.7, 36.1, 35.5, 32.0, 31.7, 24.6, 24.4.

MS (ESI): calc. for $[M+H]^+$: 158.12, obs.: 158.2.

Cyclohepthylmethanol (2)

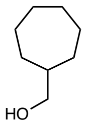

Cycloheptanecarboxylic acid (1.01 g, 7.03 mmol, 1.0 eq.) was dissolved in dry THF (20 mL) under a nitrogen atmosphere, and the solution was cooled to 0 °C. $LiAlH_4$ (2.4 M in THF, 8.8 mL, 21 mmol, 3.0 eq.) was added dropwise, and the reaction was stirred at 0 °C for 1 h, followed by 1.5 h at room temperature. The reaction mixture was cooled to 0 °C, and water (2 mL), aq. NaOH (15%, 2 mL), and water (4 mL) were added. The slurry was stirred for 10 min and filtered through Celite. The filter cake was washed with EtOAc (3x), and the combined organic phases were washed with water and brine. The organic phase was dried over Na_2SO_4, and the supernatant was collected by filtration. The solvent was removed under reduced pressure to yield **2** (900 mg, 7.03 mmol, quant.) as a colorless oil.

^{1}H NMR (400 MHz, $CDCl_3$) δ 3.42 (d, $^3J_{HH}$ = 6.6 Hz, 2H), 1.80 – 1.38 (m, 11H), 1.25 – 1.13 (m, 2H).

Cyclohepthylmethylbromide (3)

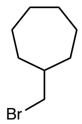

2 (900 mg, 7.03 mmol, 1.0 eq.) and CBr_4 (3.04 g, 9.17 mmol, 1.3 eq.) were dissolved in dry DCM (13 mL) under a nitrogen atmosphere, and the solution was cooled to 0 °C. A solution of PPh_3 (2.41 g, 9.17 mmol, 1.3 eq.) in dry DCM (13 mL) was added dropwise, and the mixture was stirred at 4 °C for 3 h. The solvent was evaporated under reduced pressure, n-pentane (30 mL) was added, and the suspension was sonicated for 5 min. The flask was chilled in a freezer for 30 min, and the suspension was filtered. The precipitate was washed with chilled n-pentane, and the precipitation was once more worked up by a cycle of suspension, sonication, chilling, and filtering. Both filtrates were combined, the solvent was removed under reduced pressure to yield **3** as a yellowish oil (1.08 g, 5.68 mmol, 74%). There was still triphenylphosphine oxide present, but it was used without further purification for the next step.

^{1}H NMR (400 MHz, $CDCl_3$) δ 3.32 (d, $^3J_{HH}$ = 5.9 Hz, 2H), 1.92 – 1.22 (m, 13H). With triphenylphosphine contamination in the aromatic region.

Diethyl 2-acetamido-2-(cycloheptylmethyl)malonate (4)

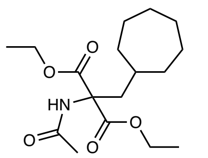

Diethyl acetamidomalonate (1.03 g, 4.76 mmol, 1.0 eq.) was dissolved in DMSO (2 mL) under a nitrogen atmosphere. Cs_2CO_3 (2.33 g, 7.14 g, 1.5 eq.) and **3** (1.00 g, 5.23 mmol, 1.1 eq.) in DMSO (3 mL) were added and the suspension was stirred at 60 °C for 17 h. The reaction was allowed to reach room temperature, and ice-cold water (60 mL) was added. The mixture was extracted with EtOAc (2 x 60 mL), and the combined organic phases were washed with brine (100 mL). The organic phase was dried over Na_2SO_4, and the supernatant was collected by filtration. The solvent was removed under reduced pressure to give an orange oil. The crude product was purified by automated flash column chromatography (25 g Si_2O, 15% EtOAc in cyclohexane to 100% EtOAc) to yield an orange solid. Although the product still contained minor impurities, the desired compound peaks could be identified, and the crude material was taken for the next step.

^{1}H NMR (400 MHz, $CDCl_3$) δ 4.23 (q, $^3J_{HH}$ = 7.1 Hz, 4H), 2.33 (d, $^3J_{HH}$ = 6.0 Hz, 2H), 2.03 (s, 3H), 1.65 – 1.16 (m, 13H), 1.25 (t, $^3J_{HH}$ = 7.1 Hz, 6H).

Cycloheptylalanine hydrochloride (C7a)

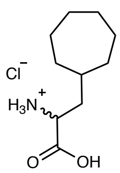

Crude **4** (approx. 570 mg, 1.74 mmol, 1.0 eq.) was dissolved in HCl (4 M in dioxane, 10 mL) and stirred at 100 °C for 16 h. Starting material was still observed by LC-MS. Thus, additional aq. HCl (4 M, 10 mL) was added. The reaction mixture was stirred at 100 °C for another 24 h, and the solvents were evaporated under reduced pressure to yield **C7a** (145 mg, 654 µmol, 13% over 2 steps) as a brownish solid.

^{1}H NMR (400 MHz, D_2O) δ 4.01 (dd, $^3J_{HH}$ = 8.4, 5.8 Hz, 1H), 1.96 – 1.86 (m, 1H), 1.80 – 1.38 (m, 12H), 1.33 – 1.20 (m, 2H).

^{13}C NMR (101 MHz, D_2O) δ 173.3, 51.7, 38.2, 34.6, 34.0, 32.9, 27.9, 27.9, 25.5, 25.3.

MS (ESI): calc. for $[M+H]^+$: 186.15, obs.: 186.2.

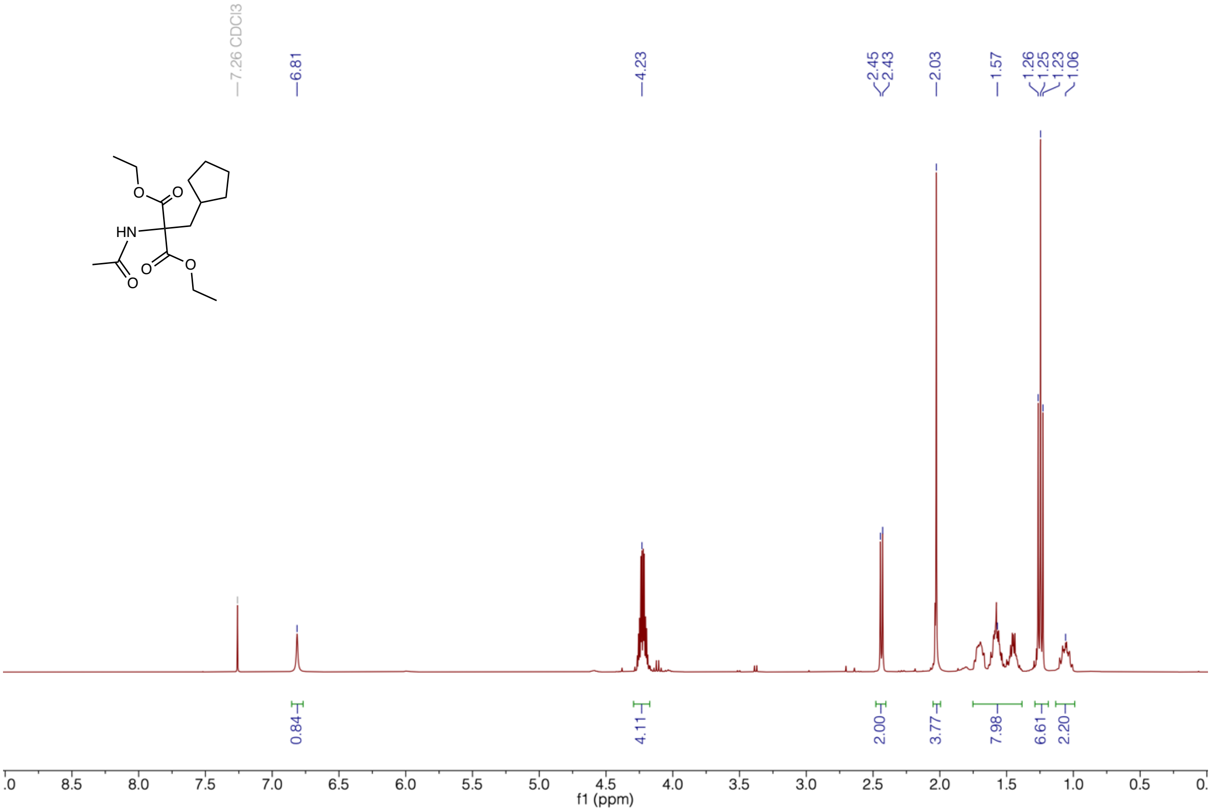

Supplementary Figure 16 | ^{1}H-NMR of 1.

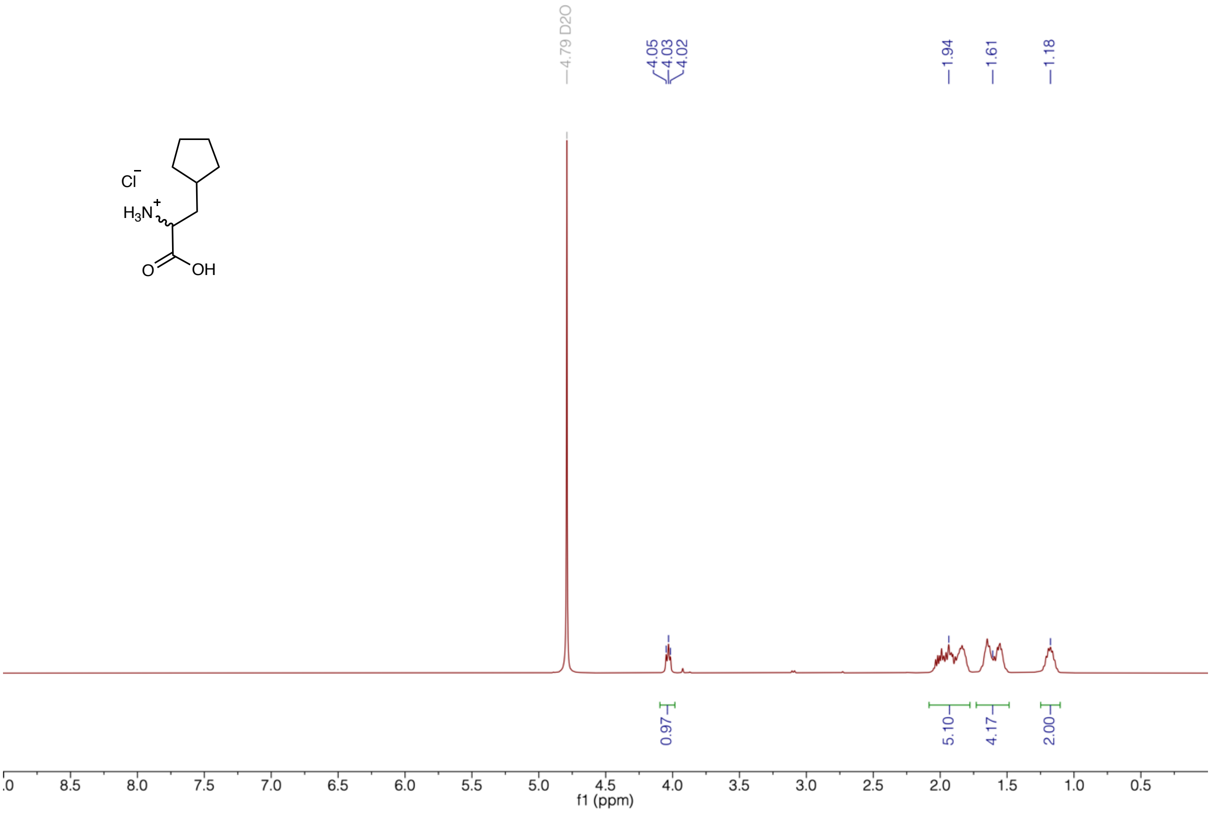

Supplementary Figure 17 | ^{1}H-NMR of C5a.

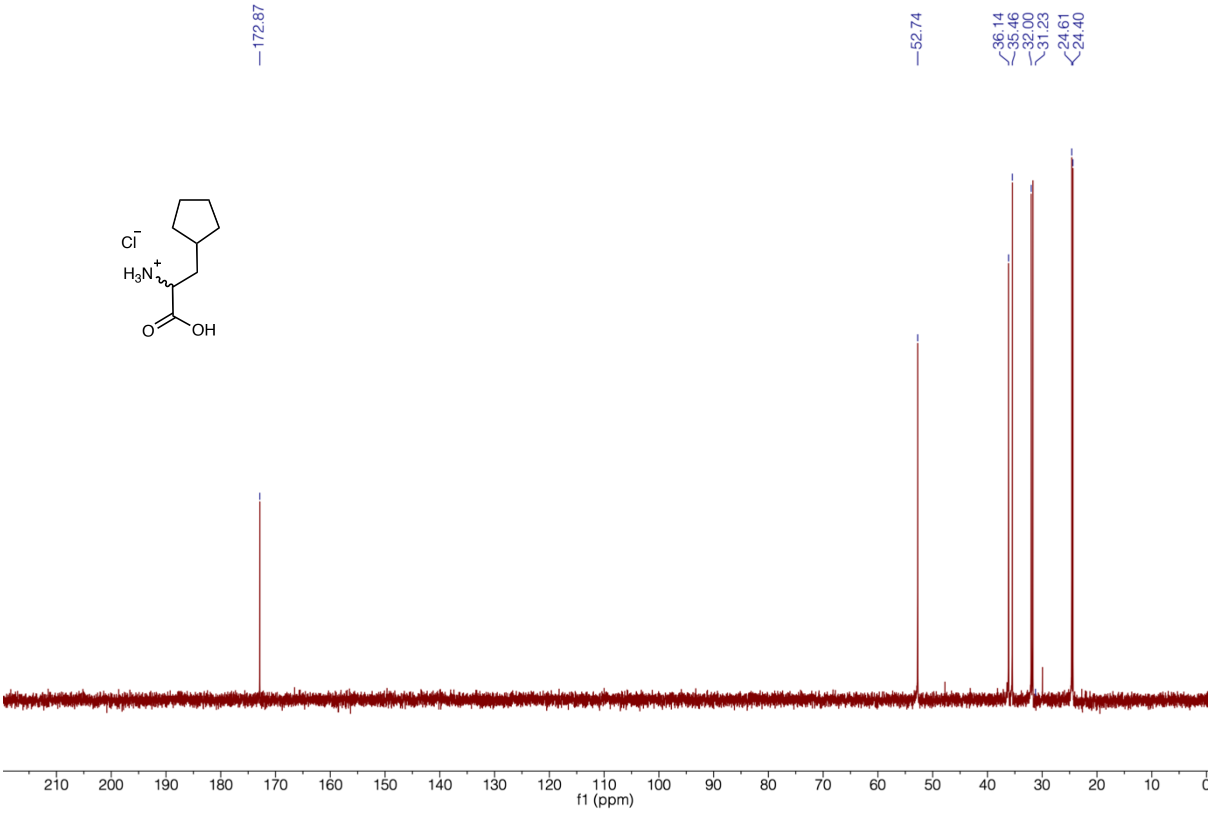

Supplementary Figure 18 | ^{13}C-NMR of C5a.

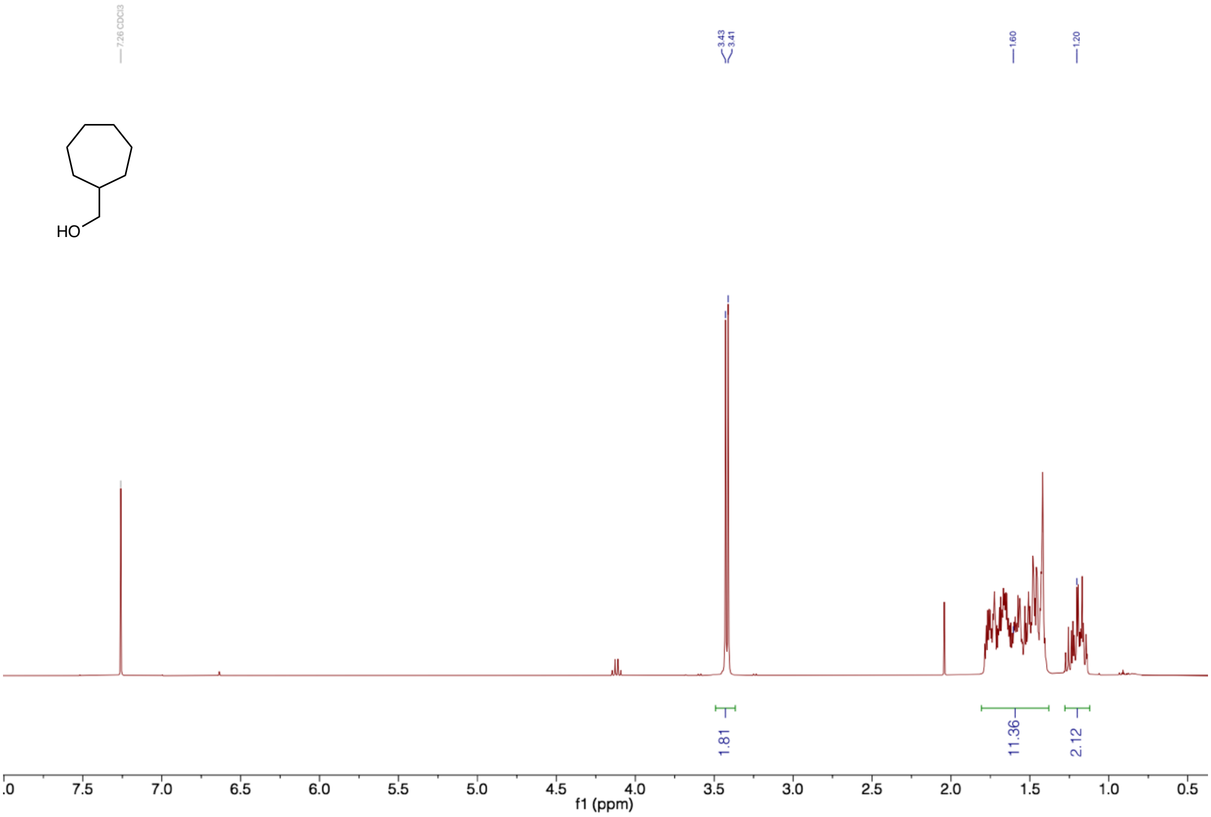

Supplementary Figure 19 | ¹H-NMR of 2.

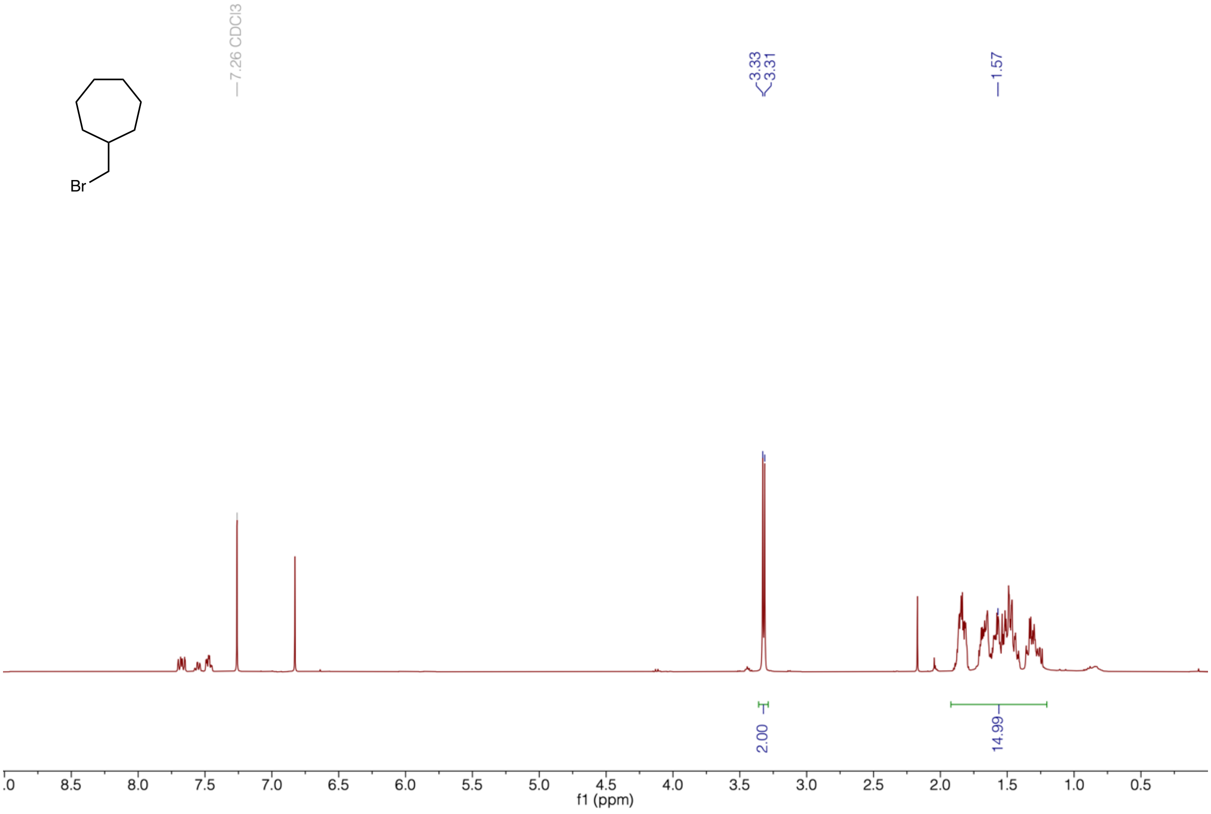

Supplementary Figure 20 | ¹H-NMR of crude 3.

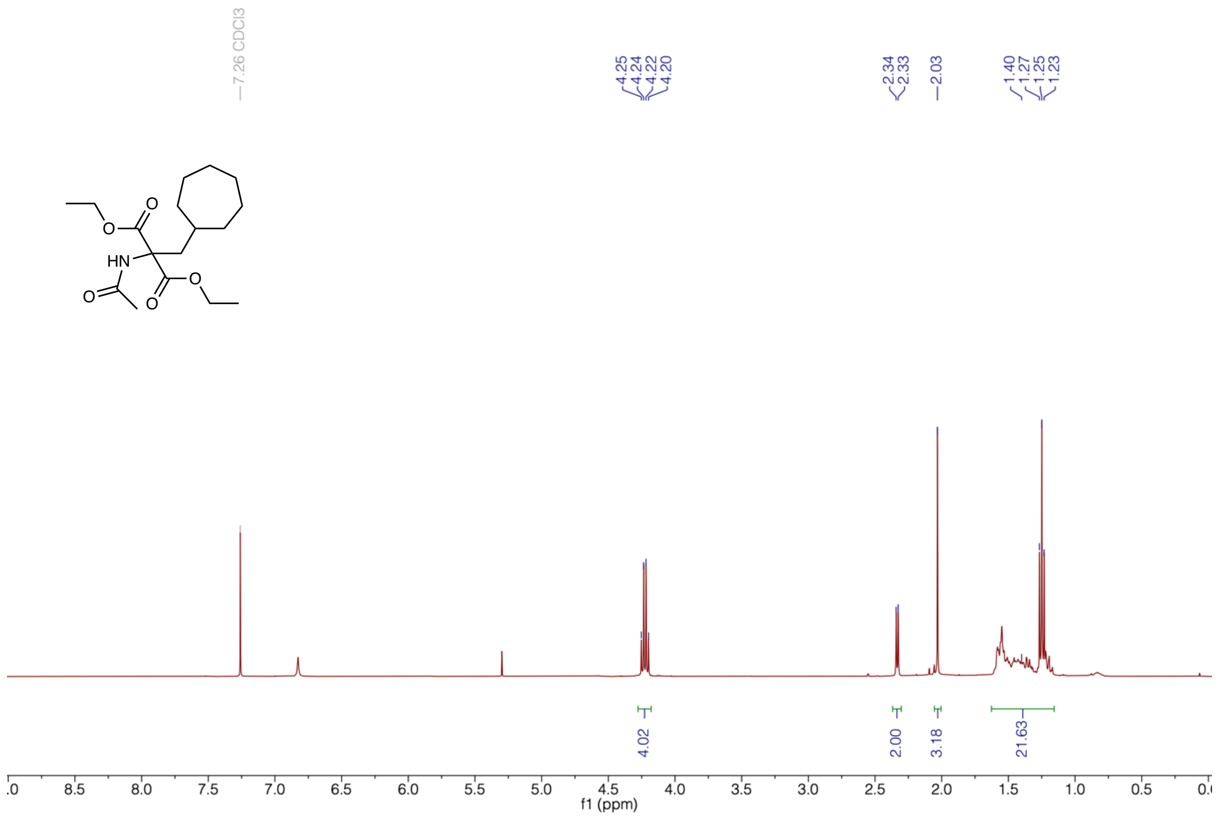

Supplementary Figure 21 | ^{1}H-NMR of 4.

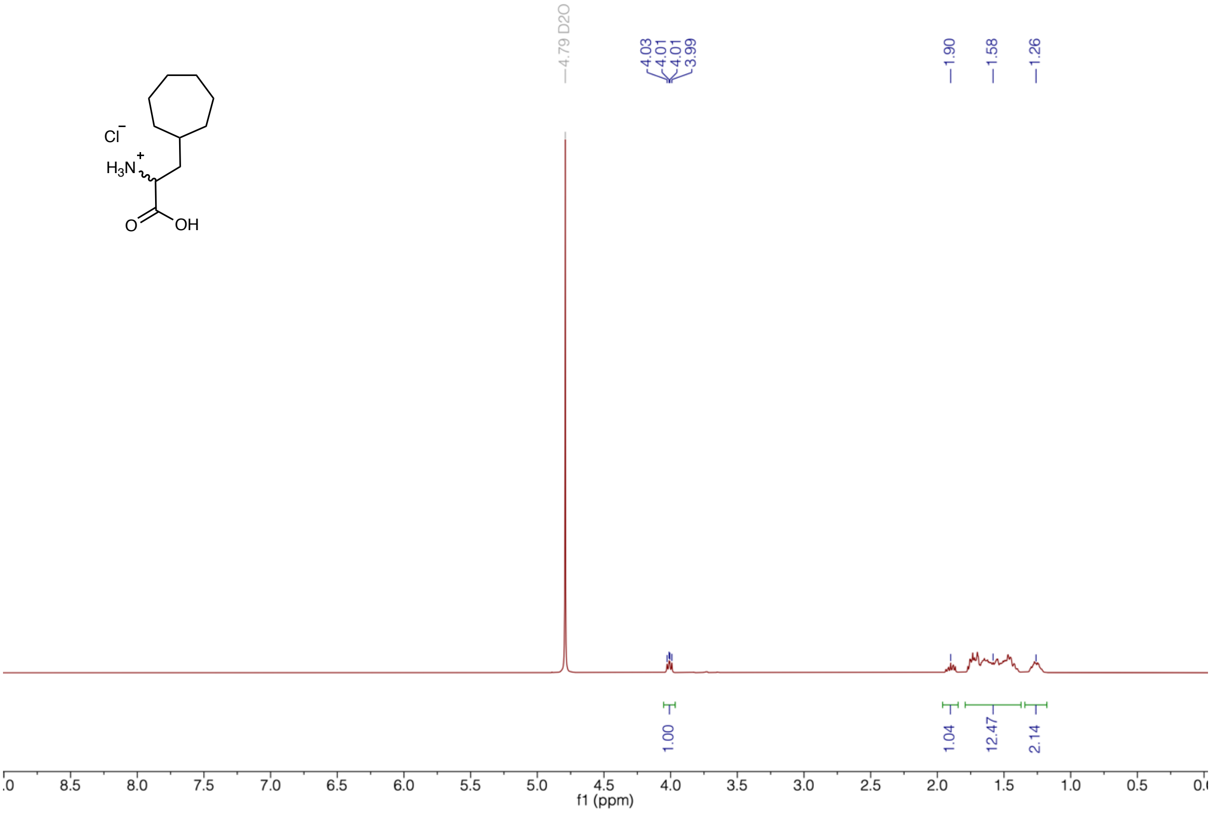

Supplementary Figure 22 | ¹H-NMR of C7a.

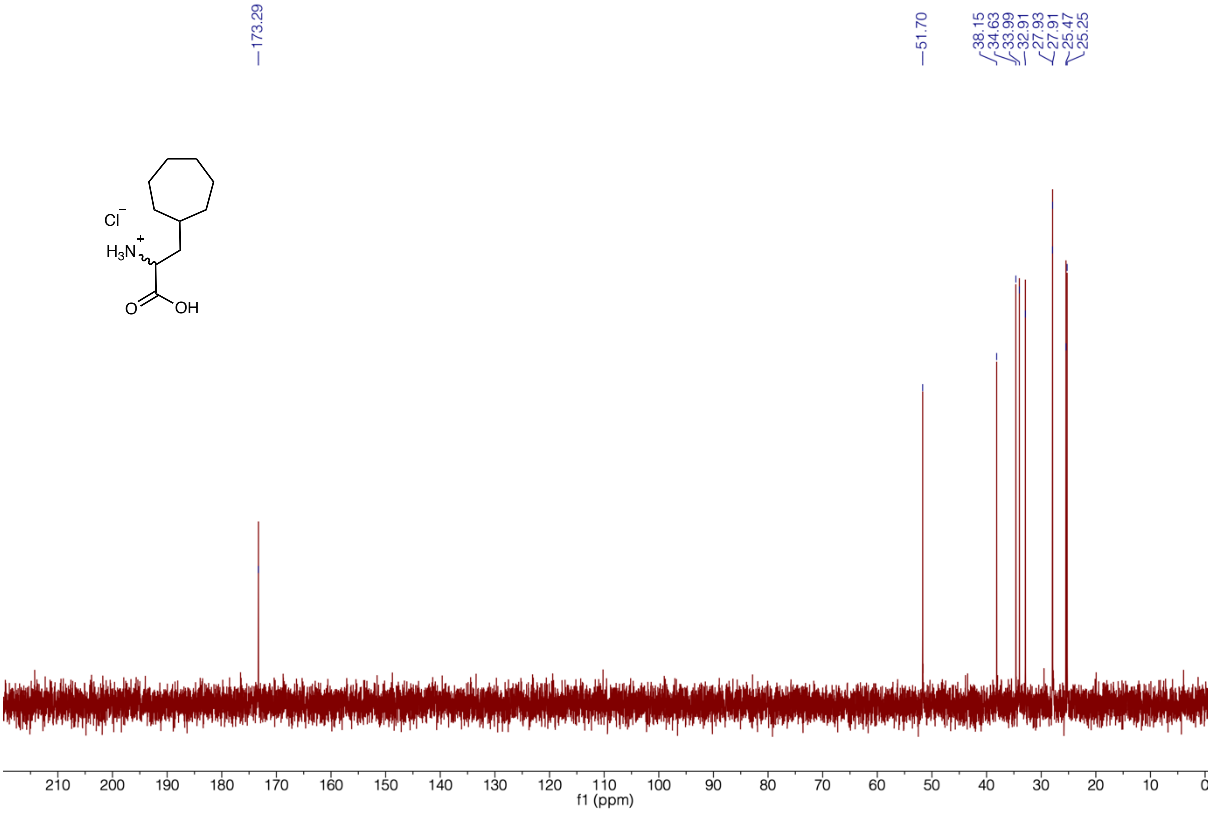

Supplementary Figure 23 | ¹³C-NMR of C7a.

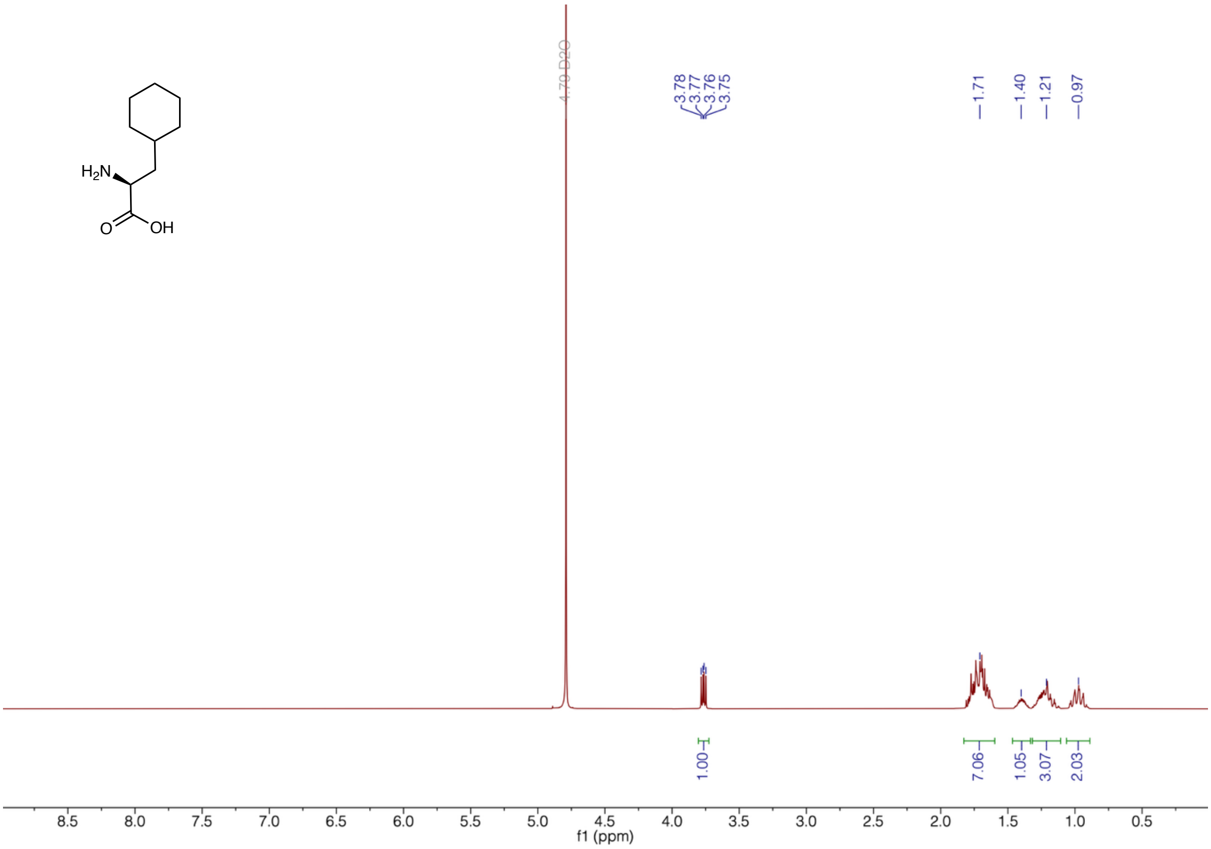

Supplementary Figure 24 | ^{1}H-NMR of commercially available C6a.

III. Supplementary Data Tables

Supplementary Table 1 | Redox potentials and UV-vis parameters of *Sc*SLAC variants.

Variant	$E°'_{T1Cu}$ (mV)	$\Delta E°'_{T1Cu}$ (mV)	ε_σ (λ)	ε_π (λ)	$\varepsilon_{\sigma/\pi}$*	R' ($\varepsilon_\sigma/(\varepsilon_\sigma + \varepsilon_\pi)$)
M298	366(7)	--	--	3927 (590)	0.3	0.00
M298**C5a**	457(3)	91	1719 (425)	1752 (578)	0.98	0.50
M298**C6a**	476(4)	110	1503 (423)	2000 (581)	0.75	0.43
M298F	346(11)	-20	1680 (439)	2744 (591)	0.61	0.38
M298L	<350	--	2075 (420)	1624 (565)	1.28	0.56
PW**C6a**	>475	--	573 (420)	1264 (595)	0.46	0.31
Fungal‡	n.d.	--	--	--	--	--
M298I	n.d.	--	2761 (417)	2214 (572)	1.22	0.55
M298Q	n.d.	--	1225 (438)	4175 (594)	0.29	0.23

n.d. = not determined. Extinction coefficients are reported in $M^{-1}cm^{-1}$, and wavelengths are reported in nm. *The relative intensities of the UV-vis bands at ~450 nm and ~600 nm ($\varepsilon_{\sigma/\pi}$) have been connected to the extent of tetragonal distortion from a pseudo-tetrahedral His_2CysMet T1Cu or exchange of the coordinating ligands[32]. Surprisingly, a continuum of relative intensities exists even for sites with the same coordination motifs. Such that both "blue" (low $\varepsilon_{\sigma/\pi}$) and "green" (high $\varepsilon_{\sigma/\pi}$) copper proteins can arise from the canonical His_2CysMet T1Cu motif[32]. ‡Fungal laccase from *Trametes versicolor* (Merck).

Supplementary Table 2 | EPR parameters of *Sc*SLAC variants.

	parameter	wtSLAC	M298**C5a**	M298**C6a**	M298F	M298L	PW**C6a**
**T1Cu**	$g_\perp$	2.039	2.056	2.055	2.067	2.060	2.046
	$g_\parallel$	2.228	2.263	2.261	2.292	2.260	2.260
	$A_\perp$ (x 10^4 cm^{-1})	9.0	24	25	21	30	21
	$A_\parallel$ (x 10^4 cm^{-1})	75	81	79	52	82	85
	lw	4.0	5.6	4.8	9.6	4.8	5.5
	fraction	0.50	0.50	0.50	0.50	0.50	0.50
**T2Cu**	$g_\perp$	2.050	2.049	2.049	2.050	2.050	2.049
	$g_\parallel$	2.246	2.248	2.248	2.248	2.246	2.248
	$A_\perp$ (x 10^4 cm^{-1})	9.3	9.3	9.5	9.5	9.5	9.3
	$A_\parallel$ (x 10^4 cm^{-1})	195	197	193	194	197	197
	lw	5.9	6.2	5.8	6.4	5.5	6.0
	fraction	0.50	0.50	0.50	0.50	0.50	0.50

Supplementary Table 3 | Kinetic parameters for *Sc*SLAC variants with ABTS as a substrate.

Variant	k_{cat} (s^{-1})	K_M (mM)	k_{cat}/K_M (s^{-1} mM^{-1})
M298	1.6(1)	0.96(5)	1.6(2)
M298**C5a**	3.1(1)	1.27(2)	2.5(1)
M298**C6a**	3.0(2)	1.05(5)	2.8(1)
M298F	0.19(3)	0.4(1)	0.46(4)
M298L	0.32(1)	1.19(7)	0.27(1)
PW**C6a**	1.5(1)	0.58(4)	2.6(2)
Fungal	80(4)	0.63(5)	128(17)
M298I	0.23(2)	1.0(2)	0.23(2)
M298Q	n.d.	n.d.	n.d.

Supplementary Table 4 | Kinetic parameters for *Sc*SLAC variants with Amplex Red as a substrate.

Variant	k_{cat} (min^{-1})	K_M (mM)	k_{cat}/K_M (min^{-1} mM^{-1})
M298	0.045(4)	0.25(3)	0.18(3)
M298**C5a**	0.23(8)	0.3(1)	0.70(3)
M298**C6a**	0.45(1)	0.44(1)	1.04(3)
PW**C6a**	0.91(7)	0.11(1)	8.4(4)
Fungal	11.0(3)	0.25(1)	44(1)
PW	0.405(4)	0.144(5)	2.81(8)

Supplementary Table 5 | Analysis of evolutionary improvements.

Substrate	E (mV)	Parameter	Improvements upon engineering		
			C6a vs. wt	PWC6a vs. M298C6a	PWC6a vs. wt
Amplex Red	1140	k_{cat}	10	2.0	20
		k_{cat}/K_M	5.8	8.1	47
Catechol	605	TTN	0.18	0.48	0.09
2,6-DMP	676	TTN	1.6	0.67	1.1
4Et2MeOP	742	TTN	4.0	1.2	4.8
4MeOP	783	TTN	2.3	2.6	6.0
2MeOP	818	TTN	1.9	4.0	7.6
2,5-DMP	883	TTN	4.2	3.0	13
Cresol	934	TTN	6.0	4.3	26
ABTS	680	k_{cat}	1.9	0.50	0.94
		k_{cat}/K_M	1.8	0.93	1.6

2,6-DMP = 2,6-dimethoxyphenol, 4Et2MeOP = 4-ethyl-2-methoxyphenol, 4MeOP = 4-methoxyphenol, 2MeOP = 2-methoxyphenol, and 2,5-DMP = 2,5-dimethylphenol.

Supplementary Table 6 | Overview of PylRS libraries used in this study.

Library	Fixed residues	Randomized residues
**1**	I405R	M300, A302, N346, C348
1.1	-	L305, L309, N346, C348, W417
1.2	-	M300, A302, N346, C348, W417
1.3	-	L305, L309, N346, C348, V401
**2**	M300D, A302H, N346A, I405R	S399, A400, V401
2.1	M300D, A302H, N346A, I405R	W417, G419

Supplementary Table 7 | PDB codes for computational analysis.

pdb_file	Type	Note
1gw0	Fungal	Melanocarpus albomyces
3t6v	Fungal	Steccherinum ochraceum
5ehf	Fungal	Antrodiella faginea
2h5u	Fungal	Laccase, Cerrena maxima
2hrh	Fungal	Trametes trogii
1v10	Fungal	Laccase, Rigidoporus lignosus
2xyb	Fungal	Pycnoporus cinnabarinus
2hrg	Fungal	Laccase, Trametes trogii
5e9n	Fungal	Steccherinum murashkinskyi
4a2e	Fungal	Coriolopsis gallica
3pps	Fungal	Thielavia arenaria
6h5y	Fungal	Aspergillus oryzae
2qt6	Fungal	Lentinus Tigrinus
2hzh	Fungal	Coriolus zonatus
3sqr	Fungal	Botrytis aclada
5lm8	Fungal	Aspergillus niger
3fpx	Fungal	Trametes hirsuta
3kw7	Fungal	Trametes sp
1gyc	Fungal	Trametes versicolor
4gxf	Bacterial	SLAC, Streptomyces coelicolor
3tas	Bacterial	SLAC, Streptomyces viridosporus
3kw8	Bacterial	SLAC, Streptomyces coelicolor
8z59	Bacterial	Sulfurimonas sp
3ta4	Bacterial	Laccase, Amycolatopsis sp.
5lhl	Bacterial	SLAC, Streptomyces griseoflavus
6xj0	Bacterial	Pediococcus pentosaceus
9bd5	Bacterial	Bacillus licheniformis
9f1t	Bacterial	Oenococcus oeni
4m3h	Bacterial	Ssl1, Streptomyces sviceus
1n68	Bacterial	CueO, Escherichia coli

7bdn	Bacterial	SLAC, Streptomyces coelicolor
4q89	Bacterial	CotA, Escherichia coli
3t9w	Bacterial	SLAC, Amycolatopsis sp
6q29	Bacterial	Thermus thermophilus
6tyr	Bacterial	Thermus thermophilus
8z5b	Bacterial	Bacillus freudenreichii
6ttd	Bacterial	Aquifex aeolicus
5o3k	Mutant	SLAC_M54L/M64L/M96L, Streptomyces griseoflavus
7pen	Mutant	SLAC_Y230A, Streptomyces griseoflavus
8p9u	Mutant	SLAC_M199A/D268N, Streptomyces griseoflavus
7pfr	Mutant	SLAC_M199A, Streptomyces griseoflavus
8p9v	Mutant	SLAC_M199G/R240H/D268N, Streptomyces griseoflavus
8u8q	Mutant	SLAC_V290N/S292F
8u8p	Mutant	SLAC_S292F
8u8r	Mutant	SLAC_Y229F/V290N/S292F
6yzy	Mutant	Ssl1_M295V, Streptomyces sviceus
8u8s	Mutant	SLAC_Y229F/S292F
6zij	Mutant	SLAC_R240H, Streptomyces griseoflavus
8u8t	Mutant	SLAC_Y229F/V290N
6rhq	Mutant	SLAC_I170A, Streptomyces griseoflavus
5o4i	Mutant	SLAC_M54L/M64L/M96L, Streptomyces griseoflavus
7pu0	Mutant	SLAC_H165A/M199G, Streptomyces griseoflavus
6yzd	Mutant	Ssl1_M295A, Streptomyces sviceus
6zip	Mutant	SLAC_R240A, Streptomyces griseoflavus
7puh	Mutant	SLAC_H165A/R240H, Streptomyces griseoflavus
6yzf	Mutant	Ssl1_M295Y, Streptomyces sviceus
4wtq	Mutant	Ssl1_M295L, Streptomyces sviceus
6fc7	Mutant	SLAC_H165F, Streptomyces griseoflavus
7ptm	Mutant	SLAC_M199G/R240H, Streptomyces griseoflavus
6yo5	Mutant	Ssl1_M295F, Streptomyces sviceus
7b2k	Mutant	SLAC_M298F, Streptomyces coelicolor
6y4a	Mutant	Ssl1_M295I, Streptomyces sviceus
6rh9	Mutant	SLAC_I170F, Streptomyces griseoflavus
5mkm	Mutant	SLAC_H165F, Streptomyces griseoflavus
7pes	Mutant	SLAC_M199G, Streptomyces griseoflavus
7b4y	Mutant	SLAC_M298L, Streptomyces coelicolor
9HU7	Mutant	SLAC_M298**C6a**, Streptomyces coelicolor

IV. DNA and Protein Sequences

DNA sequence of sfGFP150$_{TAG}$:

ATGGTTAGCAAAGGTGAAGAACTGTTTACCGGCGTTGTGCCGATTCTGGTGGAACTGGATGGTGATGTGAATGGCCATAAATT
TAGCGTTCGTGGCGAAGGCGAAGGTGATGCGACCAACGGTAAACTGACCCTGAAATTTATTTGCACCACCGGTAAACTGCCGG
TTCCGTGGCCGACCCTGGTGACCACCCTGACCTATGGCGTTCAGTGCTTTAGCCGCTATCCGGATCATATGAAACGCCATGAT
TTCTTTAAAAGCGCGATGCCGGAAGGCTATGTGCAGGAACGTACCATTAGCTTCAAAGATGATGGCACCTATAAAACCCGTGC
GGAAGTTAAATTTGAAGGCGATACCCTGGTGAACCGCATTGAACTGAAAGGTATTGATTTTAAAGAAGATGGCAACATTCTGG
GTCATAAACTGGAATATAATTTCAACAGCCATTAGGTGTATATTACCGCCGATAAACAGAAAAATGGCATCAAAGCGAACTTT
AAAATCCGTCACAACGTGGAAGATGGTAGCGTGCAGCTGGCGGATCATTATCAGCAGAATACCCCGATTGGTGATGGCCCGGT
GCTGCTGCCGGATAATCATTATCTGAGCACCCAGAGCGTTCTGAGCAAAGATCCGAATGAAAAACGTGATCATATGGTGCTGC
TGGAATTTGTTACCGCCGCGGGCATTACCCACGGTATGGATGAACTGTATAAAGGCAGCCACCATCATCATCACCATTAA

Protein sequence of sfGFP150$_{TAG}$ with (ncAA) at position 150:

MVSKGEELFTGVVPILVELDGDVNGHKFSVRGEGEGDATNGKLTLKFICTTGKLPVPWPTLVTTLTYGVQCFSRYPDHMKRHD
FFKSAMPEGYVQERTISFKDDGTYKTRAEVKFEGDTLVNRIELKGIDFKEDGNILGHKLEYNFNSH(ncAA)VYITADKQKNG
IKANFKIRHNVEDGSVQLADHYQQNTPIGDGPVLLPDNHYLSTQSVLSKDPNEKRDHMVLLEFVTAAGITHGMDELYKGSHHH
HHH*

DNA sequence of wild-type sfGFP:

ATGGTTAGCAAAGGTGAAGAACTGTTTACCGGCGTTGTGCCGATTCTGGTGGAACTGGATGGTGATGTGAATGGCCATAAATT
TAGCGTTCGTGGCGAAGGCGAAGGTGATGCGACCAACGGTAAACTGACCCTGAAATTTATTTGCACCACCGGTAAACTGCCGG
TTCCGTGGCCGACCCTGGTGACCACCCTGACCTATGGCGTTCAGTGCTTTAGCCGCTATCCGGATCATATGAAACGCCATGAT
TTCTTTAAAAGCGCGATGCCGGAAGGCTATGTGCAGGAACGTACCATTAGCTTCAAAGATGATGGCACCTATAAAACCCGTGC
GGAAGTTAAATTTGAAGGCGATACCCTGGTGAACCGCATTGAACTGAAAGGTATTGATTTTAAAGAAGATGGCAACATTCTGG
GTCATAAACTGGAATATAATTTCAACAGCCATAACGTGTATATTACCGCCGATAAACAGAAAAATGGCATCAAAGCGAACTTT
AAAATCCGTCACAACGTGGAAGATGGTAGCGTGCAGCTGGCGGATCATTATCAGCAGAATACCCCGATTGGTGATGGCCCGGT
GCTGCTGCCGGATAATCATTATCTGAGCACCCAGAGCGTTCTGAGCAAAGATCCGAATGAAAAACGTGATCATATGGTGCTGC
TGGAATTTGTTACCGCCGCGGGCATTACCCACGGTATGGATGAACTGTATAAAGGCAGCCACCATCATCATCACCATTAA

Protein sequence of wild-type sfGFP:

MVSKGEELFTGVVPILVELDGDVNGHKFSVRGEGEGDATNGKLTLKFICTTGKLPVPWPTLVTTLTYGVQCFSRYPDHMKRHD
FFKSAMPEGYVQERTISFKDDGTYKTRAEVKFEGDTLVNRIELKGIDFKEDGNILGHKLEYNFNSHNVYITADKQKNGIKANF
KIRHNVEDGSVQLADHYQQNTPIGDGPVLLPDNHYLSTQSVLSKDPNEKRDHMVLLEFVTAAGITHGMDELYKGSHHHHHH*

DNA sequence of *Sc*SLAC:

ATGGCTCCTGCTGCAAAAGGTATTACTGCTCGTACTGCACCTGCTGGTGGCGAAGTGCGTCATCTCAAGATGTACGCTGAAAA
GCTGGCAGACGGTCAGATGGGCTACGGCTTCGAAAAGGGCAAGGCATCGGTCCCTGGCCCTCTGATCGAAGTCAACGAAGGCG
ACACTCTGCATATCGAATTCACCAACACTATGGACGTGCGTGCTAGTCTGCATGTGCATGGCCTGGACTACGAAATCTCCAGT
GACGGTACCGCAATGAACAAATCAGATGTTGAACCTGGTGGTACTCGTACTTATACATGGCGTACTCATAAACCTGGTCGTCG
TGACGACGGCACCTGGCGTCCTGGCAGTGCAGGCTACTGGCATTACCATGACCATGTCGTCGGCACCGAACATGGCACCGGAG
GCATCCGTAACGGCCTGTACGGCCCTGTGATCGTGCGTCGTAAGGGTGACGTGCTGCCTGACGCTACTCATACTATCGTCTTC
AACGACATGACCATCAACAACCGTAAACCTCATACCGGCCCTGACTTCGAAGCTACCGTGGGCGACCGTGTGGAAATCGTCAT
GATCACTCATGGTGAATACTACCATACCTTCCACATGCATGGTCATCGTTGGGCAGACAACCGTACCGGCATCCTCACCGGCC
CTGACGACCCTTCCCGTGTCATCGACAACAAGATCACCGGCCCTGCTGACTCCTTCGGCTTCCAGATCATCGCAGGTGAAGGT
GTGGGCGCTGGTGCATGGATGTACCATTGTCATGTCCAGAGTCATTCCGACATGGGCATGGTGGGTCTGTTCCTGGTGAAGAA
GCCTGACGGCACTATCCCTGGTTACGAACCTCATGAACATGGCGGTGCAACCGCAAAGAGTGGCGAAAGTGGTGAACCTACTG
GCGGTGCAGCTGCACATGAACATGAACATCTCGAGCACCACCACCACCACCACTGA

Protein Sequence of *Sc*SLAC:

MAPAAKGITARTAPAGGEVRHLKMYAEKLADGQMGYGFEKGKASVPGPLIEVNEGDTLHIEFTNTMDVRASLHVHGLDYEISS
DGTAMNKSDVEPGGTRTYTWRTHKPGRRDDGTWRPGSAGYWHYHDHVVGTEHGTGGIRNGLYGPVIVRRKGDVLPDATHTIVF
NDMTINNRKPHTGPDFEATVGDRVEIVMITHGEYYHTFHMHGHRWADNRTGILTGPDDPSRVIDNKITGPADSFGFQIIAGEG
VGAGAWMYHCHVQSHSDMGMVGLFLVKKPDGTIPGYEPHEHGGATAKSGESGEPTGGAAAHEHEHLEHHHHHH*

DNA sequence of *Sc*SLAC-M298$_{TAG}$:

ATGGCTCCTGCTGCAAAAGGTATTACTGCTCGTACTGCACCTGCTGGTGGCGAAGTGCGTCATCTCAAGATGTACGCTGAAAA
GCTGGCAGACGGTCAGATGGGCTACGGCTTCGAAAAGGGCAAGGCATCGGTCCCTGGCCCTCTGATCGAAGTCAACGAAGGCG
ACACTCTGCATATCGAATTCACCAACACTATGGACGTGCGTGCTAGTCTGCATGTGCATGGCCTGGACTACGAAATCTCCAGT
GACGGTACCGCAATGAACAAATCAGATGTTGAACCTGGTGGTACTCGTACTTATACATGGCGTACTCATAAACCTGGTCGTCG
TGACGACGGCACCTGGCGTCCTGGCAGTGCAGGCTACTGGCATTACCATGACCATGTCGTCGGCACCGAACATGGCACCGGAG
GCATCCGTAACGGCCTGTACGGCCCTGTGATCGTGCGTCGTAAGGGTGACGTGCTGCCTGACGCTACTCATACTATCGTCTTC
AACGACATGACCATCAACAACCGTAAACCTCATACCGGCCCTGACTTCGAAGCTACCGTGGGCGACCGTGTGGAAATCGTCAT
GATCACTCATGGTGAATACTACCATACCTTCCACATGCATGGTCATCGTTGGGCAGACAACCGTACCGGCATCCTCACCGGCC
CTGACGACCCTTCCCGTGTCATCGACAACAAGATCACCGGCCCTGCTGACTCCTTCGGCTTCCAGATCATCGCAGGTGAAGGT
GTGGGCGCTGGTGCATGGATGTACCATTGTCATGTCCAGAGTCATTCCGACATGGGCTAGGTGGGTCTGTTCCTGGTGAAGAA
GCCTGACGGCACTATCCCTGGTTACGAACCTCATGAACATGGCGGTGCAACCGCAAAGAGTGGCGAAAGTGGTGAACCTACTG
GCGGTGCAGCTGCACATGAACATGAACATCTCGAGCACCACCACCACCACCACTGA

Protein Sequence of *Sc*SLAC-M298$_{TAG}$:

MAPAAKGITARTAPAGGEVRHLKMYAEKLADGQMGYGFEKGKASVPGPLIEVNEGDTLHIEFTNTMDVRASLHVHGLDYEISS
DGTAMNKSDVEPGGTRTYTWRTHKPGRRDDGTWRPGSAGYWHYHDHVVGTEHGTGGIRNGLYGPVIVRRKGDVLPDATHTIVF
NDMTINNRKPHTGPDFEATVGDRVEIVMITHGEYYHTFHMHGHRWADNRTGILTGPDDPSRVIDNKITGPADSFGFQIIAGEG
VGAGAWMYHCHVQSHSDMG(ncAA)VGLFLVKKPDGTIPGYEPHEHGGATAKSGESGEPTGGAAAHEHEHLEHHHHHH*

DNA sequence of *Sc*SLAC-M298F:

ATGGCTCCTGCTGCAAAAGGTATTACTGCTCGTACTGCACCTGCTGGTGGCGAAGTGCGTCATCTCAAGATGTACGCTGAAAA
GCTGGCAGACGGTCAGATGGGCTACGGCTTCGAAAAGGGCAAGGCATCGGTCCCTGGCCCTCTGATCGAAGTCAACGAAGGCG
ACACTCTGCATATCGAATTCACCAACACTATGGACGTGCGTGCTAGTCTGCATGTGCATGGCCTGGACTACGAAATCTCCAGT
GACGGTACCGCAATGAACAAATCAGATGTTGAACCTGGTGGTACTCGTACTTATACATGGCGTACTCATAAACCTGGTCGTCG
TGACGACGGCACCTGGCGTCCTGGCAGTGCAGGCTACTGGCATTACCATGACCATGTCGTCGGCACCGAACATGGCACCGGAG
GCATCCGTAACGGCCTGTACGGCCCTGTGATCGTGCGTCGTAAGGGTGACGTGCTGCCTGACGCTACTCATACTATCGTCTTC
AACGACATGACCATCAACAACCGTAAACCTCATACCGGCCCTGACTTCGAAGCTACCGTGGGCGACCGTGTGGAAATCGTCAT
GATCACTCATGGTGAATACTACCATACCTTCCACATGCATGGTCATCGTTGGGCAGACAACCGTACCGGCATCCTCACCGGCC
CTGACGACCCTTCCCGTGTCATCGACAACAAGATCACCGGCCCTGCTGACTCCTTCGGCTTCCAGATCATCGCAGGTGAAGGT
GTGGGCGCTGGTGCATGGATGTACCATTGTCATGTCCAGAGTCATTCCGACATGGGCTTTGTGGGTCTGTTCCTGGTGAAGAA
GCCTGACGGCACTATCCCTGGTTACGAACCTCATGAACATGGCGGTGCAACCGCAAAGAGTGGCGAAAGTGGTGAACCTACTG
GCGGTGCAGCTGCACATGAACATGAACATCTCGAGCACCACCACCACCACCACTGA

Protein Sequence of *Sc*SLAC-M298F:

MAPAAKGITARTAPAGGEVRHLKMYAEKLADGQMGYGFEKGKASVPGPLIEVNEGDTLHIEFTNTMDVRASLHVHGLDYEISS
DGTAMNKSDVEPGGTRTYTWRTHKPGRRDDGTWRPGSAGYWHYHDHVVGTEHGTGGIRNGLYGPVIVRRKGDVLPDATHTIVF
NDMTINNRKPHTGPDFEATVGDRVEIVMITHGEYYHTFHMHGHRWADNRTGILTGPDDPSRVIDNKITGPADSFGFQIIAGEG
VGAGAWMYHCHVQSHSDMGFVGLFLVKKPDGTIPGYEPHEHGGATAKSGESGEPTGGAAAHEHEHLEHHHHHH*

DNA sequence of *Sc*SLAC-M298I:

ATGGCTCCTGCTGCAAAAGGTATTACTGCTCGTACTGCACCTGCTGGTGGCGAAGTGCGTCATCTCAAGATGTACGCTGAAAA
GCTGGCAGACGGTCAGATGGGCTACGGCTTCGAAAAGGGCAAGGCATCGGTCCCTGGCCCTCTGATCGAAGTCAACGAAGGCG
ACACTCTGCATATCGAATTCACCAACACTATGGACGTGCGTGCTAGTCTGCATGTGCATGGCCTGGACTACGAAATCTCCAGT
GACGGTACCGCAATGAACAAATCAGATGTTGAACCTGGTGGTACTCGTACTTATACATGGCGTACTCATAAACCTGGTCGTCG
TGACGACGGCACCTGGCGTCCTGGCAGTGCAGGCTACTGGCATTACCATGACCATGTCGTCGGCACCGAACATGGCACCGGAG
GCATCCGTAACGGCCTGTACGGCCCTGTGATCGTGCGTCGTAAGGGTGACGTGCTGCCTGACGCTACTCATACTATCGTCTTC
AACGACATGACCATCAACAACCGTAAACCTCATACCGGCCCTGACTTCGAAGCTACCGTGGGCGACCGTGTGGAAATCGTCAT
GATCACTCATGGTGAATACTACCATACCTTCCACATGCATGGTCATCGTTGGGCAGACAACCGTACCGGCATCCTCACCGGCC
CTGACGACCCTTCCCGTGTCATCGACAACAAGATCACCGGCCCTGCTGACTCCTTCGGCTTCCAGATCATCGCAGGTGAAGGT
GTGGGCGCTGGTGCATGGATGTACCATTGTCATGTCCAGAGTCATTCCGACATGGGCATTGTGGGTCTGTTCCTGGTGAAGAA
GCCTGACGGCACTATCCCTGGTTACGAACCTCATGAACATGGCGGTGCAACCGCAAAGAGTGGCGAAAGTGGTGAACCTACTG
GCGGTGCAGCTGCACATGAACATGAACATCTCGAGCACCACCACCACCACCACTGA

Protein Sequence of *Sc*SLAC-M298I:

MAPAAKGITARTAPAGGEVRHLKMYAEKLADGQMGYGFEKGKASVPGPLIEVNEGDTLHIEFTNTMDVRASLHVHGLDYEISS
DGTAMNKSDVEPGGTRTYTWRTHKPGRRDDGTWRPGSAGYWHYHDHVVGTEHGTGGIRNGLYGPVIVRRKGDVLPDATHTIVF
NDMTINNRKPHTGPDFEATVGDRVEIVMITHGEYYHTFHMHGHRWADNRTGILTGPDDPSRVIDNKITGPADSFGFQIIAGEG
VGAGAWMYHCHVQSHSDMGIVGLFLVKKPDGTIPGYEPHEHGGATAKSGESGEPTGGAAAHEHEHLEHHHHHH*

DNA sequence of *Sc*SLAC-M298L:

ACCGCAATGAACAAATCAGATGTTGAACCTGGTGGTACTCGTACTTATACATGGCGTACTCATAAACCTGGTCGTCGTGACGA
CGGCACCTGGCGTCCTGGCAGTGCAGGCTACTGGCATTACCATGACCATGTCGTCGGCACCGAACATGGCACCGGAGGCATCC
GTAACGGCCTGTACGGCCCTGTGATCGTGCGTCGTAAGGGTGACGTGCTGCCTGACGCTACTCATACTATCGTCTTCAACGAC
ATGACCATCAACAACCGTAAACCTCATACCGGCCCTGACTTCGAAGCTACCGTGGGCGACCGTGTGGAAATCGTCATGATCAC
TCATGGTGAATACTACCATACCTTCCACATGCATGGTCATCGTTGGGCAGACAACCGTACCGGCATCCTCACCGGCCCTGACG
ACCCTTCCCGTGTCATCGACAACAAGATCACCGGCCCTGCTGACTCCTTCGGCTTCCAGATCATCGCAGGTGAAGGTGTGGGC
GCTGGTGCATGGATGTACCATTGTCATGTCCAGAGTCATTCCGACATGGGCCTGGTGGGTCTGTTCCTGGTGAAGAAGCCTGA
CGGCACTATCCCTGGTTACGAACCTCATGAACATGGCGGTGCAACCGCAAAGAGTGGCGAAAGTGGTGAACCTACTGGCGGTG
CAGCTGCACATGAACATGAACATCTCGAGCACCACCACCACCACCACTGA

Protein Sequence of *Sc*SLAC-M298L:

MAPAAKGITARTAPAGGEVRHLKMYAEKLADGQMGYGFEKGKASVPGPLIEVNEGDTLHIEFTNTMDVRASLHVHGLDYEISS
DGTAMNKSDVEPGGTRTYTWRTHKPGRRDDGTWRPGSAGYWHYHDHVVGTEHGTGGIRNGLYGPVIVRRKGDVLPDATHTIVF
NDMTINNRKPHTGPDFEATVGDRVEIVMITHGEYYHTFHMHGHRWADNRTGILTGPDDPSRVIDNKITGPADSFGFQIIAGEG
VGAGAWMYHCHVQSHSDMGLVGLFLVKKPDGTIPGYEPHEHGGATAKSGESGEPTGGAAAHEHEHLEHHHHHH*

DNA sequence of *Sc*SLAC-M298Q:

ATGGCTCCTGCTGCAAAAGGTATTACTGCTCGTACTGCACCTGCTGGTGGCGAAGTGCGTCATCTCAAGATGTACGCTGAAAA
GCTGGCAGACGGTCAGATGGGCTACGGCTTCGAAAAGGGCAAGGCATCGGTCCCTGGCCCTCTGATCGAAGTCAACGAAGGCG
ACACTCTGCATATCGAATTCACCAACACTATGGACGTGCGTGCTAGTCTGCATGTGCATGGCCTGGACTACGAAATCTCCAGT
GACGGTACCGCAATGAACAAATCAGATGTTGAACCTGGTGGTACTCGTACTTATACATGGCGTACTCATAAACCTGGTCGTCG
TGACGACGGCACCTGGCGTCCTGGCAGTGCAGGCTACTGGCATTACCATGACCATGTCGTCGGCACCGAACATGGCACCGGAG
GCATCCGTAACGGCCTGTACGGCCCTGTGATCGTGCGTCGTAAGGGTGACGTGCTGCCTGACGCTACTCATACTATCGTCTTC
AACGACATGACCATCAACAACCGTAAACCTCATACCGGCCCTGACTTCGAAGCTACCGTGGGCGACCGTGTGGAAATCGTCAT
GATCACTCATGGTGAATACTACCATACCTTCCACATGCATGGTCATCGTTGGGCAGACAACCGTACCGGCATCCTCACCGGCC
CTGACGACCCTTCCCGTGTCATCGACAACAAGATCACCGGCCCTGCTGACTCCTTCGGCTTCCAGATCATCGCAGGTGAAGGT
GTGGGCGCTGGTGCATGGATGTACCATTGTCATGTCCAGAGTCATTCCGACATGGGCCAGGTGGGTCTGTTCCTGGTGAAGAA
GCCTGACGGCACTATCCCTGGTTACGAACCTCATGAACATGGCGGTGCAACCGCAAAGAGTGGCGAAAGTGGTGAACCTACTG
GCGGTGCAGCTGCACATGAACATGAACATCTCGAGCACCACCACCACCACCACTGA

Protein Sequence of *Sc*SLAC-M298Q:

MAPAAKGITARTAPAGGEVRHLKMYAEKLADGQMGYGFEKGKASVPGPLIEVNEGDTLHIEFTNTMDVRASLHVHGLDYEISS
DGTAMNKSDVEPGGTRTYTWRTHKPGRRDDGTWRPGSAGYWHYHDHVVGTEHGTGGIRNGLYGPVIVRRKGDVLPDATHTIVF
NDMTINNRKPHTGPDFEATVGDRVEIVMITHGEYYHTFHMHGHRWADNRTGILTGPDDPSRVIDNKITGPADSFGFQIIAGEG
VGAGAWMYHCHVQSHSDMGQVGLFLVKKPDGTIPGYEPHEHGGATAKSGESGEPTGGAAAHEHEHLEHHHHHH*

DNA sequence of *Sc*SLAC-S292P-M296W-M298$_{TAG}$:

ATGGCTCCTGCTGCAAAAGGTATTACTGCTCGTACTGCACCTGCTGGTGGCGAAGTGCGTCATCTCAAGATGTACGCTGAAAA
GCTGGCAGACGGTCAGATGGGCTACGGCTTCGAAAAGGGCAAGGCATCGGTCCCTGGCCCTCTGATCGAAGTCAACGAAGGCG
ACACTCTGCATATCGAATTCACCAACACTATGGACGTGCGTGCTAGTCTGCATGTGCATGGCCTGGACTACGAAATCTCCAGT
GACGGTACCGCAATGAACAAATCAGATGTTGAACCTGGTGGTACTCGTACTTATACATGGCGTACTCATAAACCTGGTCGTCG
TGACGACGGCACCTGGCGTCCTGGCAGTGCAGGCTACTGGCATTACCATGACCATGTCGTCGGCACCGAACATGGCACCGGAG
GCATCCGTAACGGCCTGTACGGCCCTGTGATCGTGCGTCGTAAGGGTGACGTGCTGCCTGACGCTACTCATACTATCGTCTTC
AACGACATGACCATCAACAACCGTAAACCTCATACCGGCCCTGACTTCGAAGCTACCGTGGGCGACCGTGTGGAAATCGTCAT
GATCACTCATGGTGAATACTACCATACCTTCCACATGCATGGTCATCGTTGGGCAGACAACCGTACCGGCATCCTCACCGGCC
CTGACGACCCTTCCCGTGTCATCGACAACAAGATCACCGGCCCTGCTGACTCCTTCGGCTTCCAGATCATCGCAGGTGAAGGT
GTGGGCGCTGGTGCATGGATGTACCATTGTCATGTCCAGCCGCATTCCGACTGGGGCTAGGTGGGTCTGTTCCTGGTGAAGAA

GCCTGACGGCACTATCCCTGGTTACGAACCTCATGAACATGGCGGTGCAACCGCAAAGAGTGGCGAAAGTGGTGAACCTACTG
GCGGTGCAGCTGCACATGAACATGAACATCTCGAGCACCACCACCACCACCACTGA

Protein Sequence of *Sc*SLAC-S292P-M296W-M298$_{TAG}$:

MAPAAKGITARTAPAGGEVRHLKMYAEKLADGQMGYGFEKGKASVPGPLIEVNEGDTLHIEFTNTMDVRASLHVHGLDYEISS
DGTAMNKSDVEPGGTRTYTWRTHKPGRRDDGTWRPGSAGYWHYHDHVVGTEHGTGGIRNGLYGPVIVRRKGDVLPDATHTIVF
NDMTINNRKPHTGPDFEATVGDRVEIVMITHGEYYHTFHMHGHRWADNRTGILTGPDDPSRVIDNKITGPADSFGFQIIAGEG
VGAGAWMYHCHVQPHSDWG(ncAA)VGLFLVKKPDGTIPGYEPHEHGGATAKSGESGEPTGGAAAHEHEHLEHHHHHH*

V. Plasmid Construction

pDPS2-*Mm*tRNA$^{Pyl}_{CUA}$ plasmid (GenBank: PX848774):

The pDPS2-*Mm*tRNA$^{Pyl}_{CUA}$ plasmid contains a p15a origin of replication, a tetracycline resistance gene (TetR), an sfGFP gene containing an amber mutation at position 150 under control of an L-arabinose inducible araBAD promoter, a chloramphenicol resistance gene with an amber mutation at position 111 and an *Mm*tRNA$^{Pyl}_{CUA}$ tRNA flanked by a proK promoter and terminator.

TetR; ***Mm*tRNA$^{Pyl}_{CUA}$**; **cat-promoter/cat111TAG**; **p15A ori**; **araC**; **araBAD**; **sfGFP150$_{TAG}$-6xHis**.

GAGAGAAGATTTTCAGCCTGATACAGATTAAATCAGAACGCAGAAGCGGTCTGATAAAACAGAATTTGCCTGGCGGCAGTAGC
GCGGTGGTCCCACCTGACCCCATGCCGAACTCAGAAGTGAAACGCCGTAGCGCCGATGGTAGTGTAGCACCTGAAGTCAGCCC
CATACGATATAAGTTGTAATTCTCATGT**TTGACAGCTTATCATCGATAAGCTTTAATGCGGTAGTTTATCACAGTTAAATTGC**
TAACGCAGTCAGGCACCGTGTATGAAATCTAACAATGCGCTCATCGTCATCCTCGGCACCGTCACCCTGGATGCTGTAGGCAT
AGGCTTGGTTATGCCGGTACTGCCGGGCCTCTTGCGGGATATCGTCCATTCCGACAGCATCGCCAGTCACTATGGCGTGCTGC
TAGCGCTATATGCGTTGATGCAATTTCTATGCGCACCCGTTCTCGGAGCACTGTCCGACCGCTTTGGCCGCCGCCCAGTCCTG
CTCGCTTCGCTACTTGGAGCCACTATCGACTACGCGATCATGGCGACCACACCCGTCCTGTGGATCCTCTACGCCGGACGCAT
CGTGGCCGGCATCACCGGCGCCACAGGTGCGGTTGCTGGCGCCTATATCGCCGACATCACCGATGGGGAAGATCGGGCTCGCC
ACTTCGGGCTCATGAGCGCTTGTTTCGGCGTGGGTATGGTGGCAGGCCCCGTGGCCGGGGGACTGTTGGGCGCCATCTCCTTG
CATGCACCATTCCTTGCGGCGGCGGTGCTCAACGGCCTCAACCTACTACTGGGCTGCTTCCTAATGCAGGAGTCGCATAAGGG
AGAGCGTCGACCGATGCCCTTGAGAGCCTTCAACCCAGTCAGCTCCTTCCGGTGGGCGCGGGGCATGACTATCGTCGCCGCAC
TTATGACTGTCTTCTTTATCATGCAACTCGTAGGACAGGTGCCGGCAGCGCTCTGGGTCATTTTCGGCGAGGACCGCTTTCGC
TGGAGCGCGACGATGATCGGCCTGTCGCTTGCGGTATTCGGAATCTTGCACGCCCTCGCTCAAGCCTTCGTCACTGGTCCCGC
CACCAAACGTTTCGGCGAGAAGCAGGCCATTATCGCCGGCATGGCGGCCGACGCGCTGGGCTACGTCTTGCTGGCGTTCGCGA
CGCGAGGCTGGATGGCCTTCCCCATTATGATTCTTCTCGCTTCCGGCGGCATCGGGATGCCCGCGTTGCAGGCCATGCTGTCC
AGGCAGGTAGATGACGACCATCAGGGACAGCTTCAAGGATCGCTCGCGGCTCTTACCAGCCTAACTTCGATCATTGGACCGCT
GATCGTCACGGCGATTTATGCCGCCTCGGCGAGCACATGGAACGGGTTGGCATGGATTGTAGGCGCCGCCCTATACCTTGTCT
GCCTCCCCGCGTTGCGTCGCGGTGCATGGAGCCGGGCCACCTCGACCTAATAAAACGAAAGGCTCAGTCGAAAGACTGGGCCT
TGTTTGTGAGCTCCCGGTCATCAATCATCCCCATAATCCTTGTTAGATTATCAATTTTAAAAAACTAACAGTTGTCAGCCTGT
CCCGCTTTAATATCATACGCCGTTATACGTTGTTTACGCTTTGAGGAATCCCATATGATACGCGGCCGCTTTCAAACGCTAAA
TTGCCTGATGCGCTACGCTTATCAGGCCTACATGATCTCTGCAATATATTGAGTTTGCGTGCTTTTGTAGGCCGGATAAGGCG
TTCACGCCGCATCCGGCAAGAAACAGCAAACAATCCAAAACGCCGCGTTCAGCGGCGTTTTTTCTGCTTTTCTTCGCGAATTA
ATTCCGCTTCGCAACATGTGAGCACCGGTTTATTGACTACCGGAAGCAGTGTGACCGTGTGCTTCTCAAATGCCTGAGGCCAG
TTTGCTCAGGCTCTCCCCGTGGAGGTAATAATTGACGATATGATCAGTGCACGGCTAACTAAGCGGCCTGCTGACTTTCTCGC
CGATCAAAAGGCATTTTGCTATTAAGGGATTGACGAGGGCGTATCTGCGCAGTAAGATGCGCCCCGCATT**GGAAACCTGATCA**
TGTAGATCGAATGGACTCTAAATCCGTTCAGCCGGGTTAGATTCCCGGGGTTTCCGCCAAATTCGAAAAGCCTGCTCAACGAG
CAGGCTTTTTTGCATGCTCGAGCAGCTCAGGGTCGAATTTGCTTTCGAATTTCTGCCATTCATCCGCTTATTATCACTTATTC
AGGCGTAGCAACCAGGCGTTTAAGGGCACCAATAACTGCCTTAAAAAAA**TTACGCCCCGCCCTGCCACTCATCGCAGTACTGT**
TGTAATTCATTAAGCATTCTGCCGACATGGAAGCCATCACAAACGGCATGATGAACCTGAATCGCCAGCGGCATCAGCACCTT
GTCGCCTTGCGTATAATATTTGCCCATGGTGAAAACGGGGGCGAAGAAGTTGTCCATATTGGCCACGTTTAAATCAAAACTGG
TGAAACTCACCCAGGGATTGGCTGAGACGAAAAACATATTCTCAATAAACCCTTTAGGGAAATAGGCCAGGTTTTCACCGTAA
CACGCCACATCTTGCGAATATATGTGTAGAAACTGCCGGAAATCCTAGTGGTATTCACTCCAGAGCGATGAAAACGTTTCAGT
TTGCTCATGGAAAACGGTGTAACAAGGGTGAACACTATCCCATATCACCAGCTCACCGTCTTTCATTGCCATACGGAATTCCG
GATGAGCATTCATCAGGCGGGCAAGAATGTGAATAAAGGCCGGATAAAACTTGTGCTTATTTTTCTTTACGGTCTTTAAAAAG
GCCGTAATATCCAGCTGAACGGTCTGGTTATAGGTACATTGAGCAACTGACTGAAATGCCTCAAAATGTTCTTTACGATGCCA
TTGGGATATATCAACGGTGGTATATCCAGTGATTTTTTTCTCCATTTTAGCTTCCTTAGCTCCTGAAAATCTCGATAACTCAA
AAAATACGCCCGGTAGTGATCTTATTTCATTATGGTGAAAGTTGGAACCTCTTACGTGCCGATCAACGTCTCATTTTCGCCAA
AAGTTGGCCCAGGGCTTCCCGGTATCAACAGGGACACCAGGATTTATTTATTCTGCGAAGTGATCTTCCGTCACAGGTATTTA
TTCGGCGCAAAGTGCGTCGGGTGATGCTGCCAACTTACTGATTTAGTGTATGATGGTGTTTTTGAGGTGCTCCAGTGGCTTCT
GTTTCTATCAGCTGTCCCTCCTGTTCAGCTACTGACGGGGTGGTGCGTAACGGCAAAAGCACCGCCGGACATCAGCGCTAGCG
GAGTGTATACTGGCTTACTATGTTGGCACTGATGAGGGTGTCAGTGAAGTGCTTCATGTGGCAGGAGAAAAAAGGCTGCACCG
GTGCGTCAGCAGAATATGTGATACAGGATATATTCCGCTTCCTCGCTCACTGACTCGCTACGCTCGGTCGTTCGACTGCGGCG
AGCGGAAATGGCTTACGAACGGGGCGGAGATTTCCTGGAAGATGCCAGGAAGATACTTAACAGGGAAGTGAGAGGGCCGCGGC
AAAGCCGTT**TTTCCATAGGCTCCGCCCCCCTGACAAGCATCACGAAATCTGACGCTCAAATCAGTGGTGGCGAAACCCGACAG**
GACTATAAAGATACCAGGCGTTTCCCCCTGGCGGCTCCCTCGTGCGCTCTCCTGTTCCTGCCTTTCGGTTTACCGGTGTCATT
CCGCTGTTATGGCCGCGTTTGTCTCATTCCACGCCTGACACTCAGTTCCGGGTAGGCAGTTCGCTCCAAGCTGGACTGTATGC
ACGAACCCCCCGTTCAGTCCGACCGCTGCGCCTTATCCGGTAACTATCGTCTTGAGTCCAACCCGGAAAGACATGCAAAAGCA
CCACTGGCAGCAGCCACTGGTAATTGATTTAGAGGAGTTAGTCTTGAAGTCATGCGCCGGTTAAGGCTAAACTGAAAGGACAA
GTTTTGGTGACTGCGCTCCTCCAAGCCAGTTACCTCGGTTCAAAGAGTTGGTAGCTCAGAGAACCTTCGAAAAACCGCCCTGC
AAGGCGGTTTTTTCGTTTTCAGAGCAAGAGATTACGCGCAGACCAAAACGATCTCAAGAAGATCATCTTATTAATCAGATAAA
ATATTTCTAGATTTCAGTGCAATTTATCTCTTCAAATGTAGCACCTGAAGTCAGCCCCATACGATATAAGTTGTAATTCTCAT
GTTTGACAGCTTATCATCGATAAGCTTGGTACCCAA**TTATGACAACTTGACGGCTACATCATTCACTTTTTCTTCACAACCGG**
CACGGAACTCGCTCGGGCTGGCCCCGGTGCATTTTTTAAATACCCGCGAGAAATAGAGTTGATCGTCAAAACCAACATTGCGA
CCGACGGTGGCGATAGGCATCCGGGTGGTGCTCAAAAGCAGCTTCGCCTGGCTGATACGTTGGTCCTCGCGCCAGCTTAAGAC
GCTAATCCCTAACTGCTGGCGGAAAAGATGTGACAGACGCGACGGCGACAAGCAAACATGCTGTGCGACGCTGGCGATATCAA

AATTGCTGTCTGCCAGGTGATCGCTGATGTACTGACAAGCCTCGCGTACCCGATTATCCATCGGTGGATGGAGCGACTCGTTA
ATCGCTTCCATGCGCCGCAGTAACAATTGCTCAAGCAGATTTATCGCCAGCAGCTCCGAATAGCGCCCTTCCCCTTGCCCGGC
GTTAATGATTTGCCCAAACAGGTCGCTGAAATGCGGCTGGTGCGCTTCATCCGGGCGAAAGAACCCCGTATTGGCAAATATTG
ACGGCCAGTTAAGCCATTCATGCCAGTAGGCGCGCGGACGAAAGTAAACCCACTGGTGATACCATTCGCGAGCCTCCGGATGA
CGACCGTAGTGATGAATCTCTCCTGGCGGGAACAGCAAAATATCACCCGGTCGGCAAACAAATTCTCGTCCCTGATTTTTCAC
CACCCCCTGACCGCGAATGGTGAGATTGAGAATATAACCTTTCATTCCCAGCGGTCGGTCGATAAAAAAATCGAGATAACCGT
TGGCCTCAATCGGCGTTAAACCCGCCACCAGATGGGCATTAAACGAGTATCCCGGCAGCAGGGGATCATTTTGCGCTTCAGCC
ATACTTTTCATACTCCCGCCATTCAGAG**AAGAAACCAATTGTCCATATTGCATCAGACATTGCCGTCACTGCGTCTTTTACTG**
GCTCTTCTCGCTAACCAAACCGGTAACCCCGCTTATTAAAAGCATTCTGTAACAAAGCGGGACCAAAGCCATGACAAAAACGC
GTAACAAAAGTGTCTATAATCACGGCAGAAAAGTCCACATTGATTATTTGCACGGCGTCACACTTTGCTATGCCATAGCATTT
TTATCCATAAGATTAGCGGATCCTACCTGACGCTTTTTATCGCAACTCTCTACTGTTTCTCCATACCCGTTTTTTGGGCTAAC
AGGAGGAATTAACC**ATGGTTAGCAAAGGTGAAGAACTGTTTACCGGCGTTGTGCCGATTCTGGTGGAACTGGATGGTGATGTG**
AATGGCCATAAATTTAGCGTTCGTGGCGAAGGCGAAGGTGATGCGACCAACGGTAAACTGACCCTGAAATTTATTTGCACCAC
CGGTAAACTGCCGGTTCCGTGGCCGACCCTGGTGACCACCCTGACCTATGGCGTTCAGTGCTTTAGCCGCTATCCGGATCATA
TGAAACGCCATGATTTCTTTAAAAGCGCGATGCCGGAAGGCTATGTGCAGGAACGTACCATTAGCTTCAAAGATGATGGCACC
TATAAAACCCGTGCGGAAGTTAAATTTGAAGGCGATACCCTGGTGAACCGCATTGAACTGAAAGGTATTGATTTTAAAGAAGA
TGGCAACATTCTGGGTCATAAACTGGAATATAATTTCAACAGCCATTAGGTGTATATTACCGCCGATAAACAGAAAAATGGCA
TCAAAGCGAACTTTAAAATCCGTCACAACGTGGAAGATGGTAGCGTGCAGCTGGCGGATCATTATCAGCAGAATACCCCGATT
GGTGATGGCCCGGTGCTGCTGCCGGATAATCATTATCTGAGCACCCAGAGCGTTCTGAGCAAAGATCCGAATGAAAAACGTGA
TCATATGGTGCTGCTGGAATTTGTTACCGCCGCGGGCATTACCCACGGTATGGATGAACTGTATAAAGGCAGCCACCATCATC
ATCACCATTAAAGCTCGAGCGAAGCTTGGGCCCGAACAAAAACTCATCTCAGAAGAGGATCTGAATAGCGCCGTCGACCATCA
TCATCATCATCATTGAGTTTAAACGGTCTCCAGCTTGGCTGTTTTGGCGGATGAGAGAAGATTTTCAGCCTGATACAGATTAA
ATCAGAACGCAGAAGCGGTCTGATAAAACAGAATTTGCCTGGCGGCAGTAGCGCGGTGGTCCCACCTGACCCCATGCCGAACT
CAGAAGTGAAACGCCGTAGCGCCGATGGTAGTGTGGGGTCTCCCCATGCGAGAGTAGGGAACTGCCAGGCATCAAATAAAACG
AAAGGCTCAGTCGAAAGACTGGGCCTTTCGTTTTATCTGTTGTTTGTCGGTGAACGCTCTCCTGAGTAGGACAAATCCTGGCT
GTTTTGGCGGAT

DNA sequence of pSL-*Mm*PylRS (GenBank: PX848775):

The pSL-*Mm*PylRS plasmid contains a pBR322 type origin of replication, a kanamycin resistance gene (KanR), and an *Mm*PylRS under control of a glnS promoter.

***Mm*PylRS; KanR; pBR322 ori**.

CGAACTTTTGCTGAGTTGAAGGATCCTCGGGTTGTCAGCCTGTCCCGCTTATAAGATCATACGCCGTTATACGTTGTTTACGC
TTTGAGGAATCCCAT**ATGGATAAAAAACCGCTGAACACCCTGATTAGCGCGACCGGCCTGTGGATGAGCCGCACCGGCACCAT**
TCATAAAATTAAACATCATGAAGTGAGCCGCAGCAAAATTTATATTGAAATGGCGTGCGGCGATCATCTGGTGGTGAACAACA
GCCGCAGCAGCCGCACCGCGCGCGCGCTGCGCCATCATAAATATCGCAAAACCTGCAAACGCTGCCGCGTGAGCGATGAAGAT
CTGAACAAATTTCTGACCAAAGCGAACGAAGATCAGACCAGCGTGAAAGTGAAAGTGGTGAGCGCGCCGACCCGCACCAAAAA
AGCGATGCCGAAAAGCGTGGCGCGCGCGCCGAAACCGCTGGAAAACACCGAAGCGGCGCAGGCGCAGCCGAGCGGCAGCAAAT
TTAGCCCGGCGATTCCGGTGAGCACCCAGGAAAGCGTGAGCGTGCCGGCGAGCGTGAGCACCAGCATTAGCAGCATTAGCACC
GGCGCGACCGCGAGCGCGCTGGTGAAAGGCAACACCAACCCGATTACCAGCATGAGCGCGCCGGTGCAGGCGAGCGCGCCGGC
GCTGACCAAAAGCCAGACCGATCGCCTGGAAGTGCTGCTGAACCCGAAAGATGAAATTAGCCTGAACAGCGGCAAACCGTTTC
GCGAACTGGAAAGCGAACTGCTGAGCCGCCGCAAAAAAGATCTGCAGCAGATTTATGCGGAAGAACGCGAAAACTATCTGGGC
AAACTGGAACGCGAAATTACCCGCTTTTTTGTGGATCGCGGCTTTCTGGAAATTAAAAGCCCGATTCTGATTCCGCTGGAATA
TATTGAACGCATGGGCATTGATAACGATACCGAACTGAGCAAACAGATTTTTCGCGTGGATAAAAACTTTTGCCTGCGCCCGA
TGCTGGCGCCGAACCTGTATAACTATCTGCGCAAACTGGATCGCGCGCTGCCGGATCCGATTAAAATTTTTGAAATTGGCCCG
TGCTATCGCAAAGAAAGCGATGGCAAAGAACATCTGGAAGAATTTACCATGCTGAACTTTTGCCAGATGGGCAGCGGCTGCAC
CCGCGAAAACCTGGAAAGCATTATTACCGATTTTCTGAACCATCTGGGCATTGATTTTAAAATTGTGGGCGATAGCTGCATGG
TGTATGGCGATACCCTGGATGTGATGCATGGCGATCTGGAACTGAGCAGCGCGGTGGTGGGCCCGATTCCGCTGGATCGCGAA
TGGGGCATTGATAAACCGTGGATTGGCGCGGGCTTTGGCCTGGAACGCCTGCTGAAAGTGAAACATGATTTTAAAAACATTAA
ACGCGCGGCGCGCAGCGAAAGCTATTATAACGGCATTAGCACCAACCTGTAAAGGTGGCACTTTTCGGGGAAATGTGCGCGGA
ACCCCTATTTGTTTATTTTTCTAAATACATTCAAATATGTATCCGCTCATGAATTAATTC**TTAGAAAAACTCATCGAGCATCA**
AATGAAACTGCAATTTATTCATATCAGGATTATCAATACCATATTTTTGAAAAAGCCGTTTCTGTAATGAAGGAGAAAACTCA
CCGAGGCAGTTCCATAGGATGGCAAGATCCTGGTATCGGTCTGCGATTCCGACTCGTCCAACATCAATACAACCTATTAATTT
CCCCTCGTCAAAAATAAGGTTATCAAGTGAGAAATCACCATGAGTGACGACTGAATCCGGTGAGAATGGCAAAAGTTTATGCA
TTTCTTTCCAGACTTGTTCAACAGGCCAGCCATTACGCTCGTCATCAAAATCACTCGCATCAACCAAACCGTTATTCATTCGT
GATTGCGCCTGAGCGAGACGAAATACGCGATCGCTGTTAAAAGGACAATTACAAACAGGAATCGAATGCAACCGGCGCAGGAA
CACTGCCAGCGCATCAACAATATTTTCACCTGAATCAGGATATTCTTCTAATACCTGGAATGCTGTTTTCCCGGGGATCGCAG
TGGTGAGTAACCATGCATCATCAGGAGTACGGATAAAATGCTTGATGGTCGGAAGAGGCATAAATTCCGTCAGCCAGTTTAGT
CTGACCATCTCATCTGTAACATCATTGGCAACGCTACCTTTGCCATGTTTCAGAAACAACTCTGGCGCATCGGGCTTCCCATA
CAATCGATAGATTGTCGCACCTGATTGCCCGACATTATCGCGAGCCCATTTATACCCATATAAATCAGCATCCATGTTGGAAT
TTAATCGCGGCCTAGAGCAAGACGTTTCCCGTTGAATATGGCTCATAACACCCCTTGTATTACTGTTTATGTAAGCAGACAGT
TTTATTGTTCATGACCAAAATCCCTTAACGTGAGTTTTCGTTCCACTGAGCGTCAGACCCCGTAGAAAAGATCAAAGGATCTT
C**TTGAGATCCTTTTTTTCTGCGCGTAATCTGCTGCTTGCAAACAAAAAAACCACCGCTACCAGCGGTGGTTTGTTTGCCGGAT**
CAAGAGCTACCAACTCTTTTTCCGAAGGTAACTGGCTTCAGCAGAGCGCAGATACCAAATACTGTCCTTCTAGTGTAGCCGTA

GTTAGGCCACCACTTCAAGAACTCTGTAGCACCGCCTACATACCTCGCTCTGCTAATCCTGTTACCAGTGGCTGCTGCCAGTG
GCGATAAGTCGTGTCTTACCGGGTTGGACTCAAGACGATAGTTACCGGATAAGGCGCAGCGGTCGGGCTGAACGGGGGGTTCG
TGCACACAGCCCAGCTTGGAGCGAACGACCTACACCGAACTGAGATACCTACAGCGTGAGCTATGAGAAAGCGCCACGCTTCC
CGAAGGGAGAAAGGCGGACAGGTATCCGGTAAGCGGCAGGGTCGGAACAGGAGAGCGCACGAGGGAGCTTCCAGGGGGAAACG
CCTGGTATCTTTATAGTCCTGTCGGGTTTCGCCACCTCTGACTTGAGCGTCGATTTTTGTGATGCTCGTCAGGGGGGCGGAGC
CTATGGAAAAACGCCAGCAACGCGGCCTTTTTACGGTTCCTGGCCTTTTGCTGGCCTTTTGCTCACATGTTCTTTCCTGCGTT
ATCCCCTGATTCTGTGGATAACCGTATTACCGCCTTTGAGTGAGCTGATACCGCTCGCCGCAGCCGAACGACCGAGCGCAGCG
AGTCAGTGAGCGAGGAAGCGGAAGAG

DNA sequence of pBARN-*Mm*tRNA$^{Pyl}_{CUA}$ (GenBank: PX848776):

The pBARN-*Mm*tRNA$^{Pyl}_{CUA}$ plasmid contains a p15A type origin of replication, a chloramphenicol resistance gene (CamR), and an *Mm*tRNA$^{Pyl}_{CUA}$ under control of a proK promoter.

***Mm*tRNA$^{Pyl}_{CUA}$**; **CamR**; **p15A ori**; araBAD-barnase(3TAG, 45TAG, 66TAG).

GGCACCTCGCTAACGGACGCCTGAATAAGTGATAATAAGCGGATGAATGGCAGAAATTCGAAAGCAAATTCGACCCTGAGCTG
CTCGAGCATGCAAGGCATTTTGCTATTAAGGGATTGACGAGGGCGTATCTGCGCAGTAAGATGCGCCCCGCATT**GGAAACCTG**
ATCATGTAGATCGAATGGACTCTAAATCCGTTCAGCCGGGTTAGATTCCCGGGGTTTCCGCCAAATTCGAAAAGCCTGCTCAA
CGAGCAGGCTTTTTTGCATGCTCGAGCAGCTCAGGGTCGAATTTGCTTTCGAATTTCTGCCATTCATCCGCTTATTATCACTT
ATTCAGGCGTAGCAACCAGGCGTTTAAGGGCACCAATAACTGCCTTAAAAAAA**TTACGCCCCGCCCTGCCACTCATCGCAGTA**
CTGTTGTAATTCATTAAGCATTCTGCCGACATGGAAGCCATCACAAACGGCATGATGAACCTGAATCGCCAGCGGCATCAGCA
CCTTGTCGCCTTGCGTATAATATTTGCCCATGGTGAAAACGGGGGCGAAGAAGTTGTCCATATTGGCCACGTTTAAATCAAAA
CTGGTGAAACTCACCCAGGGATTGGCTGAGACGAAAAACATATTCTCAATAAACCCTTTAGGGAAATAGGCCAGGTTTTCACC
GTAACACGCCACATCTTGCGAATATATGTGTAGAAACTGCCGGAAATCGTCGTGGTATTCACTCCAGAGCGATGAAAACGTTT
CAGTTTGCTCATGGAAAACGGTGTAACAAGGGTGAACACTATCCCATATCACCAGCTCACCGTCTTTCATTGCCATACGGAAT
TCCGGATGAGCATTCATCAGGCGGGCAAGAATGTGAATAAAGGCCGGATAAAACTTGTGCTTATTTTTCTTTACGGTCTTTAA
AAAGGCCGTAATATCCAGCTGAACGGTCTGGTTATAGGTACATTGAGCAACTGACTGAAATGCCTCAAAATGTTCTTTACGAT
GCCATTGGGATATATCAACGGTGGTATATCCAGTGATTTTTTTCTCCATTTTAGCTTCCTTAGCTCCTGAAAATCTCGATAAC
TCAAAAAATACGCCCGGTAGTGATCTTATTTCATTATGGTGAAAGTTGGAACCTCTTACGTGCCGATCAACGTCTCATTTTCG
CCAAAAGTTGGCCCAGGGCTTCCCGGTATCAACAGGGACACCAGGATTTATTTATTCTGCGAAGTGATCTTCCGTCACAGGTA
TTTATTCGGCGCAAAGTGCGTCGGGTGATGCTGCCAACTTACTGATTTAGTGTATGATGGTGTTTTTGAGGTGCTCCAGTGGC
TTCTGTTTCTATCAGCTGTCCCTCCTGTTCAGCTACTGACGGGGTGGTGCGTAACGGCAAAAGCACCGCCGGACATCAGCGCT
AGCGGAGTGTATACTGGCTTACTATGTTGGCACTGATGAGGGTGTCAGTGAAGTGCTTCATGTGGCAGGAGAAAAAAGGCTGC
ACCGGTGCGTCAGCAGAATATGTGATACAGGATATATTCCGCTTCCTCGCTCACTGACTCGCTACGCTCGGTCGTTCGACTGC
GGCGAGCGGAAATGGCTTACGAACGGGGCGGAGATTTCCTGGAAGATGCCAGGAAGATACTTAACAGGGAAGTGAGAGGGCCG
CGGCAAAGCCGTT**TTTCCATAGGCTCCGCCCCCCTGACAAGCATCACGAAATCTGACGCTCAAATCAGTGGTGGCGAAACCCG**
ACAGGACTATAAAGATACCAGGCGTTTCCCCCTGGCGGCTCCCTCGTGCGCTCTCCTGTTCCTGCCTTTCGGTTTACCGGTGT
CATTCCGCTGTTATGGCCGCGTTTGTCTCATTCCACGCCTGACACTCAGTTCCGGGTAGGCAGTTCGCTCCAAGCTGGACTGT
ATGCACGAACCCCCCGTTCAGTCCGACCGCTGCGCCTTATCCGGTAACTATCGTCTTGAGTCCAACCCGGAAAGACATGCAAA
AGCACCACTGGCAGCAGCCACTGGTAATTGATTTAGAGGAGTTAGTCTTGAAGTCATGCGCCGGTTAAGGCTAAACTGAAAGG
ACAAGTTTTGGTGACTGCGCTCCTCCAAGCCAGTTACCTCGGTTCAAAGAGTTGGTAGCTCAGAGAACCTTCGAAAAACCGCC
CTGCAAGGCGGTTTTTTCGTTTTCAGAGCAAGAGATTACGCGCAGACCAAAACGATCTCAAGAAGATCATCTTATTAATCAGA
TAAAATATTTCTAGATTTCAGTGCAATTTATCTCTTCAAATGTAGCACCTGAAGTCAGCCCCATACGATATAAGTTGTAATTC
TCATGTTTGACAGCATTATCATCGATAAGCTTTAATGCGGTAGTTTATCACAGTTAAATTGCTAACGCAGTCAGGCACCGTGT
ATGAAATCTAACAATGCGCTCATCGTCATCCTCGGCACCGTCACCCTGGATGCTGTAGGCATAGGCTTGGTTATGCCGGTACT
GCCGGGCCTCTTGCGGGATGGCCACGATGCGTCCGGCGTAGAGGATCTGCTCATGTTTGACAGCTTATCATCGATGCATAATG
TGCCTGTCAAATGGACGAAGCAGGGATTCTGCAAACCCTATGCTACTCCGTCAAGCCGTCAATTGTCTGATTCGTTACCAATT
ATGACAACTTGACGGCTACATCATTCACTTTTTCTTCACAACCGGCACGGAACTCGCTCGGGCTGGCCCCGGTGCATTTTTTA
AATACCCGCGAGAAATAGAGTTGATCGTCAAAACCAACATTGCGACCGACGGTGGCGATAGGCATCCGGGTGGTGCTCAAAAG
CAGCTTCGCCTGGTGATACGTTGGTCCTCGCGCCAGCTTAAGACGCTAATCCCTAACTGCTGGCGGAAAAGATGTGACAGACG
CGACGGCGACAAGCAAACATGCTGTGCGACGCTGGCGATATCAAAATTGCTGTCTGCCAGGTGATCGCTGATGTACTGACAAG
CCTCGCGTACCCGATTATCCATCGGTGGATGGAGCGACTCGTTAATCGCTTCCATGCGCCGCAGTAACAATTGCTCAAGCAGA
TTTATCGCCAGCAGCTCCGAATAGCGCCCTTCCCCTTGCCCGGCGTTAATGATTTGCCCAAACAGGTCGCTGAAATGCGGCTG
GTGCGCTTCATCCGGGCGAAAGAACCCCGTATTGGCAAATATTGACGGCCAGTTAAGCCATTCATGCCAGTAGGCGCGCGGAC
GAAAGTAAACCCACTGGTGATACCATTCGCGAGCCTCCGGATGACGACCGTAGTGATGAATCTCTCCTGGCGGGAACAGCAAA
ATATCACCCGGTCGGCAAACAAATTCTCGTCCCTGATTTTTCACCACCCCCTGACCGCGAATGGTGAGATTGAGAATATAACC
TTTCATTCCCAGCGGTCGGTCGATAAAAAAATCGAGATAACCGTTGGCCTCAATCGGCGTTAAACCCGCCACCAGATGGGCAT
TAAACGAGTATCCCGGCAGCAGGGGATCATTTTGCGCTTCAGCCATACTTTTCATACTCCCGCCATTCAGAG**AAGAAACCAAT**
TGTCCATATTGCATCAGACATTGCCGTCACTGCGTCTTTTACTGGCTCTTCTCGCTAACCAAAACCGGTAACCCCGCTTATTA
AAAGCATTCTGTAACAAAGCGGGACCAAAGCCATGACAAAAACGCGTAACAAAAGTGTCTATAATCACGGCAGAAAAGTCCAC
ATTGATTATTTGCACGGCGTCACACTTTGCTATGCCATAGCATTTTTATCCATAAGATTAGCGGATCCTACCTGACGCTTTTT
ATCGCAACTCTCTACTGTTTCTCCATACCCGTTTTTTTGGGCTAGAAATAATTTTGTTTAACTTTAAGAAGGAGATATACATA
TGGCATAGGTTATCAACACGTTTGATGGGGTTGCGGATTATCTTCAGACATATCATAAGCTACCTGATAATTACATTACAAAA
TCAGAAGCACAAGCCCTCGGCTGGGTGGCATCAAAAGGGAACCTTGCATAGGTCGCTCCGGGGAAAAGCATCGGCGGAGACAT
CTTCTCAAACAGGGAAGGCAAACTCCCGTAGAAAAGCGGACGAACATGGCGTGAAGCGGATATTAACTATACATCAGGCTTCA

GAAATTCAGACCGGATTCTTTACTCAAGCGACTGGCTGATTTACAAAACAACGGACCATTATCAGACCTTTACAAAAATCAGA
TAAGCATGCACCATTCCTTGCGGCGGCGGTGCTCAACGGCCTCAACCTACTACTGGGCTGCTTCGGC

DNA sequence of pGS1T-PylRS^{C6a}-PylT$^{m15}_{CUA}$ (GenBank: PX848771, Addgene: 251554):

The pGS1T-PylRS^{C6a}-PylT$^{m15}_{CUA}$ plasmid contains a p15A type origin of replication, a tetracycline resistance gene (TetR), and a PylT$^{m15}_{CUA}$ under control of a proK promoter.

PylRS^{C6a}; p15a ori; TetR; PylT$^{m15}_{CUA}$.

ATGGATAAAAAACCGCTGAACACCCTGATTAGCGCGACCGGCCTGTGGATGAGCCGCACCGGCACCATTCATAAAATTAAACA
TCATGAAGTGAGCCGCAGCAAAATTTATATTGAAATGGCGTGCGGCGATCATCTGGTGGTGAACAACAGCCGCAGCAGCCGCA
CCGCGCGCGCGCTGCGCCATCATAAATATCGCAAAACCTGCAAACGCTGCCGCGTGAGCGATGAAGATCTGAACAAATTTCTG
ACCAAAGCGAACGAAGATCAGACCAGCGTGAAAGTGAAAGTGGTGAGCGCGCCGACCCGCACCAAAAAAGCGATGCCGAAAAG
CGTGGCGCGCGCGCCGAAACCGCTGGAAAACACCGAAGCGGCGCAGGCGCAGCCGAGCGGCAGCAAATTTAGCCCGGCGATTC
CGGTGAGCACCCAGGAAAGCGTGAGCGTGCCGGCGAGCGTGAGCACCAGCATTAGCAGCATTAGCACCGGCGCGACCGCGAGC
GCGCTGGTGAAAGGCAACACCAACCCGATTACCAGCATGAGCGCGCCGGTGCAGGCGAGCGCGCCGGCGCTGACCAAAAGCCA
GACCGATCGCCTGGAAGTGCTGCTGAACCCGAAAGATGAAATTAGCCTGAACAGCGGCAAACCGTTTCGCGAACTGGAAAGCG
AACTGCTGAGCCGCCGCAAAAAAGATCTGCAGCAGATTTATGCGGAAGAACGCGAAAACTATCTGGGCAAACTGGAACGCGAA
ATTACCCGCTTTTTTGTGGATCGCGGCTTTCTGGAAATTAAAAGCCCGATTCTGATTCCGCTGGAATATATTGAACGCATGGG
CATTGATAACGATACCGAACTGAGCAAACAGATTTTTCGCGTGGATAAAAACTTTTGCCTGCGCCCGGATCTGCATCCGAACC
TGTATAACTATCTGCGCAAACTGGATCGCGCGCTGCCGGATCCGATTAAAATTTTTGAAATTGGCCCGTGCTATCGCAAAGAA
AGCGATGGCAAAGAACATCTGGAAGAATTTACCATGCTGGCATTTTGCCAGATGGGCAGCGGCTGCACCCGCGAAAACCTGGA
AAGCATTATTACCGATTTTCTGAACCATCTGGGCATTGATTTTAAAATTGTGGGCGATAGCTGCATGGTGTATGGCGATACCC
TGGATGTGATGCATGGCGATCTGGAACTGAGCAGTTGTGTTGTGGGCCCGCGCCCGCTGGATCGCGAATGGGGCATTGATAAA
CCGTGGATTGGCGCGGGCTTTGGCCTGGAACGCCTGCTGAAAGTGAAACATGATTTTAAAAACATTAAACGCGCGGCGCGCAG
CGAAAGCTATTATAACGGCATTAGCACCAACCTGTAAAGGTGGCACTTTTCGGGGCAGTTTCAAACGGGTACCATATGGGAAT
TCGAAGCTTGGGCCCGAACAAAAACTCATCTCAGAAGAGGATCTGAATAGCGCCGTCGACCATCATCATCATCATCATTGAGT
TTAAACGGTCTCCAGCTTGGCTGTTTTGGCGGATGAGAGAAGATTTTCAGCCTGATACAGATTAAATCAGAACGCAGAAGCGG
TCTGATAAAACAGAATTTGCCTGGCGGCAGTAGCGCGGTGGTCCCACCTGACCCCATGCCGAACTCAGAAGTGAAACGCCGTA
GCGCCGATGGTAGTGTGGGGTCTCCCCATGCGAGAGTAGGGAACTGCCAGGCATCAAATAAAACGAAAGGCTCAGTCGAAAGA
CTGGGCCTTTCGTTTTATCTGTTGTTTGTCGGTGAACGCTCTCCTGAGTAGGACAAATCCGCCGGGAGCTGTCCCTCCTGTTC
AGCTACTGACGGGGTGGTGCGTAACGGCAAAAGCACCGCCGGACATCAGCGCTAGCGGAGTGTATACTGGCTTACTATGTTGG
CACTGATGAGGGTGTCAGTGAAGTGCTTCATGTGGCAGGAGAAAAAAGGCTGCACCGGTGCGTCAGCAGAATATGTGATACAG
GATATATTCCGCTTCCTCGCTCACTGACTCGCTACGCTCGGTCGTTCGACTGCGGCGAGCGGAAATGGCTTACGAACGGGGCG
GAGATTTCCTGGAAGATGCCAGGAAGATACTTAACAGGGAAGTGAGAGGGCCGCGGCAAAGCCGTTTTTCCATAGGCTCCGCC
CCCCTGACAAGCATCACGAAATCTGACGCTCAAATCAGTGGTGGCGAAACCCGACAGGACTATAAAGATACCAGGCGTTTCCC
CCTGGCGGCTCCCTCGTGCGCTCTCCTGTTCCTGCCTTTCGGTTTACCGGTGTCATTCCGCTGTTATGGCCGCGTTTGTCTCA
TTCCACGCCTGACACTCAGTTCCGGGTAGGCAGTTCGCTCCAAGCTGGACTGTATGCACGAACCCCCCGTTCAGTCCGACCGC
TGCGCCTTATCCGGTAACTATCGTCTTGAGTCCAACCCGGAAAGACATGCAAAAGCACCACTGGCAGCAGCCACTGGTAATTG
ATTTAGAGGAGTTAGTCTTGAAGTCATGCGCCGGTTAAGGCTAAACTGAAAGGACAAGTTTTGGTGACTGCGCTCCTCCAAGC
CAGTTACCTCGGTTCAAAGAGTTGGTAGCTCAGAGAACCTTCGAAAAACCGCCCTGCAAGGCGGTTTTTTCGTTTTCAGAGCA
AGAGATTACGCGCAGACCAAAACGATCTCAAGAAGATCATCTTATTAATCAGATAAAATATTTCTAGATTTCAGTGCAATTTA
TCTCTTCAAATGTAGCACCTGAAGTCAGCCCCATACGATATAAGTTGTAATTCTCATGTTGATCGGCACGTAATAGCGGGGCG
TAAATGGAAGCCGGCGGCGCTGGTAGCAAAATTCCTCGACGAACGTGGGATCGTAGTAGAGAAAACCGGCCCTTATAACCTGC
TGTTTCTCTTTAGTATTGGCATCGATAAAACCAAAGCAATGGGATTATTGCGTGGGTTGACGGAATTCAAACGCTCTTACGAT
CTCAACCTGCGGATCAAAAATATGCTACCCGATCTCTATGCAGAAGATCCCGATTTCTACCGCAATATGCGTATTCAGGATCT
GGCACAAGGGATCCATAAGCTGATTCGTAAACACGATCTTCCCGGTTTGATGTTGCGGGCATTCGATACTTTGCCGGAGATGA
TCATGACGCCACATCAGGCATGGCAACGACAAATTAAAGGCGAAGTAGAAACCATTGCGCTGGAACAACTGGTCGGTAGAGTA
TCGGCAAATATGATCCTGCCTTATCCACCGGGCGTACCGCTGTTGATGCCTGGAGAAATGCTGACCAAAGAGAGCCGCACAGT
ACTCGATTTTCTACTGATGCTTTGTTCCGTCGGGCAACATTACCCCGGTTTTGAAACGGATATTCACGGCGCGAAACAGGACG
AAGACGGCGTTTACCGCGTACGAGTCCTAAAAATGGCGGGATAACTTGCCAGAGCGGCTTCCATTTACGCCCCGCCCTGTTGA
CAGCTTATCATCGATAAGCTTTAATGCGGTAGTTTATCACAGTTAAATTGCTAACGCAGTCAGGCACCGTGTATGAAATCTAA
CAATGCGCTCATCGTCATCCTCGGCACCGTCACCCTGGATGCTGTAGGCATAGGCTTGGTTATGCCGGTACTGCCGGGCCTCT
TGCGGGATATCGTCCATTCCGACAGCATCGCCAGTCACTATGGCGTGCTGCTAGCGCTATATGCGTTGATGCAATTTCTATGC
GCACCCGTTCTCGGAGCACTGTCCGACCGCTTTGGCCGCCGCCCAGTCCTGCTCGCTTCCCTACTTGGAGCCACTATCGACTA
CGCGATCATGGCGACCACACCCGTCCTGTGGATCCTCTACGCCGGACGCATCGTGGCCGGCATCACCGGCGCCACAGGTGCGG
TTGCTGGCGCCTATATCGCCGACATCACCGATGGGGAAGATCGGGCTCGCCACTTCGGGCTCATGAGCGCTTGTTTCGGCGTG
GGTATGGTGGCAGGCCCCGTGGCCGGGGGACTGTTGGGCGCCATCTCCTTGCATGCACCATTCCTTGCGGCGGCGGTGCTCAA
CGGCCTCAACCTACTACTGGGCTGCTTCCTAATGCAGGAGTCCCATAAGGGAGAGCGTCGACCGATGCCCTTGAGAGCCTTCA
ACCCAGTCAGCTCCTTCCGGTGGGCGCGGGGCATGACTATCGTCGCCGCACTTATGACTGTCTTCTTTATCATGCAACTCGTA
GGACAGGTGCCGGCAGCGCTCTGGGTCATTTTCGGCGAGGACCGCTTTCGCTGGAGCGCGACGATGATCGGCCTGTCCCTTGC
GGTATTCGGAATCTTGCACGCCCTCGCTCAAGCCTTCGTCACTGGTCCCGCCACCAAACGTTTCGGCGAGAAGCAGGCCATTA
TCGCCGGCATGGCGGCCGACGCGCTGGGCTACGTCTTGCTGGCGTTCGCGACGCGAGGCTGGATGGCCTTCCCCATTATGATT
CTTCTCGCTTCCGGCGGCATCGGGATGCCCGCGTTGCAGGCCATGCTGTCCAGGCAGGTAGATGACGACCATCAGGGACAGCT
TCAAGGATCCCTCGCGGCTCTTACCAGCCTAACTTCCATCATTGGACCGCTGATCGTCACGGCGATTTATGCCGCCTCCGCGA

GCACATGGAACGGGTTGGCATGGATTGTAGGCGCCGCCCTATACCTTGTCTGCCTCCCCGCGTTGCGTCGCGGTGCATGGAGC
CGGGCCACCTCCACCTGAAGGGCGGGGCGTAAATGGAAGCCGGCGGCACCTCGCTAACGGATTCACCACTCCAAGAATTGGAG
CCAATCAATTCTTGCGGAGAACTGTGAATGCGCAAACCAACCCTTGGCAGAACATATCCATCGCGTCCGCCATCTCCAGCAGC
CGCACGCGGCGCATCTCGGGCTCCTTGCATGCACCATTCCTTGCGGCGGCGGTGCTCAACGGCCTCAACCTACTACTGGGCTG
CTTCCTAATGCAGGAGTCGCATAAGGGAGAGCGTCTGGCGAAAAAAAAGCCTGCTCGTTGAGCAGGCTTTTCGAATT**TGGCGG**
AAACCCCGGGAATCGAACCCGGCTGAACGGATTTAGAGTCCGTTCGGTCTCCCTGACCAGGTTTCCAATGCGGGGCGCATCTT
ACTGCGCAGATACGCCCTCGTCAATCCCTTAATAGCAAAATGCCTCCTGCACCATTATGTTCCGGATCTGCATCGCAGGATGC
TGCTGGCTACCCTGTGGAACACCTACATCTGTATTAACGAAGCGCTAACCGTTTTTATCATGCTCTGGGAGGCAGAATAAATG
ATCATATCGTCAATTATTACCTCCACGGGGAGAGCCTGAGCAAACTGGCCTCAGGCATTTGAGAAGCACACGGTCACACTGCT
TCCGGTAGTCAATAAACCGGTAAACCAGCAATAGACATAAGCGGCTATTTAACGAGAACGACCGAGCGCAGCGAGTCAGTGAG
CGAGGAAGCGGAAGAGCGAACTTTTGCTGAGTTGAAGGATCCTCGGGTTGTCAGCCTGTCCCGCTTATAAGATCATACGCCGT
TATACGTTGTTTACGCTTTGAGGAATCCCAT

DNA sequence of pGS1T-PylRS^{C6a}-*Mm*tRNA$^{Pyl}_{CUA}$ (GenBank: PX848772, Addgene: 251555):

The pGS1T-PylRS^{C6a}-*Mm*tRNA$^{Pyl}_{CUA}$ plasmid contains a p15A type origin of replication, a tetracycline resistance gene (TetR), and a PylT$^{m15}_{CUA}$ under control of a proK promoter.

PylRS^{C6a}; p15a ori; TetR; *Mm*tRNA^{Pyl}CUA.

ATGGATAAAAAACCGCTGAACACCCTGATTAGCGCGACCGGCCTGTGGATGAGCCGCACCGGCACCATTCATAAAATTAAACA
TCATGAAGTGAGCCGCAGCAAAATTTATATTGAAATGGCGTGCGGCGATCATCTGGTGGTGAACAACAGCCGCAGCAGCCGCA
CCGCGCGCGCGCTGCGCCATCATAAATATCGCAAAACCTGCAAACGCTGCCGCGTGAGCGATGAAGATCTGAACAAATTTCTG
ACCAAAGCGAACGAAGATCAGACCAGCGTGAAAGTGAAAGTGGTGAGCGCGCCGACCCGCACCAAAAAAGCGATGCCGAAAAG
CGTGGCGCGCGCGCCGAAACCGCTGGAAAACACCGAAGCGGCGCAGGCGCAGCCGAGCGGCAGCAAATTTAGCCCGGCGATTC
CGGTGAGCACCCAGGAAAGCGTGAGCGTGCCGGCGAGCGTGAGCACCAGCATTAGCAGCATTAGCACCGGCGCGACCGCGAGC
GCGCTGGTGAAAGGCAACACCAACCCGATTACCAGCATGAGCGCGCCGGTGCAGGCGAGCGCGCCGGCGCTGACCAAAAGCCA
GACCGATCGCCTGGAAGTGCTGCTGAACCCGAAAGATGAAATTAGCCTGAACAGCGGCAAACCGTTTCGCGAACTGGAAAGCG
AACTGCTGAGCCGCCGCAAAAAAGATCTGCAGCAGATTTATGCGGAAGAACGCGAAAACTATCTGGGCAAACTGGAACGCGAA
ATTACCCGCTTTTTTGTGGATCGCGGCTTTCTGGAAATTAAAAGCCCGATTCTGATTCCGCTGGAATATATTGAACGCATGGG
CATTGATAACGATACCGAACTGAGCAAACAGATTTTTCGCGTGGATAAAAACTTTTGCCTGCGCCCGGATCTGCATCCGAACC
TGTATAACTATCTGCGCAAACTGGATCGCGCGCTGCCGGATCCGATTAAAATTTTTGAAATTGGCCCGTGCTATCGCAAAGAA
AGCGATGGCAAAGAACATCTGGAAGAATTTACCATGCTGGCATTTTGCCAGATGGGCAGCGGCTGCACCCGCGAAAACCTGGA
AAGCATTATTACCGATTTTCTGAACCATCTGGGCATTGATTTTAAAATTGTGGGCGATAGCTGCATGGTGTATGGCGATACCC
TGGATGTGATGCATGGCGATCTGGAACTGAGCAGTTGTGTTGTGGGCCCGCGCCCGCTGGATCGCGAATGGGGCATTGATAAA
CCGTGGATTGGCGCGGGCTTTGGCCTGGAACGCCTGCTGAAAGTGAAACATGATTTTAAAAACATTAAACGCGCGGCGCGCAG
CGAAAGCTATTATAACGGCATTAGCACCAACCTGTAAAGGTGGCACTTTTCGGGGCAGTTTCAAACGGGTACCATATGGGAAT
TCGAAGCTTGGGCCCGAACAAAAACTCATCTCAGAAGAGGATCTGAATAGCGCCGTCGACCATCATCATCATCATCATTGAGT
TTAAACGGTCTCCAGCTTGGCTGTTTTGGCGGATGAGAGAAGATTTTCAGCCTGATACAGATTAAATCAGAACGCAGAAGCGG
TCTGATAAAACAGAATTTGCCTGGCGGCAGTAGCGCGGTGGTCCCACCTGACCCCATGCCGAACTCAGAAGTGAAACGCCGTA
GCGCCGATGGTAGTGTGGGGTCTCCCCATGCGAGAGTAGGGAACTGCCAGGCATCAAATAAAACGAAAGGCTCAGTCGAAAGA
CTGGGCCTTTCGTTTTATCTGTTGTTTGTCGGTGAACGCTCTCCTGAGTAGGACAAATCCGCCGGGAGCTGTCCCTCCTGTTC
AGCTACTGACGGGGTGGTGCGTAACGGCAAAAGCACCGCCGGACATCAGCGCTAGCGGAGTGTATACTGGCTTACTATGTTGG
CACTGATGAGGGTGTCAGTGAAGTGCTTCATGTGGCAGGAGAAAAAAGGCTGCACCGGTGCGTCAGCAGAATATGTGATACAG
GATATATTCCGCTTCCTCGCTCACTGACTCGCTACGCTCGGTCGTTCGACTGCGGCGAGCGGAAATGGCTTACGAACGGGGCG
GAGATTTCCTGGAAGATGCCAGGAAGATACTTAACAGGGAAGTGAGAGGGCCGCGGCAAAGCCGTT**TTTCCATAGGCTCCGCC**
CCCCTGACAAGCATCACGAAATCTGACGCTCAAATCAGTGGTGGCGAAACCCGACAGGACTATAAAGATACCAGGCGTTTCCC
CCTGGCGGCTCCCTCGTGCGCTCTCCTGTTCCTGCCTTTCGGTTTACCGGTGTCATTCCGCTGTTATGGCCGCGTTTGTCTCA
TTCCACGCCTGACACTCAGTTCCGGGTAGGCAGTTCGCTCCAAGCTGGACTGTATGCACGAACCCCCCGTTCAGTCCGACCGC
TGCGCCTTATCCGGTAACTATCGTCTTGAGTCCAACCCGGAAAGACATGCAAAAGCACCACTGGCAGCAGCCACTGGTAATTG
ATTTAGAGGAGTTAGTCTTGAAGTCATGCGCCGGTTAAGGCTAAACTGAAAGGACAAGTTTTGGTGACTGCGCTCCTCCAAGC
CAGTTACCTCGGTTCAAAGAGTTGGTAGCTCAGAGAACCTTCGAAAAACCGCCCTGCAAGGCGGTTTTTTCGTTTTCAGAGCA
AGAGATTACGCGCAGACCAAAACGATCTCAAGAAGATCATCTTATTAATCAGATAAAATATTTCTAGATTTCAGTGCAATTTA
TCTCTTCAAATGTAGCACCTGAAGTCAGCCCCATACGATATAAGTTGTAATTCTCATGTTGATCGGCACGTAATAGCGGGGCG
TAAATGGAAGCCGGCGGCGCTGGTAGCAAAATTCCTCGACGAACGTGGGATCGTAGTAGAGAAAACCGGCCCTTATAACCTGC
TGTTTCTCTTTAGTATTGGCATCGATAAAACCAAAGCAATGGGATTATTGCGTGGGTTGACGGAATTCAAACGCTCTTACGAT
CTCAACCTGCGGATCAAAAATATGCTACCCGATCTCTATGCAGAAGATCCCGATTTCTACCGCAATATGCGTATTCAGGATCT
GGCACAAGGGATCCATAAGCTGATTCGTAAACACGATCTTCCCGGTTTGATGTTGCGGGCATTCGATACTTTGCCGGAGATGA
TCATGACGCCACATCAGGCATGGCAACGACAAATTAAAGGCGAAGTAGAAACCATTGCGCTGGAACAACTGGTCGGTAGAGTA
TCGGCAAATATGATCCTGCCTTATCCACCGGGCGTACCGCTGTTGATGCCTGGAGAAATGCTGACCAAAGAGAGCCGCACAGT
ACTCGATTTTCTACTGATGCTTTGTTCCGTCGGGCAACATTACCCCGGTTTTGAAACGGATATTCACGGCGCGAAACAGGACG
AAGACGGCGTTTACCGCGTACGAGTCCTAAAAATGGCGGGATAACTTGCCAGAGCGGCTTCCATTTACGCCCCGCCCTGTTGA
CAGCTTATCA**TCGATAAGCTTTAATGCGGTAGTTTATCACAGTTAAATTGCTAACGCAGTCAGGCACCGTGTATGAAATCTAA**
CAATGCGCTCATCGTCATCCTCGGCACCGTCACCCTGGATGCTGTAGGCATAGGCTTGGTTATGCCGGTACTGCCGGGCCTCT
TGCGGGATATCGTCCATTCCGACAGCATCGCCAGTCACTATGGCGTGCTGCTAGCGCTATATGCGTTGATGCAATTTCTATGC
GCACCCGTTCTCGGAGCACTGTCCGACCGCTTTGGCCGCCGCCCAGTCCTGCTCGCTTCCCTACTTGGAGCCACTATCGACTA
CGCGATCATGGCGACCACACCCGTCCTGTGGATCCTCTACGCCGGACGCATCGTGGCCGGCATCACCGGCGCCACAGGTGCGG
TTGCTGGCGCCTATATCGCCGACATCACCGATGGGGAAGATCGGGCTCGCCACTTCGGGCTCATGAGCGCTTGTTTCGGCGTG

GGTATGGTGGCAGGCCCCGTGGCCGGGGGACTGTTGGGCGCCATCTCCTTGCATGCACCATTCCTTGCGGCGGCGGTGCTCAA
CGGCCTCAACCTACTACTGGGCTGCTTCCTAATGCAGGAGTCCCATAAGGGAGAGCGTCGACCGATGCCCTTGAGAGCCTTCA
ACCCAGTCAGCTCCTTCCGGTGGGCGCGGGGCATGACTATCGTCGCCGCACTTATGACTGTCTTCTTTATCATGCAACTCGTA
GGACAGGTGCCGGCAGCGCTCTGGGTCATTTTCGGCGAGGACCGCTTTCGCTGGAGCGCGACGATGATCGGCCTGTCCCTTGC
GGTATTCGGAATCTTGCACGCCCTCGCTCAAGCCTTCGTCACTGGTCCCGCCACCAAACGTTTCGGCGAGAAGCAGGCCATTA
TCGCCGGCATGGCGGCCGACGCGCTGGGCTACGTCTTGCTGGCGTTCGCGACGCGAGGCTGGATGGCCTTCCCCATTATGATT
CTTCTCGCTTCCGGCGGCATCGGGATGCCCGCGTTGCAGGCCATGCTGTCCAGGCAGGTAGATGACGACCATCAGGGACAGCT
TCAAGGATCCCTCGCGGCTCTTACCAGCCTAACTTCCATCATTGGACCGCTGATCGTCACGGCGATTTATGCCGCCTCCGCGA
GCACATGGAACGGGTTGGCATGGATTGTAGGCGCCGCCCTATACCTTGTCTGCCTCCCCGCGTTGCGTCGCGGTGCATGGAGC
CGGGCCACCTCCACCTGAAGGGCGGGGCGTAAATGGAAGCCGGCGGCACCTCGCTAACGGATTCACCACTCCAAGAATTGGAG
CCAATCAATTCTTGCGGAGAACTGTGAATGCGCAAACCAACCCTTGGCAGAACATATCCATCGCGTCCGCCATCTCCAGCAGC
CGCACGCGGCGCATCTCGGGCTCCTTGCATGCACCATTCCTTGCGGCGGCGGTGCTCAACGGCCTCAACCTACTACTGGGCTG
CTTCCTAATGCAGGAGTCGCATAAGGGAGAGCGTCTGGCGAAAAAAAAGCCTGCTCGTTGAGCAGGCTTTTCGAATTTGGCGG
AAACCCCGGGAATCTAACCCGGCTGAACGGATTTAGAGTCCATTCGATCTACATGATCAGGTTTCCAATGCGGGGCGCATCTT
ACTGCGCAGATACGCCCTCGTCAATCCCTTAATAGCAAAATGCCTCCTGCACCATTATGTTCCGGATCTGCATCGCAGGATGC
TGCTGGCTACCCTGTGGAACACCTACATCTGTATTAACGAAGCGCTAACCGTTTTTATCATGCTCTGGGAGGCAGAATAAATG
ATCATATCGTCAATTATTACCTCCACGGGGAGAGCCTGAGCAAACTGGCCTCAGGCATTTGAGAAGCACACGGTCACACTGCT
TCCGGTAGTCAATAAACCGGTAAACCAGCAATAGACATAAGCGGCTATTTAACGAGAACGACCGAGCGCAGCGAGTCAGTGAG
CGAGGAAGCGGAAGAGCGAACTTTTGCTGAGTTGAAGGATCCTCGGGTTGTCAGCCTGTCCCGCTTATAAGATCATACGCCGT
TATACGTTGTTTACGCTTTGAGGAATCCCAT

DNA sequence of pBAD-sfGFP150$_{TAG}$ (GenBank: PX848777):

The pBAD-sfGFP150$_{TAG}$ plasmid contains a pBR322 type origin of replication, a kanamycin resistance gene (KanR), an araC element and an sfGFP150$_{TAG}$ under control of an araBAD promoter. Modified from Addgene #85483 (replacement of antibiotic resistance cassette)[93].

araBAD; **sfGFP150$_{TAG}$-6xHis**; **KanR**; **pBR322 ori**; **araC**.

AAGAAACCAATTGTCCATATTGCATCAGACATTGCCGTCACTGCGTCTTTTACTGGCTCTTCTCGCTAACCAAACCGGTAACC
CCGCTTATTAAAAGCATTCTGTAACAAAGCGGGACCAAAGCCATGACAAAAACGCGTAACAAAAGTGTCTATAATCACGGCAG
AAAAGTCCACATTGATTATTTGCACGGCGTCACACTTTGCTATGCCATAGCATTTTTATCCATAAGATTAGCGGATCCTACCT
GACGCTTTTTATCGCAACTCTCTACTGTTTCTCCATACCCGTTTTTTTGGGCTAACAGGAGGAATTAACCATGGTTAGCAAAGG
TGAAGAACTGTTTACCGGCGTTGTGCCGATTCTGGTGGAACTGGATGGTGATGTGAATGGCCATAAATTTAGCGTTCGTGGCG
AAGGCGAAGGTGATGCGACCAACGGTAAACTGACCCTGAAATTTATTTGCACCACCGGTAAACTGCCGGTTCCGTGGCCGACC
CTGGTGACCACCCTGACCTATGGCGTTCAGTGCTTTAGCCGCTATCCGGATCATATGAAACGCCATGATTTCTTTAAAAGCGC
GATGCCGGAAGGCTATGTGCAGGAACGTACCATTAGCTTCAAAGATGATGGCACCTATAAAACCCGTGCGGAAGTTAAATTTG
AAGGCGATACCCTGGTGAACCGCATTGAACTGAAAGGTATTGATTTTAAAGAAGATGGCAACATTCTGGGTCATAAACTGGAA
TATAATTTCAACAGCCATTAGGTGTATATTACCGCCGATAAACAGAAAAATGGCATCAAAGCGAACTTTAAAATCCGTCACAA
CGTGGAAGATGGTAGCGTGCAGCTGGCGGATCATTATCAGCAGAATACCCCGATTGGTGATGGCCCGGTGCTGCTGCCGGATA
ATCATTATCTGAGCACCCAGAGCGTTCTGAGCAAAGATCCGAATGAAAAACGTGATCATATGGTGCTGCTGGAATTTGTTACC
GCCGCGGGCATTACCCACGGTATGGATGAACTGTATAAAGGCAGCCACCATCATCATCACCATTAAAGCTCGAGATCTGCAGC
TGGTACCATATGGGAATTCGAAGCTTGGCTGTTTTGGCGGATGAGAGAAGATTTTCAGCCTGATACAGATTAAATCAGAACGC
AGAAGCGGTCTGATAAAACAGAATTTGCCTGGCGGCAGTAGCGCGGTGGTCCCACCTGACCCCATGCCGAACTCAGAAGTGAA
ACGCCGTAGCGCCGATGGTAGTGTGGGGTCTCCCCATGCGAGAGTAGGGAACTGCCAGGCATCAAATAAAACGAAAGGCTCAG
TCGAAAGACTGGGCCTTTCGTTTTATCTGTTGTTTGTCGGTGAACGCTCTCCTGAGTAGGACAAATCCGCCGGGAGCGGATTT
GAACGTTGCGAAGCAACGGCCCGGAGGGTGGCGGGCAGGACGCCCGCCATAAACTGCCAGGCATCAAATTAAGCAGAAGGCCA
TCCTGACGGATGGCCTTTTTGCGTTTCTACAAACTCTTGTGCGCGGAACCCCTATTTGTTTATTTTTCTAAATACATTCAAAT
ATGTATCCGCTCATGAATTAATTCTTAGAAAAACTCATCGAGCATCAAATGAAACTGCAATTTATTCATATCAGGATTATCAA
TACCATATTTTTGAAAAAGCCGTTTCTGTAATGAAGGAGAAAACTCACCGAGGCAGTTCCATAGGATGGCAAGATCCTGGTAT
CGGTCTGCGATTCCGACTCGTCCAACATCAATACAACCTATTAATTTCCCCTCGTCAAAAATAAGGTTATCAAGTGAGAAATC
ACCATGAGTGACGACTGAATCCGGTGAGAATGGCAAAAGTTTATGCATTTCTTTCCAGACTTGTTCAACAGGCCAGCCATTAC
GCTCGTCATCAAAATCACTCGCATCAACCAAACCGTTATTCATTCGTGATTGCGCCTGAGCGAGACGAAATACGCGATCGCTG
TTAAAAGGACAATTACAAACAGGAATCGAATGCAACCGGCGCAGGAACACTGCCAGCGCATCAACAATATTTTCACCTGAATC
AGGATATTCTTCTAATACCTGGAATGCTGTTTTCCCGGGGATCGCAGTGGTGAGTAACCATGCATCATCAGGAGTACGGATAA
AATGCTTGATGGTCGGAAGAGGCATAAATTCCGTCAGCCAGTTTAGTCTGACCATCTCATCTGTAACATCATTGGCAACGCTA
CCTTTGCCATGTTTCAGAAACAACTCTGGCGCATCGGGCTTCCCATACAATCGATAGATTGTCGCACCTGATTGCCCGACATT
ATCGCGAGCCCATTTATACCCATATAAATCAGCATCCATGTTGGAATTTAATCGCGGCCTAGAGCAAGACGTTTCCCGTTGAA
TATGGCTCATAACACCCCTTGTATTACTGTTTATGTAAGCAGACAGTTTTATTGTTCATGACCAAAATCCCTTAACGTGAGTT
TTCGTTCCACTGAGCGTCAGACCCCGTAGAAAAGATCAAAGGATCTTCTTGAGATCCTTTTTTTCTGCGCGTAATCTGCTGCT
TGCAAACAAAAAAACCACCGCTACCAGCGGTGGTTTGTTTGCCGGATCAAGAGCTACCAACTCTTTTTCCGAAGGTAACTGGC
TTCAGCAGAGCGCAGATACCAAATACTGTCCTTCTAGTGTAGCCGTAGTTAGGCCACCACTTCAAGAACTCTGTAGCACCGCC
TACATACCTCGCTCTGCTAATCCTGTTACCAGTGGCTGCTGCCAGTGGCGATAAGTCGTGTCTTACCGGGTTGGACTCAAGAC
GATAGTTACCGGATAAGGCGCAGCGGTCGGGCTGAACGGGGGGTTCGTGCACACAGCCCAGCTTGGAGCGAACGACCTACACC
GAACTGAGATACCTACAGCGTGAGCTATGAGAAAGCGCCACGCTTCCCGAAGGGAGAAAGGCGGACAGGTATCCGGTAAGCGG
CAGGGTCGGAACAGGAGAGCGCACGAGGGAGCTTCCAGGGGGAAACGCCTGGTATCTTTATAGTCCTGTCGGGTTTCGCCACC
TCTGACTTGAGCGTCGATTTTTGTGATGCTCGTCAGGGGGGCGGAGCCTATGGAAAAACGCCAGCAACGCGGCCTTTTTACGG
TTCCTGGCCTTTTGCTGGCCTTTTGCTCACATGTTCTTTCCTGCGTTATCCCCTGATTCTGTGGATAACCGTATTACCGCCTT

TGAGTGAGCTGATACCGCTCGCCGCAGCCGAACGACCGAGCGCAGCGAGTCAGTGAGCGAGGAAGCGGAAGAGCGCCTGATGC
GGTATTTTCTCCTTACGCATCTGTGCGGTATTTCACACCGCATATGGTGCACTCTCAGTACAATCTGCTCTGATGCCGCATAG
TTAAGCCAGTATACACTCCGCTATCGCTACGTGACTGGGTCATGGCTGCGCCCCGACACCCGCCAACACCCGCTGACGCGCCC
TGACGGGCTTGTCTGCTCCCGGCATCCGCTTACAGACAAGCTGTGACCGTCTCCGGGAGCTGCATGTGTCAGAGGTTTTCACC
GTCATCACCGAAACGCGCGAGGCAGCAGATCAATTCGCGCGCGAAGGCGAAGCGGCATGCATAATGTGCCTGTCAAATGGACG
AAGCAGGGATTCTGCAAACCCTATGCTACTCCGTCAAGCCGTCAATTGTCTGATTCGTTACCAATTATGACAACTTGACGGCT
ACATCATTCACTTTTTCTTCACAACCGGCACGGAACTCGCTCGGGCTGGCCCCGGTGCATTTTTTAAATACCCGCGAGAAATA
GAGTTGATCGTCAAAACCAACATTGCGACCGACGGTGGCGATAGGCATCCGGGTGGTGCTCAAAAGCAGCTTCGCCTGGCTGA
TACGTTGGTCCTCGCGCCAGCTTAAGACGCTAATCCCTAACTGCTGGCGGAAAAGATGTGACAGACGCGACGGCGACAAGCAA
ACATGCTGTGCGACGCTGGCGATATCAAAATTGCTGTCTGCCAGGTGATCGCTGATGTACTGACAAGCCTCGCGTACCCGATT
ATCCATCGGTGGATGGAGCGACTCGTTAATCGCTTCCATGCGCCGCAGTAACAATTGCTCAAGCAGATTTATCGCCAGCAGCT
CCGAATAGCGCCCTTCCCCTTGCCCGGCGTTAATGATTTGCCCAAACAGGTCGCTGAAATGCGGCTGGTGCGCTTCATCCGGG
CGAAAGAACCCCGTATTGGCAAATATTGACGGCCAGTTAAGCCATTCATGCCAGTAGGCGCGCGGACGAAAGTAAACCCACTG
GTGATACCATTCGCGAGCCTCCGGATGACGACCGTAGTGATGAATCTCTCCTGGCGGGAACAGCAAAATATCACCCGGTCGGC
AAACAAATTCTCGTCCCTGATTTTTCACCACCCCCTGACCGCGAATGGTGAGATTGAGAATATAACCTTTCATTCCCAGCGGT
CGGTCGATAAAAAAATCGAGATAACCGTTGGCCTCAATCGGCGTTAAACCCGCCACCAGATGGGCATTAAACGAGTATCCCGG
CAGCAGGGGATCATTTTGCGCTTCAGCCATACTTTTCATACTCCCGCCATTCAGAG

DNA sequence of pET28(+)-SLAC-6xHis (GenBank: PX848773, Addgene: 251556):

The pET28(+)-SLAC-6xHis plasmid contains a pBR322 type origin of replication, a kanamycin resistance gene (KanR), an araC element and an $sfGFP150_{TAG}$ under control of an araBAD promoter.

SLAC-6xHis; **KanR**; **pBR322 ori**.

TAATACGACTCACTATAGGGGAATTGTGAGCGGATAACAATTCCCCTCTAGAAATAATTTTGTTTAACTTTAAGAAGGAGATA
TACCATGGCTCCTGCTGCAAAAGGTATTACTGCTCGTACTGCACCTGCTGGTGGCGAAGTGCGTCATCTCAAGATGTACGCTG
AAAAGCTGGCAGACGGTCAGATGGGCTACGGCTTCGAAAAGGGCAAGGCATCGGTCCCTGGCCCTCTGATCGAAGTCAACGAA
GGCGACACTCTGCATATCGAATTCACCAACACTATGGACGTGCGTGCTAGTCTGCATGTGCATGGCCTGGACTACGAAATCTC
CAGTGACGGTACCGCAATGAACAAATCAGATGTTGAACCTGGTGGTACTCGTACTTATACATGGCGTACTCATAAACCTGGTC
GTCGTGACGACGGCACCTGGCGTCCTGGCAGTGCAGGCTACTGGCATTACCATGACCATGTCGTCGGCACCGAACATGGCACC
GGAGGCATCCGTAACGGCCTGTACGGCCCTGTGATCGTGCGTCGTAAGGGTGACGTGCTGCCTGACGCTACTCATACTATCGT
CTTCAACGACATGACCATCAACAACCGTAAACCTCATACCGGCCCTGACTTCGAAGCTACCGTGGGCGACCGTGTGGAAATCG
TCATGATCACTCATGGTGAATACTACCATACCTTCCACATGCATGGTCATCGTTGGGCAGACAACCGTACCGGCATCCTCACC
GGCCCTGACGACCCTTCCCGTGTCATCGACAACAAGATCACCGGCCCTGCTGACTCCTTCGGCTTCCAGATCATCGCAGGTGA
AGGTGTGGGCGCTGGTGCATGGATGTACCATTGTCATGTCCAGAGTCATTCCGACATGGGCATGGTGGGTCTGTTCCTGGTGA
AGAAGCCTGACGGCACTATCCCTGGTTACGAACCTCATGAACATGGCGGTGCAACCGCAAAGAGTGGCGAAAGTGGTGAACCT
ACTGGCGGTGCAGCTGCACATGAACATGAACATCTCGAGCACCACCACCACCACCACTGAGATCCGGCTGCTAACAAAGCCCG
AAAGGAAGCTGAGTTGGCTGCTGCCACCGCTGAGCAATAACTAGCATAACCCCTTGGGGCCTCTAAACGGGTCTTGAGGGGTT
TTTTGCTGAAAGGAGGAACTATATCCGGATTGGCGAATGGGACGCGCCCTGTAGCGGCGCATTAAGCGCGGCGGGTGTGGTGG
TTACGCGCAGCGTGACCGCTACACTTGCCAGCGCCCTAGCGCCCGCTCCTTTCGCTTTCTTCCCTTCCTTTCTCGCCACGTTC
GCCGGCTTTCCCCGTCAAGCTCTAAATCGGGGGCTCCCTTTAGGGTTCCGATTTAGTGCTTTACGGCACCTCGACCCCAAAAA
ACTTGATTAGGGTGATGGTTCACGTAGTGGGCCATCGCCCTGATAGACGGTTTTTCGCCCTTTGACGTTGGAGTCCACGTTCT
TTAATAGTGGACTCTTGTTCCAAACTGGAACAACACTCAACCCTATCTCGGTCTATTCTTTTGATTTATAAGGGATTTTGCCG
ATTTCGGCCTATTGGTTAAAAAATGAGCTGATTTAACAAAAATTTAACGCGAATTTTAACAAAATATTAACGCTTACAATTTA
GGTGGCACTTTTCGGGGAAATGTGCGCGGAACCCCTATTTGTTTATTTTTCTAAATACATTCAAATATGTATCCGCTCATGAA
TTAATTCTTAGAAAAACTCATCGAGCATCAAATGAAACTGCAATTTATTCATATCAGGATTATCAATACCATATTTTTGAAAA
AGCCGTTTCTGTAATGAAGGAGAAAACTCACCGAGGCAGTTCCATAGGATGGCAAGATCCTGGTATCGGTCTGCGATTCCGAC
TCGTCCAACATCAATACAACCTATTAATTTCCCCTCGTCAAAAATAAGGTTATCAAGTGAGAAATCACCATGAGTGACGACTG
AATCCGGTGAGAATGGCAAAAGTTTATGCATTTCTTTCCAGACTTGTTCAACAGGCCAGCCATTACGCTCGTCATCAAAATCA
CTCGCATCAACCAAACCGTTATTCATTCGTGATTGCGCCTGAGCGAGACGAAATACGCGATCGCTGTTAAAAGGACAATTACA
AACAGGAATCGAATGCAACCGGCGCAGGAACACTGCCAGCGCATCAACAATATTTTCACCTGAATCAGGATATTCTTCTAATA
CCTGGAATGCTGTTTTCCCGGGGATCGCAGTGGTGAGTAACCATGCATCATCAGGAGTACGGATAAAATGCTTGATGGTCGGA
AGAGGCATAAATTCCGTCAGCCAGTTTAGTCTGACCATCTCATCTGTAACATCATTGGCAACGCTACCTTTGCCATGTTTCAG
AAACAACTCTGGCGCATCGGGCTTCCCATACAATCGATAGATTGTCGCACCTGATTGCCCGACATTATCGCGAGCCCATTTAT
ACCCATATAAATCAGCATCCATGTTGGAATTTAATCGCGGCCTAGAGCAAGACGTTTCCCGTTGAATATGGCTCATAACACCC
CTTGTATTACTGTTTATGTAAGCAGACAGTTTTATTGTTCATGACCAAAATCCCTTAACGTGAGTTTTCGTTCCACTGAGCGT
CAGACCCCGTAGAAAAGATCAAAGGATCTTCTTGAGATCCTTTTTTTCTGCGCGTAATCTGCTGCTTGCAAACAAAAAAACCA
CCGCTACCAGCGGTGGTTTGTTTGCCGGATCAAGAGCTACCAACTCTTTTTCCGAAGGTAACTGGCTTCAGCAGAGCGCAGAT
ACCAAATACTGTCCTTCTAGTGTAGCCGTAGTTAGGCCACCACTTCAAGAACTCTGTAGCACCGCCTACATACCTCGCTCTGC
TAATCCTGTTACCAGTGGCTGCTGCCAGTGGCGATAAGTCGTGTCTTACCGGGTTGGACTCAAGACGATAGTTACCGGATAAG
GCGCAGCGGTCGGGCTGAACGGGGGGTTCGTGCACACAGCCCAGCTTGGAGCGAACGACCTACACCGAACTGAGATACCTACA
GCGTGAGCTATGAGAAAGCGCCACGCTTCCCGAAGGGAGAAAGGCGGACAGGTATCCGGTAAGCGGCAGGGTCGGAACAGGAG
AGCGCACGAGGGAGCTTCCAGGGGGAAACGCCTGGTATCTTTATAGTCCTGTCGGGTTTCGCCACCTCTGACTTGAGCGTCGA
TTTTTGTGATGCTCGTCAGGGGGGCGGAGCCTATGGAAAAACGCCAGCAACGCGGCCTTTTTACGGTTCCTGGCCTTTTGCTG

GCCTTTTGCTCACATGTTCTTTCCTGCGTTATCCCCTGATTCTGTGGATAACCGTATTACCGCCTTTGAGTGAGCTGATACCG
CTCGCCGCAGCCGAACGACCGAGCGCAGCGAGTCAGTGAGCGAGGAAGCGGAAGAGCGCCTGATGCGGTATTTTCTCCTTACG
CATCTGTGCGGTATTTCACACCGCAATGGTGCACTCTCAGTACAATCTGCTCTGATGCCGCATAGTTAAGCCAGTATACACTC
CGCTATCGCTACGTGACTGGGTCATGGCTGCGCCCCGACACCCGCCAACACCCGCTGACGCGCCCTGACGGGCTTGTCTGCTC
CCGGCATCCGCTTACAGACAAGCTGTGACCGTCTCCGGGAGCTGCATGTGTCAGAGGTTTTCACCGTCATCACCGAAACGCGC
GAGGCAGCTGCGGTAAAGCTCATCAGCGTGGTCGTGAAGCGATTCACAGATGTCTGCCTGTTCATCCGCGTCCAGCTCGTTGA
GTTTCTCCAGAAGCGTTAATGTCTGGCTTCTGATAAAGCGGGCCATGTTAAGGGCGGTTTTTTCCTGTTTGGTCACTGATGCC
TCCGTGTAAGGGGGATTTCTGTTCATGGGGGTAATGATACCGATGAAACGAGAGAGGATGCTCACGATACGGGTTACTGATGA
TGAACATGCCCGGTTACTGGAACGTTGTGAGGGTAAACAACTGGCGGTATGGATGCGGCGGGACCAGAGAAAAATCACTCAGG
GTCAATGCCAGCGCTTCGTTAATACAGATGTAGGTGTTCCACAGGGTAGCCAGCAGCATCCTGCGATGCAGATCCGGAACATA
ATGGTGCAGGGCGCTGACTTCCGCGTTTCCAGACTTTACGAAACACGGAAACCGAAGACCATTCATGTTGTTGCTCAGGTCGC
AGACGTTTTGCAGCAGCAGTCGCTTCACGTTCGCTCGCGTATCGGTGATTCATTCTGCTAACCAGTAAGGCAACCCCGCCAGC
CTAGCCGGGTCCTCAACGACAGGAGCACGATCATGCGCACCCGTGGGGCCGCCATGCCGGCGATAATGGCCTGCTTCTCGCCG
AAACGTTTGGTGGCGGGACCAGTGACGAAGGCTTGAGCGAGGGCGTGCAAGATTCCGAATACCGCAAGCGACAGGCCGATCAT
CGTCGCGCTCCAGCGAAAGCGGTCCTCGCCGAAAATGACCCAGAGCGCTGCCGGCACCTGTCCTACGAGTTGCATGATAAAGA
AGACAGTCATAAGTGCGGCGACGATAGTCATGCCCCGCGCCCACCGGAAGGAGCTGACTGGGTTGAAGGCTCTCAAGGGCATC
GGTCGAGATCCCGGTGCCTAATGAGTGAGCTAACTTACATTAATTGCGTTGCGCTCACTGCCTGCTTTCCAGTCGGGAAACCT
GTCGTGCCAGCTGCATTAATGAATCGGCCAACGCGCGGGGAGAGGCGGTTTGCGTATTGGGCGCCAGGGTGGTTTTTCTTTTC
ACCAGTGAGACGGGCAACAGCTGATTGCCCTTCACCGCCTGGCCCTGAGAGAGTTGCAGCAAGCGGTCCACGCTGGTTTGCCC
CAGCAGGCGAAAATCCTGTTTGATGGTGGTTAACGGCGGGATATAACATGAGCTGTCTTCGGTATCGTCGTATCCCACTACCG
AGATATCCGCACCAACGCGCAGCCCGGACTCGGTAATGGCGCGCATTGCGCCCAGCGCCATCTGATCGTTGGCAACCAGCATC
GCAGTGGGAACGATGCCCTCATTCAGCATTTGCATGGTTTGTTGAAAACCGGACATGGCACTCCAGTCGCCTTCCCGTTCCGC
TATCGGCTGAATTTGATTGCGAGTGAGATATTTATGCCAGCCAGCCAGACGCAGACGCGCCGAGACAGAACTTAATGGGCCCG
CTAACAGCGCGATTTGCTGGTGACCCAATGCGACCAGATGCTCCACGCCCAGTCGCGTACCGTCTTCATGGGAGAAAATAATA
CTGTTGATGGGTGTCTGGTCAGAGACATCAAGAAATAACGCCGGAACATTAGTGCAGGCAGCTTCCACAGCAATGGCATCCTG
GTCATCCAGCGGATAGTTAATGATCAGCCCACTGACGCGTTGCGCGAGAAGATTGTGCACCGCCGCTTTACAGGCTTCGACGC
CGCTTCGTTCTACCATCGACACCACCACGCTGGCACCCAGTTGATCGGCGCGAGATTTAATCGCCGCGACAATTTGCGACGGC
GCGTGCAGGGCCAGACTGGAGGTGGCAACGCCAATCAGCAACGACTGTTTGCCCGCCAGTTGTTGTGCCACGCGGTTGGGAAT
GTAATTCAGCTCCGCCATCGCCGCTTCCACTTTTTCCCGCGTTTTCGCAGAAACGTGGCTGGCCTGGTTCACCACGCGGGAAA
CGGTCTGATAAGAGACACCGGCATACTCTGCGACATCGTATAACGTTACTGGTTTCACATTCACCACCCTGAATTGACTCTCT
TCCGGGCGCTATCATGCCATACCGCGAAAGGTTTTGCGCCATTCGATGGTGTCCGGGATCTCGACGCTCTCCCTTATGCGACT
CCTGCATTAGGAAGCAGCCCAGTAGTAGGTTGAGGCCGTTGAGCACCGCCGCCGCAAGGAATGGTGCATGCAAGGAGATGGCG
CCCAACAGTCCCCCGGCCACGGGGCCTGCCACCATACCCACGCCGAAACAAGCGCTCATGAGCCCGAAGTGGCGAGCCCGATC
TTCCCCATCGGTGATGTCGGCGATATAGGCGCCAGCAACCGCACCTGTGGCGCCGGTGATGCCGGCCACGATGCGTCCGGCGT
AGAGGATCGAGATCTCGATCCCGCGAAAT

VI. References

32. Jones, S. M. & Solomon, E. I. Electron transfer and reaction mechanism of laccases. *Cell. Mol. Life Sci.* **72**, 869–883 (2015).
42. Zovo, K. *et al.* Substitution of the Methionine Axial Ligand of the T1 Copper for the Fungal-like Phenylalanine Ligand (M298F) Causes Local Structural Perturbations that Lead to Thermal Instability and Reduced Catalytic Efficiency of the Small Laccase from Streptomyces coelicolor A3(2). *ACS Omega* **7**, 6184–6194 (2022).
55. Wang, J.-X. *et al.* Unexpected effect of an axial ligand mutation in the type 1 copper center in small laccase: structure-based analyses and engineering to increase reduction potential and activity. *Chem. Sci.* **16**, 11339–11346 (2025).
62. Olbrich, A. C. *et al.* Substitution of the Axial Type 1 Cu Ligand Affords Binding of a Water Molecule in Axial Position Affecting Kinetics, Spectral, and Structural Properties of the Small Laccase Ssl1. *Chem. – Eur. J.* **31**, e202403005 (2025).
74. Pavitt, A. S., Bylaska, E. J. & Tratnyek, P. G. Oxidation potentials of phenols and anilines: correlation analysis of electrochemical and theoretical values. *Environ. Sci. Process. Impacts* **19**, 339–349 (2017).
93. Miyake-Stoner, S. J. *et al.* Generating Permissive Site-Specific Unnatural Aminoacyl-tRNA Synthetases. *Biochemistry* **49**, 1667–1677 (2010).
116. IUPAC. Redox indicators. Characteristics and applications. *Pure Appl. Chem.* **50**, 463–498 (1978).
117. Xue, X. *et al.* Benzoxazinone-containing 3,5-dimethylisoxazole derivatives as BET bromodomain inhibitors for treatment of castration-resistant prostate cancer. *Eur. J. Med. Chem.* **152**, 542–559 (2018).
