# Supplementary material for: Hydrophobic tuning with non-canonical amino acids in a copper metalloenzyme
Source: Nat Chem. 2026 Apr 13;18(7):1269–77. doi: 10.1038/s41557-026-02116-7 (PMC13323077; doi:10.1038/s41557-026-02116-7)
Supplement: Supplementary file 2 — Reporting Summary [file 41557_2026_2116_MOESM2_ESM.pdf]

nature portfolio

Corresponding author(s): Alexandria Deliz Liang

Last updated by author(s): Jan 10, 2025

Reporting Summary

Nature Portfolio wishes to improve the reproducibility of the work that we publish. This form provides structure for consistency and transparency in reporting. For further information on Nature Portfolio policies, see our Editorial Policies and the Editorial Policy Checklist.

Statistics

For all statistical analyses, confirm that the following items are present in the figure legend, table legend, main text, or Methods section.

n/a	Confirmed	
☐	☒	The exact sample size (*n*) for each experimental group/condition, given as a discrete number and unit of measurement
☐	☒	A statement on whether measurements were taken from distinct samples or whether the same sample was measured repeatedly
☐	☒	The statistical test(s) used AND whether they are one- or two-sided<br>*Only common tests should be described solely by name; describe more complex techniques in the Methods section.*
☐	☒	A description of all covariates tested
☒	☐	A description of any assumptions or corrections, such as tests of normality and adjustment for multiple comparisons
☐	☒	A full description of the statistical parameters including central tendency (e.g. means) or other basic estimates (e.g. regression coefficient) AND variation (e.g. standard deviation) or associated estimates of uncertainty (e.g. confidence intervals)
☐	☒	For null hypothesis testing, the test statistic (e.g. F, t, r) with confidence intervals, effect sizes, degrees of freedom and P value noted<br>*Give P values as exact values whenever suitable.*
☒	☐	For Bayesian analysis, information on the choice of priors and Markov chain Monte Carlo settings
☒	☐	For hierarchical and complex designs, identification of the appropriate level for tests and full reporting of outcomes
☐	☒	Estimates of effect sizes (e.g. Cohen's d, Pearson's r), indicating how they were calculated

Our web collection on statistics for biologists contains articles on many of the points above.

Software and code

Policy information about availability of computer code

Data collection	Fluorescence and absorbance data for amber suppression testing and kinetic assays were collected using i-control 2.0 software. Absorbance data for redox potential determination were collected using Cary WinUV Scan Application Version 5.0.0.999. UV-VIS data were collected using either Cary WinUV Scan Application Version 5.0.0.999 or SkanIt RE 6.0.1 software. AAS data were collected using SpectrAA Worksheet AA Software Version 5.2 PRO. UPLC data for phenolic substrates were collected using OpenLab CDS Version 2.7. NMR data was collected using Topspin 4.1.3. Protein concentrations were collected using the NanoDrop 2000/2000c operation software version 1.6.198. LC-MS and UPLC data were collected using the OpenLab CDS Version 2.7 software. EPR was collected on a Elexsys E500 EPR spectrometer (Bruker Biospin, Rheinstetten, Germany) equipped with an SHQ resonator (Bruker) and an ESR900 Helium flow cryostat (Oxford Instruments, Oxfordshire, UK) running Xepr (Bruker).
Data analysis	Data plotting was carried out in GraphPadPrism Version 10. Data analysis and fitting was performed with GraphPadPrism Version 10, except in the case of the spectral deconvolution, calibration curves for phenol oxidation, and PDB analysis for laccases, which were analyzed with SciPy curvefit in Python, Microsoft Excel 16.104 , and a custom Python code, respectively (see below). Integration of UPLC UV peaks was carried out in OpenLab CDS Version 2.7. LC-MS/MS data were processed in Byonic 5.4 software. Crystallographic data were processed with Coot and phenix.refine and depicted with PyMOL Molecular Graphics System Version 2.5.0. NMR data was analyzed with MestreNova 14.1.2. EPR data was analyzed in MATLAB 2017a with EasySpin (version 6.0.8).<br><br>The code and input data used for determining calculated logP, deconvolution of redox titration spectra, and comparison of T1Cu environments of bacterial and fungal laccases are available on Zenodo (10.5281/zenodo.18271154).

For manuscripts utilizing custom algorithms or software that are central to the research but not yet described in published literature, software must be made available to editors and reviewers. We strongly encourage code deposition in a community repository (e.g. GitHub). See the Nature Portfolio guidelines for submitting code & software for further information.

Data

Policy information about availability of data

All manuscripts must include a data availability statement. This statement should provide the following information, where applicable:

- Accession codes, unique identifiers, or web links for publicly available datasets
- A description of any restrictions on data availability
- For clinical datasets or third party data, please ensure that the statement adheres to our policy

The Supporting Information provides supplementary figures and tables referenced in the text, the synthetic methods and characterization, DNA and protein sequence information for coding regions, plasmid sequences, and references relevant to this information. Additionally, the DNA and protein sequences including primer sequences are provided in a supplementary xlsx file. The source data for the main text figures and extended data figures are provided in individual xlsx files. The sequence data, source data, raw data, crystallographic data, and code are available on Zenodo (10.5281/zenodo.18271154). Additionally, crystallographic data for ScSLAC M298C6a has been deposited as PDB 9HU7 and is available on proteindiffraction.org. All reported plasmid sequences are deposited on GenBank, and the IDs are referenced in Methods and Supplementary Information. The plasmids pGS1T-PylRSC6a-m15PylTCUA, pGS1T-PylRSC6a-MmtRNAPylCUA, or pET28-ScSLAC-6xHis are available through Addgene (ID: 251554, 251555, and 251556, respectively).

Human research participants

Policy information about studies involving human research participants and Sex and Gender in Research.

Reporting on sex and gender	no research on human subjects was conducted
Population characteristics	no research on human subjects was conducted
Recruitment	no research on human subjects was conducted
Ethics oversight	no research on human subjects was conducted

Note that full information on the approval of the study protocol must also be provided in the manuscript.

Field-specific reporting

Please select the one below that is the best fit for your research. If you are not sure, read the appropriate sections before making your selection.

☒ Life sciences ☐ Behavioural & social sciences ☐ Ecological, evolutionary & environmental sciences

For a reference copy of the document with all sections, see nature.com/documents/nr-reporting-summary-flat.pdf

Life sciences study design

All studies must disclose on these points even when the disclosure is negative.

Sample size	The sample size varied between three and 600 depending on the type of analysis. For experiments where only metrics from descriptive statistics were used (e.g. mean, median, standard deviation, fold-change), the sample sizes were often small. Higher sample sizes were often used for regression analysis, and the sample size increased with increasing model complexity (example: linear calibration curves had sample sizes of approximately 6, whereas each composite regression analysis has a sample size of approximately 600). The sample sizes were deemed appropriate based on common practice in the field.
Data exclusions	There are no data exclusions.
Replication	The type and number of replicates for each experiment is indicated in the figure legend or provided in the methods section. For enzymatic characterization and aaRS/tRNA evaluation, the number of biological replicates ranged from 3-6.
Randomization	No randomization was performed as is common practice in the field.
Blinding	No blinding was performed. The data were analyzed in a uniform way using standardized methods to address analysis bias.

Reporting for specific materials, systems and methods

We require information from authors about some types of materials, experimental systems and methods used in many studies. Here, indicate whether each material, system or method listed is relevant to your study. If you are not sure if a list item applies to your research, read the appropriate section before selecting a response.

Materials & experimental systems

n/a	Involved in the study
☒	☐ Antibodies
☒	☐ Eukaryotic cell lines
☒	☐ Palaeontology and archaeology
☒	☐ Animals and other organisms
☒	☐ Clinical data
☒	☐ Dual use research of concern

Methods

n/a	Involved in the study
☒	☐ ChIP-seq
☒	☐ Flow cytometry
☒	☐ MRI-based neuroimaging
